# A Copper-Mediated Radical α-Heteroarylation of Nitriles with Azobis(alkylcarbonitriles)

**DOI:** 10.1021/acs.orglett.3c03727

**Published:** 2024-02-05

**Authors:** Gustavo G. Flores-Bernal, Luis D. Miranda

**Affiliations:** Department of Organic Chemistry, Instituto de Química, Universidad Nacional Autónoma de México, Circuito Exterior, Ciudad Universitaria, 04510 Mexico City, Mexico

## Abstract

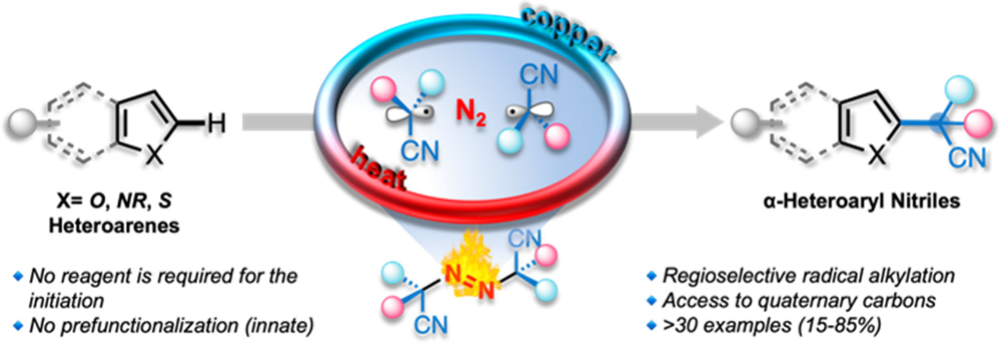

A new practical method has been developed for the α-heteroarylation of aliphatic nitriles with heteroarenes and azobis(alkylcarbonitriles) using Cu(OAc)_2_ as an oxidizing agent. This method allows the easy construction of nitrile-, aryl-, and dialkyl-bearing quaternary carbon centers from readily available building blocks, without requiring prefunctionalization steps. This reaction is based on adding cyanodialkyl radicals onto heteroarenes, including benzofurans, furans, pyrroles, and indoles. The resulting α-heteroaryl nitriles are useful synthetic intermediates and pharmacophores in biologically active molecules.

α-Aryl or heteroaryl dialkyl nitriles/esters are particularly important motifs, as these appear in bioactive molecules (Figure 1a) such as dactolisib (anticancer drug), verapamil (calcium-channel blocker), and alkaloids such as coronaridine.^1,2^ The nitrile group is considered a vital pharmacophore^3^ and a valuable synthetic intermediate. Its reactivity allows its transformation into various functional groups, such as esters, amides, ketones, aldehydes, carboxylic acids, amines, and nitrogen-containing heterocycles.^4^ The retrosynthetic analysis to obtain α-aryl dialkyl nitriles is based on the classic α-deprotonation and subsequent reaction with aryl and/or alkyl halides; however, strong bases are needed, decreasing the functional group tolerance of the process.^5^ Transition metal-catalyzed cross-coupling reactions of arene derivatives and nucleophilic nitriles have extended the synthetic repertoire.^6^ However, the required catalysts and ligands are often not readily available (Figure 1b). In general, previous approaches have mainly focused on obtaining secondary alkyl nitriles, very few methods have addressed the construction of sterically hindered α,α-dialkyl-α-aryl nitriles, and even fewer attempts have tackled the challenge of constructing the quaternary all-carbon center through direct C–H alkylation of heteroarenes.^1^ In this context, protocols that rely on the innate reactivity of the aromatic system, such as electrophilic and homolytic aromatic substitution, offer a practical method for functionalizing a C–H bond directly. This approach helps avoid unnecessary prefunctionalization steps, improving the atom and step economy of the synthetic scheme.

**Figure 1:**
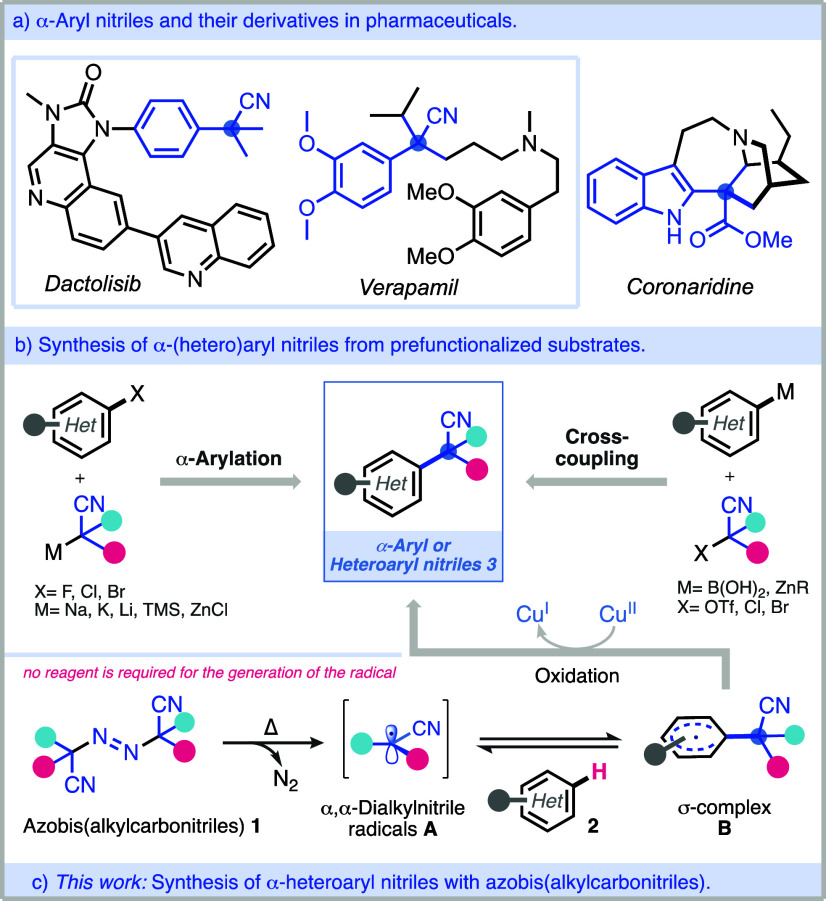
Approaches for the synthesis of α-heteroaryl nitriles.

The addition of a radical to an aromatic system poses two main challenges (Figure 1c). First, it is a relatively high-energy process because the aromatic system is disrupted. Second, this step can be highly reversible, especially when a stabilized radical **A** is involved. Therefore, an efficient oxidation system must be found for radical **B** to evolve into the rearomatized product.

Our interest in the addition of a radical to aromatic systems^7^ has recently led us to explore using azobis(alkylcarbonitriles) **1** as radical precursors for this process. This functional group has unique properties because it cleanly fragments into a pair of alkyl radicals and a nitrogen molecule. The process requires only heating a solution of **1**, and the temperature can be adjusted to control the half-life. For instance, several reports have demonstrated that azobis(alkylcarbonitirles) **1** are suitable radical precursors for functionalizing π-systems such as alkenes,^8^ alkynes,^9^ isocyanides,^10^ and aldehydes.^11^ Efforts have also been made toward the group-directed C(sp^2^)–H alkylation using azo initiators.^12−14^ Notably, to the best of our knowledge, the direct C(sp^2^)–H functionalization of heteroarenes using azobis(alkylcarbonitriles) **1** (Figure 1c) has not been explored. In this study, we present a copper-mediated radical α-heteroarylation method using azobis(alkylcarbonitriles) to synthesize α,α-dialkyl-α-aryl nitriles.

After a thorough evaluation of various potential coupling partners, the reaction between the cyanocyclohexyl radical derived from 1,1′-azobis(cyclohexanecarbonitrile) (**1a**) and 2,3-benzofuran (**2a**) was selected as a useful model system. The results are listed in Table 1.

**Table 1:**
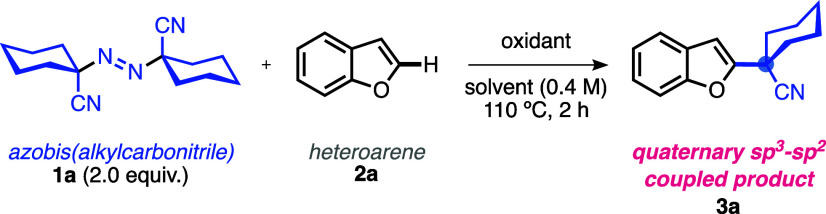
Optimization of the Reaction Conditions^a^

entry	oxidant (equiv)	solvent	yield of **3a** (%)^b^
1	Cu(OAc)_2_ (1.0)	TCE	73 (61)^c^
2	–	TCE	0
3	FeCl_3_ (1.0)	TCE	0
4	MnO_2_ (1.0)	TCE	0
5	(NH_4_)_2_S_2_O_8_ (1.0)	TCE	10
6	Cu(EH)_2_ (1.0)	TCE	69
7	CuCl_2_ (1.0)	TCE	44
8	Cu(acac)_2_ (1.0)	TCE	44
9	Cu(OTf)_2_ (1.0)	TCE	0
10	Cu(OAc)_2_ (1.0)	1,4-dioxane	2
11	Cu(OAc)_2_ (1.0)	DMF	20
12	Cu(OAc)_2_ (1.0)	2-methyl-2-butanol	20
13	Cu(OAc)_2_ (1.0)	*n*-PrOH	22
14	Cu(OAc)_2_ (1.0)	*n*-BuOH	29
15	Cu(OAc)_2_ (0.1)/(NH_4_)_2_S_2_O_8_ (1.0)	TCE	27
16	Cu(OAc)_2_ (0.1)/air	TCE	47
17	Cu(OAc)_2_ (0.1)/O_2_ (1 atm)	TCE	26

a Conditions: **1a** (0.8 mmol, 2.0 equiv), **2a** (0.4 mmol, 1.0 equiv), oxidant (0.4 mmol, 1.0 equiv, unless otherwise noted), 110 °C, 2 h.

b Isolated yield.

c With 1 mmol of **2a**. Abbreviations: TCE, 2,2,2-trichloroethanol; Cu(EH)_2_, copper(II) 2-ethylhexanoate; DMF, *N*,*N*-dimethylformamide.

Our experiment revealed that when we subjected **1a** to thermal cleavage using 1.0 equiv of Cu(OAc)_2_ as an oxidizing agent in a 2,2,2-trichloroethanol (TCE) solvent, we successfully obtained α-heteroarylation product **3a** in 73% yield. The reaction showed complete regioselectivity for position C-2 of the heteroarene and did not require any additives, bases, activating agents, or extended reaction times. The presence of Cu(OAc)_2_ was found to be crucial for the reaction as no product was obtained in its absence (Table 1, entry 2). Other oxidizing agents, which are usually used in similar radical alkylation processes, had a negative impact on reactivity (Table 1, entries 3–5). Evaluation of different Cu(II) sources showed that Cu(OAc)_2_ was better than CuCl_2_, Cu(acac)_2_, and Cu(OTf)_2_ (Table 1, entries 7–9, respectively), whereas copper(II) 2-ethylhexanoate [Cu(EH)_2_] (Table 1, entry 6) showed activity similar to that of Cu(OAc)_2_. As the thermal decomposition of azobis(alkylcarbonitriles) **1** follows a first-order kinetics,^15^ the reaction temperature determines the half-life (*t*_1/2_) and consequently the reaction time. In this case, after 2 h, solvents with boiling points of ≥100 °C completely decomposed **1a**. The evaluation of selected polar solvents showed that TCE was essential for coupling success. Table 1 shows that when other polar solvents like 1,4-dioxane, *N*,*N*-dimethylformamide (DMF), or alcohols were used, the yields were poorer (entries 10–14). Even though solvents did not significantly affect the decomposition rates of azo initiators,^16^ they still play a crucial role in cage effects, diffusion rates, and radical stabilization and trapping. Indeed, perhalogenated solvents have demonstrated significant usefulness in difficult C–H functionalizations.^17^ Because TCE is cheaper and has a boiling point higher than that of either hexafluoroisopropanol (HFIP) or trifluoroethanol (TFE), the conditions of entry 1 were determined to be optimal. Some assays carried out with a co-oxidant using catalytic amounts of Cu(OAc)_2_ considerably decreased the yield of **3a**.

Under the optimal reaction conditions, we examined various heteroarenes **2** and azobis(alkylcarbonitriles) **1**, affording 31 new α,α-dialkyl-α-aryl nitriles in 15–85% yields (Scheme 1). Both benzofurans (**3a**–**e**) and furans (**3f**–**l**) were suitable partners for the α-heteroarylation protocol with the cyanocyclohexyl radical derived from **1a**. The reaction was selective, and no regioisomers were obtained. The presence of halogen substituents in aromatic rings was well tolerated, as demonstrated in the bromine (**3b** and **3k**) and chlorine (**3j**) derivatives. In general, radical alkylation products on benzofurans and furans substituted with electron-withdrawing groups such as nitrile, ketone, ester, and nitro were obtained in lower yields (**3b**–**d**, **3h**–**j**, and **3l**) than benzofurans and furans substituted with alkyl groups (**3e**–**g**). To demonstrate the applicability of this protocol, the cyanocyclohexyl alkylation of bergapten (5-methoxypsoralen), a potent photochemotherapeutic agent, was achieved in 55% yield (**3m**). The yields of the synthesis of α,α-dialkyl-α-heteroaryl nitriles **3n**–**t** with **1a** and pyrrole, indole, and thiophene were the lowest (16–34%).

**Scheme 1:**
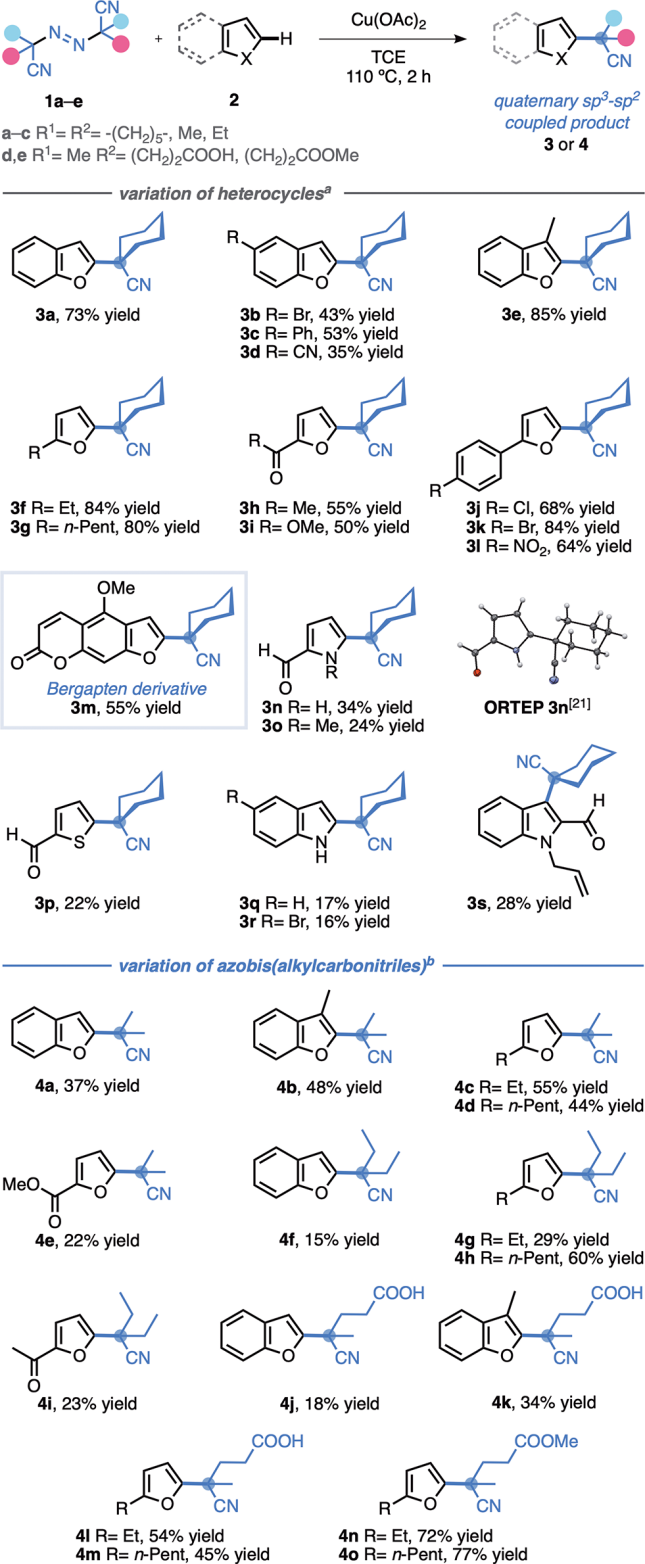
Scope of the α-Heteroarylation of Nitriles with Azobis(alkylcarbonitriles) **1** As in entry 1 of Table 1. Modified conditions: **1b**–**e** (0.8 mmol, 1.0 equiv), **2** (2.4 mmol, 3.0 equiv), Cu(OAc)_2_ (0.8 mmol, 1.0 equiv), TCE (0.8 M), 90 °C, 2 h.

Reaction with 2,2′-azobis(2-methylpropionitrile) (**1b**) and 2,2′-azobis(2-ethylbutanenitrile) (**1c**) produced α,α-dimethyl- and α,α-diethyl-α-(benzo)furanyl nitriles **4a**–**i**, at yields ranging from 15% to 60%. It is worth noting that the reaction can occur in the presence of a free carboxylic acid group. This was observed when 4,4′-azobis(4-cyanovaleric acid) (**1d**) was used, leading to the assembly of 4-[(benzo)furanyl]-4-cyano pentanoic acids **4j**–**m**, with yields ranging from 18% to 54%. Trapping these radicals has been a challenge in previous reports.^12,18^ Using 4,4′-azobis(4-cyanovaleric acid)dimethyl ester (**1e**) resulted in the formation of esters **4n** and **4o** with high yields (72–77%).

To further extend the methodology, we next examined the intramolecular cyclization reaction for the construction of a pyrido[1,2-*a*]indole skeleton bearing a quaternary all-carbon center from indole-containing azobis(alkylcarbonitriles) **5a**–**c** (Scheme 2). Compounds **5a**–**c** were synthesized through the N-acylation of indole with 4,4′-azobis(4-cyanovaleric acid) (**1d**). To our delight, in a less concentrated reaction medium (0.1 M) than the intermolecular radical addition, the thermolysis of azo compounds **5a**–**c** in the presence of Cu(OAc)_2_ (2.0 equiv) gave cyclized products **6a**–**c**, respectively, in 48–85% yields. Our laboratory is currently investigating the potential of this promising intramolecular cyclization reaction to synthesize valuable scaffolds for *Aspidosperma* alkaloids.^19^

**Scheme 2:**
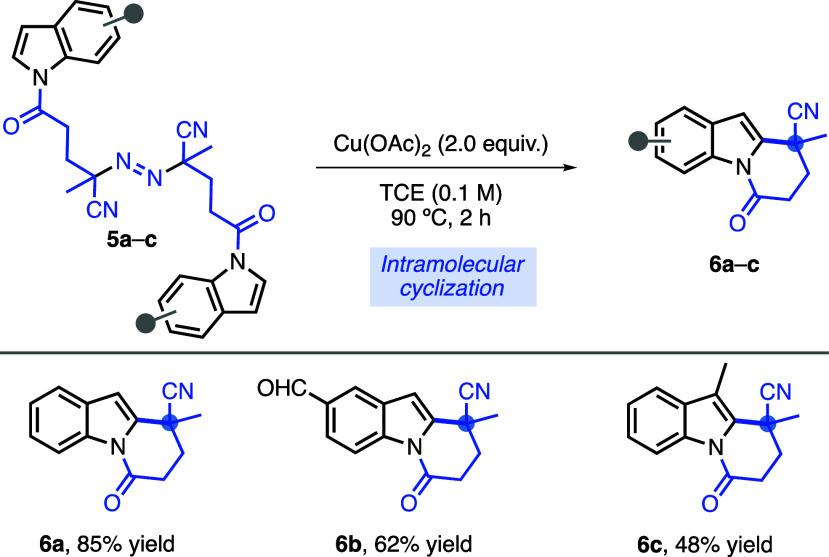
Intramolecular Approach

The protocol yields versatile synthetic intermediates, α,α-dialkyl-α-heteroaryl nitriles, which can be converted into important derivatives (Scheme 3). For instance, hydrogenation of the nitrile to the primary amine and *in situ* protection with Boc_2_O gave **7** in 73% yield. Similarly, the reduction of benzofuran-containing nitrile **3a** with DIBAL-H furnished the corresponding aldehyde **8** in 77% yield. It was possible to convert nitrile into ketone **9** with a 34% yield by using MeMgBr in toluene. The same substrate was also transformed into tetrazole derivative **10** in excellent yield using microwave irradiation.

**Scheme 3:**
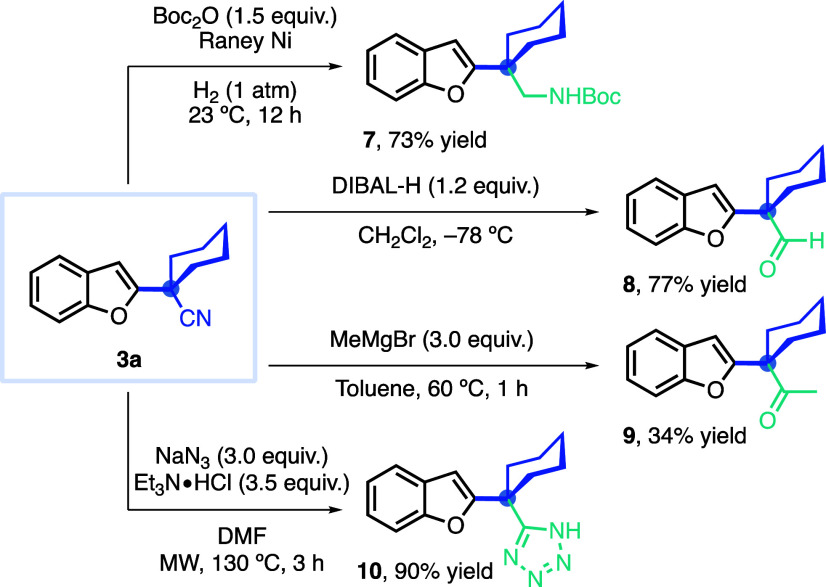
Derivatization of α-Heteroaryl Nitrile **3a**

During radical trapping experiments, either 2,6-di-*tert*-butylphenol or BHT was used as the radical scavenger in the presence of stoichiometric Cu(OAc)_2_ without heteroarene (Scheme 4). As a result, cyanoalkyl radicals were added directly to the aromatic ring, leading to the formation of phenols **11a** and **11b** (64% and 81% yields, respectively) and dearomatized products **12a** and **12b** (73% and 92% yields, respectively).

**Scheme 4:**
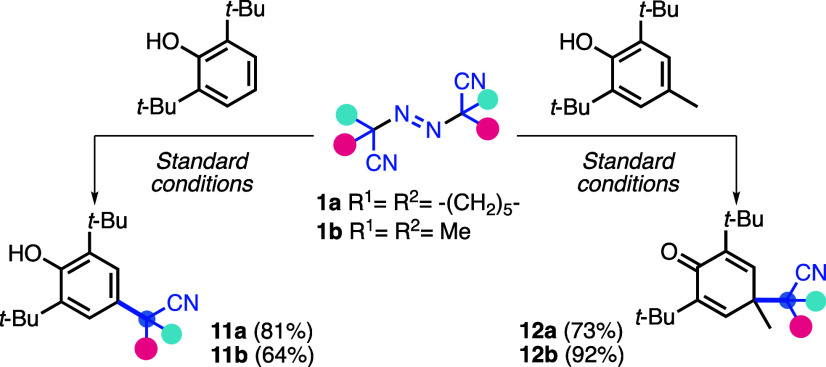
Radical Trapping Experiments

In the proposed reaction mechanism (Scheme 5) after the thermal decomposition of **1**, a pair of cyanoalkyl radicals **A** are formed along with a nitrogen molecule. Then, direct addition of radical **2** affords the intermediate radical (σ-complex **B**) in a reversible process whose progress is favored mainly by the strength of the new bond formed, the aromaticity of the system (enthalpy effect), and the electronic match of the heteroarene–alkyl radical pair (polar effect).^20^ Then, copper-mediated oxidative radical–polar crossover provides the corresponding carbocation **C**, and the subsequent loss of a proton regenerates the aromaticity to afford α-heteroaryl nitrile **3**.

**Scheme 5:**
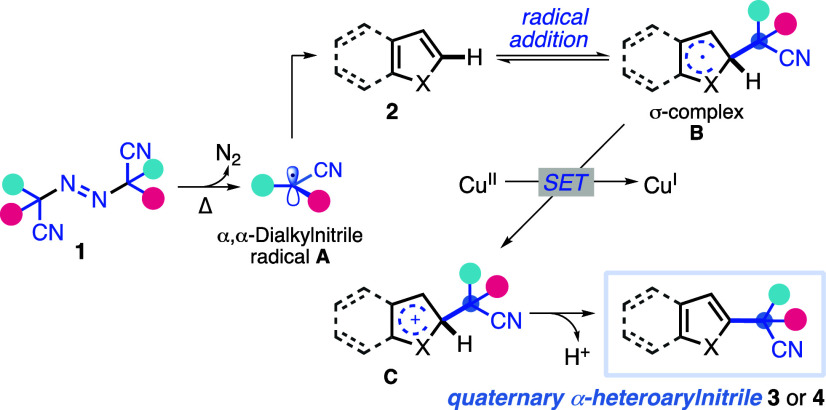
Proposed Reaction Mechanism

In summary, we have developed a practical protocol for the α-heteroarylation of aliphatic nitriles with non-prefunctionalized heteroarenes using readily available Cu(OAc)_2_ and commercially available azobis(alkylcarbonitriles). The success of this method relies on generating cyanoalkyl radicals by thermolysis of the azobis(alkylcarbonitriles), releasing N_2_ as the only waste product. The direct copper-mediated radical cyanoalkylation of heteroarenes offers a new method for synthesizing valuable sterically hindered α,α-dialkyl-α-aryl nitriles under mild conditions and short reaction times. The process is highly regioselective and compatible with diverse functional groups. Our work contributes to the growing demand for the direct introduction of quaternary all-carbon centers into heteroarenes, which could be of great interest to the scientific community working in the field of organic synthesis.

## Data Availability

The data underlying this study are available in the published article and its Supporting Information.

## Supporting Information Available

Experimental procedures, ^1^H and ^13^C NMR spectra of products, and X-ray crystallographic information (PDF)

## References

[ref1] aVitaku E.; Smith D. T.; Njardarson J. T. Analysis of the Structural Diversity, Substitution Patterns, and Frequency of Nitrogen Heterocycles among U.S. FDA Approved Pharmaceuticals. J. Med. Chem. 2014, 57, 10257–10274. 10.1021/jm501100b. 25255204

[ref2] Fleming F. F. Nitrile-containing natural products. Nat. Prod. Rep. 1999, 16, 597–606. 10.1039/a804370a.

[ref3] Fleming F. F.; Yao L.; Ravikumar P. C.; Funk L.; Shook B. C. Nitrile-Containing Pharmaceuticals: Efficacious Roles of the Nitrile Pharmacophore. J. Med. Chem. 2010, 53, 7902–7917. 10.1021/jm100762r. 20804202 PMC2988972

[ref4] Rappoport Z. In The Cyano Group. Patai’s Chemistry of Functional Groups; Patai S., Ed.; John Wiley & Sons: London, 1970.

[ref5] aKlapars A.; Waldman J. H.; Campos K. R.; Jensen M. S.; McLaughlin M.; Chung J. Y. L.; Cvetovich R. J.; Chen C.-Y Mild and Practical Method for the α-Arylation of Nitriles with Heteroaryl Halides. J. Org. Chem. 2005, 70, 10186–10189. 10.1021/jo051737f. 16292870

[ref6] aHe A.; Falck J. R. Stereospecific Suzuki Cross-Coupling of Alkyl α-Cyanohydrin Triflates. J. Am. Chem. Soc. 2010, 132, 2524–2525. 10.1021/ja910582n. 20121273 PMC2893341

[ref7] aOsornio Y. M.; Cruz-Almanza R.; Jiménez-Montaño V.; Miranda L. D. Efficient, intermolecular, oxidative radical alkylation of heteroaromatic systems under “tin-free” conditions. Chem. Commun. 2003, 2316–2317. 10.1039/B306966D. 14518892

[ref8] Cao Z.-Z.; Nie Z.; Yang T.; Su M.; Li H.; Luo W.-P.; Liu Q.; Guo C.-C. Metal-Free-Catalyzed Synthesis of Allyl Nitriles via C_sp2_–C_sp3_ Coupling between Olefins and Azobis (Alkyl-carbonitrile). J. Org. Chem. 2020, 85, 3287–3296. 10.1021/acs.joc.9b03127. 31944119

[ref9] aRong G.; Mao J.; Zheng Y.; Yao R.; Xu X. Cu-Catalyzed direct cyanation of terminal alkynes with AMBN or AIBN as the cyanation reagent. Chem. Commun. 2015, 51, 13822–13825. 10.1039/C5CC04987C. 26235725

[ref10] Song W.; Yan P.; Shen D.; Chen Z.; Zeng X.; Zhong G. Synthesis of Cyano-Containing Phenanthridine Derivatives via Catalyst-, Base-, and Oxidant-Free Direct Cyanoalkylarylation of Isocyanides. J. Org. Chem. 2017, 82, 4444–4448. 10.1021/acs.joc.7b00343. 28362091

[ref11] Wang J.-M.; Chen T.; Yao C.-S.; Zhang K. Synthesis of β-Ketonitriles via *N*-Heterocyclic-Carbene-Catalyzed Radical Coupling of Aldehydes and Azobis(isobutyronitrile). Org. Lett. 2023, 25, 3325–3329. 10.1021/acs.orglett.3c01168. 37104729

[ref12] Wen C.; Zhong R.; Qin Z.; Zhao M.; Li J. Regioselective remote C5 cyanoalkoxylation and cyanoalkylation of 8-aminoquinolines with azobisisobutyronitrile. Chem. Commun. 2020, 56, 9529–9532. 10.1039/D0CC00014K. 32687138

[ref13] Zhao M.; Qin Z.; Zhang K.; Li J. Metal-free site-selective C–H cyanoalkylation of 8-aminoquinoline and aniline-derived amides with azobisisobutyronitrile. RSC. Adv. 2021, 11, 30719–30724. 10.1039/D1RA06013A. 35479854 PMC9041112

[ref14] Yang S.; Yan B.; Zhong L.; Jia C.; Yao D.; Yang C.; Sun K.; Li G. AIBN for Ru-catalyzed *meta*-C_Ar_–H alkylation. Org. Chem. Front. 2020, 7, 2474–2479. 10.1039/D0QO00703J.

[ref15] FUJIFILM Wako Chemical Corp. Azo Radical Initiators. https://www.fujifilm.com/ffwk/en and https://labchem-wako.fujifilm.com/us/category/docs/azo%20initiators%20catalog.pdf (accessed October 2023).

[ref16] aLewis F. M.; Matheson M. S. Decomposition of Aliphatic Azo Compounds. J. Am. Chem. Soc. 1949, 71, 747–748. 10.1021/ja01170a513.

[ref17] aYu C.; Sanjosé-Orduna J.; Patureau F. W.; Pérez-Temprano M. H. Emerging unconventional organic solvents for C–H bond and related functionalization reactions. Chem. Soc. Rev. 2020, 49, 1643–1652. 10.1039/C8CS00883C. 32115586

[ref18] aTang S.; Zhou D.; Li Z.-H.; Fu M.-J.; Jie L.; Sheng R.-L.; Li S.-H. Azo-Compound-Mediated Cyanoalkylation of Alkenes by Copper Catalysis: General Access to Cyano-Substituted Oxindoles. Synthesis 2015, 47, 1567–1580. 10.1055/s-0034-1379902.

[ref19] aZhang J.; Han F.-S. A Total Synthesis of (±)-Leuconodines D and E. J. Org. Chem. 2019, 84, 13890–13896. 10.1021/acs.joc.9b02054. 31535552

[ref20] Fischer H.; Radom L. Factors Controlling the Addition of Carbon-Centered Radicals to Alkenes—An Experimental and Theoretical Perspective. Angew. Chem., Int. Ed. 2001, 40, 1340–1371. 10.1002/1521-3773(20010417)40:8<1340::AID-ANIE1340>3.0.CO;2-#. 11317286

