# Supplementary material for: A Copper-Mediated Radical α-Heteroarylation of Nitriles with Azobis(alkylcarbonitriles)
Source: Org Lett. 2024 Feb 5;26(6):1128–33. doi: 10.1021/acs.orglett.3c03727 (PMC10877592; doi:10.1021/acs.orglett.3c03727)
Supplement: Supplementary file 1 — ol3c03727_si_001.pdf [file ol3c03727_si_001.pdf]

Supporting Information for

A Copper-Mediated Radical α-Heteroarylation of Nitriles with Azobis(alkylcarbonitriles)

Gustavo G. Flores-Bernal and Luis D. Miranda*

Instituto de Química, Universidad Nacional Autónoma de México, Circuito Exterior, Ciudad Universitaria, 04510 Mexico City, Mexico.

*E-mail: lmiranda@unam.mx; Tel: +52-55-5622-4420

Table of Contents

Part I Experimental section S2
General information S2
1.1 Procedure for the synthesis of substrates S2
1.2 Procedure for the synthesis of α-heteroaryl nitriles **3** S3
1.3 Procedure for the synthesis of α-heteroaryl nitriles **4** S7
1.4 Procedure for the intramolecular cyclization of indole-containing azobis(alkylcarbonitriles) **5** for the construction of pyrido[1,2-*a*]indolones **6** S10
1.5 Derivatization of 1-(benzofuran-2-yl)cyclohexane-1-carbonitrile (**3a**) S12
1.6 Radical trapping experiments S13
1.7 X-ray crystallographic information of **3n** S14
Part II ^{1}H and ^{13}C NMR spectra S15
References S65

Part I Experimental section

General information

^{1}H and ^{13}C NMR spectra were obtained on JEOL Eclipse 300 MHz, Bruker Avance III 400 MHz, Bruker Avance III HD 500 MHz and 700 MHz spectrometers. Chemical shifts (δ) are reported in parts per million (ppm) relative to residual proton signal of $CHCl_3$ (δ 7.26) for ^{1}H NMR, and $CDCl_3$ (δ 77.0) for ^{13}C NMR. Coupling constants (J) are reported in Hertz. Peak assignments of the ^{1}H and ^{13}C NMR spectra were confirmed by using 2D NMR experiments (COSY, TOCSY, HSQC, and HMBC). HRMS were determined on a JEOL AccuTOF JMS-T100LC with an ionSense DART controller ionization source or an Agilent 6530 Accurate-Mass Q-TOF LC/MS spectrometer, as specified. Infrared spectra were recorded on a Bruker Tensor 27 FT-IR spectrophotometer. X-ray crystallographic structure for **3n** was obtained on a Bruker D8 Venture diffractometer using CuKα radiation (λ= 1.5417 Å). Microwave-assisted reactions were performed using a Microwave Synthesis System-CEM reactor. Melting points were determined on a Fisher apparatus and are uncorrected. Thin layer chromatograms were performed on precoated TLC sheets of silica gel 60 F_{254} (E. Merck). Flash chromatography was carried out using silica gel (Merck 230–400 mesh). All reactions were carried out under an argon atmosphere in oven- or flame-dried glassware unless the reaction procedure states otherwise. Degassed solutions were obtained by freeze-pump-thaw cycles (3x) using liquid nitrogen. Azobis(alkylcarbonitriles) **1a**,**b**,**d** were purchased from Sigma-Aldrich and were used without further purification. All reagents and solvents were purchased from Sigma-Aldrich and Tecsiquim and were used without further purification.

1.1 Procedure for the synthesis of substrates

Most heteroarenes were commercially available. 5-Phenylbenzofuran (**2c**) and 5-benzofurancarbonitrile (**2d**) were prepared from 5-bromobenzofuran (**2b**) by Suzuki cross-coupling with phenylboronic acid[1] and Rosenmund-Von Braun Aromatic Cyanation with CuCN,[2] respectively. 2-Phenylfurans **2j**–**l** were prepared by mechanoredox arylation of furan with aryldiazonium salts.[3] 1-(2-Propen-1-yl)-1*H*-indole-2-carboxaldehyde (**2s**) was prepared by alkylation of 1*H*-indole-2-carboxaldehyde with allyl bromide.[4] NMR spectra matched previously reported data.

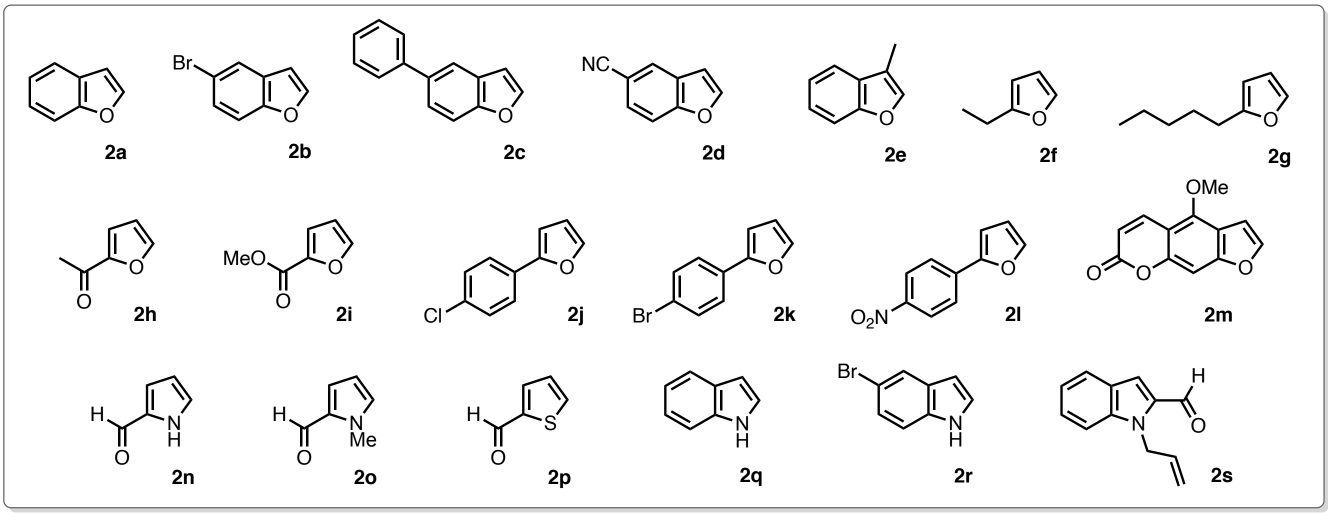

The azobis(alkylcarbonitriles) **1a**,**b**,**d** were commercially available. The azobis(alkylcarbonitriles) **1c**,**e** were prepared according to the reported procedure.[5]

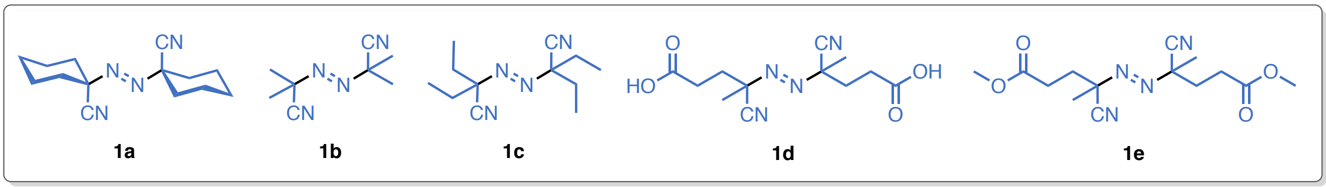

1.1.1 Synthesis of 2,2'-azobis(2-ethylbutanenitrile) (1c)

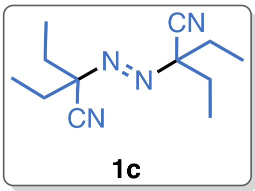

Following the procedure described by Zhang and co-workers.[5] (**Caution: The reaction must take place in a fume cupboard**). In a microwave reaction vial equipped with a stir bar and sealed with a PTFE lined butyl rubber septum and aluminum crimp cap, a mixture of 3-pentanone (0.86 g, 10.0 mmol) and hydrazine (64% wt. in water, 0.25 g, 5.0 mmol) was heated at 100 ºC in heating mantle for 5 min and cooled to room temperature. TMSCN (0.99 g, 10.0 mmol) was then added dropwise. After another 5 min stirring at 100 ºC in heating mantle, water was added and extracted with DCM. The organic phase was dried over anhydrous Na_2SO_4 and evaporated under vacuum to give the crude 1,2-bis(3-cyanobutyl) hydrazine as a yellow oil. The crude yellow oil was used to the next step without purification. To the stirred solution of the crude 1,2-bis(3-cyanobutyl) hydrazine (5.0 mmol) in DCM (25 ml) was added trichloroisocyanuric acid (TCICA) (0.395 g, 1.70 mmol) in small portions and stirring was continued for 1 h and then filtered on Celite, and the organic phase was washed with a saturated solution of

Na_2CO_3. The organic phase was dried over anhydrous Na_2SO_4, and the solvent was evaporated under vacuum at room temperature. The crude product was purified by flash column chromatography on silica gel with DCM as eluent to give **1c** (0.734 g, 3.33 mmol, 67%) as a white solid. mp: 65-65 ºC (dec.). **^{1}H NMR** (300 MHz, $CDCl_3$): δ 2.16-1.91 (m, 4H), 0.92 (t, *J* = 7.5 Hz, 1H). **^{13}C NMR** (75 MHz, $CDCl_3$): δ 116.9, 78.1, 29.8, 8.2. **FT-IR** (ATR) ν_{max}: 2975, 2937, 2884, 2852, 2241, 1462, 1444, 1381, 1349, 1319, 1186, 1154, 1063, 1038, 952, 918, 765, 678, 633, 588, 474, 455 cm^{-1}. **HRMS**-(ESI) (*m*/*z*) calcd for $C_{12}H_{20}N_4Na$ $[M+Na]^+$: 243.1586; found: 243.1587. Spectroscopic data are in accordance with those described in the literature.

1.1.2 Synthesis of 4,4'-azobis(4-cyanovaleric acid)dimethyl ester (1e)

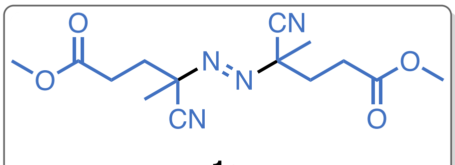

1e

Following the procedure described by Zhang and co-workers.[5] $TMSCHN_2$ (2.0 M in hexane, 5.0 mL, 10.05 mmol) was slowly added to a solution of 4,4'-azobis(4-cyanovaleric acid) (0.42 g, 1.5 mmol) in methanol (0.2 M) at 0 °C. After 0.5 h stirring at 0 °C, acetic acid (1.0 mL) was added. The reaction mixture was then evaporated under vacuum at room temperature and the residue was purified by flash column chromatography on silica gel with hexane/acetone (8:2) as eluent to afford **1e** (0.394 g, 1.278 mmol, 85%) as colorless crystals. mp: 60-65 °C (dec.). **^{1}H NMR** (300 MHz, $CDCl_3$): δ 3.69 (s, 3H), 3.68 (s, 3H), 2.56-2.28 (m, 8H), 1.70 (s, 3H), 1.65 (s, 3H). **^{13}C NMR** (75 MHz, $CDCl_3$): δ 171.7, 171.7, 117.4, 117.4, 71.8, 71.7, 52.0, 33.1, 33.1, 28.9, 28.8, 23.8, 23.6. **FT-IR** (ATR) ν_{max}: 2993, 2952, 2852, 2242, 1726, 1438, 1383, 1325, 1298, 1207, 1180, 1126, 987, 890, 802, 772, 686, 607, 481, 452, 416 cm^{-1}. **HRMS**-(ESI) (*m*/*z*) calcd for $C_{14}H_{21}N_4O_4$ $[M+H]^+$: 309.1563; found: 309.1560. Spectroscopic data are in accordance with those described in the literature.

1.2 Procedure for the synthesis of α-heteroaryl nitriles 3

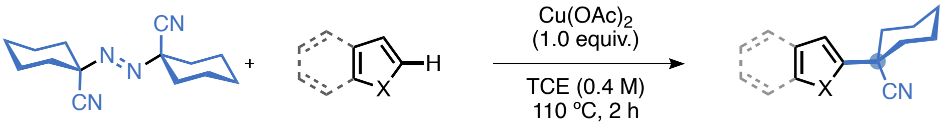

1a (2.0 equiv.) **2** (1.0 equiv.) **3**

In a microwave reaction vial equipped with a stir bar was charged with 1,1'-azobis(cyclohexanecarbonitrile) (**1a**) (2.0 equiv.), heteroarene **2** (1.0 equiv.), $Cu(OAc)_2$ (1.0 equiv.) and 2,2,2-trichloroethanol (TCE) (0.4 M). The vial was sealed with a PTFE lined butyl rubber septum and aluminum crimp cap, and the solution was degassed by three consecutive freeze−pump−thaw cycles using liquid nitrogen and backfilled with pure argon. The mixture was stirred at 110 ºC in heating mantle for 2 h. After cooling to room temperature, the crude reaction mixture was extracted with a saturated solution of $NaHCO_3$ and DCM. The organic phase was dried over anhydrous Na_2SO_4 and evaporated under reduced pressure. The residue was purified by flash column chromatography on silica gel to afford the desired α-heteroaryl nitrile **3**.

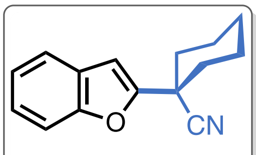

3a

1-(Benzofuran-2-yl)cyclohexane-1-carbonitrile (3a) (1 mmol scale). In a microwave reaction vial equipped with a stir bar was charged with 1,1'-azobis(cyclohexanecarbonitrile) (**1a**) (500 mg, 2.0 mmol), 2,3-benzofuran (**2a**) (0.11 mL, 1.0 mmol), $Cu(OAc)_2$ (185 g, 1.0 mmol) and 2,2,2-trichloroethanol (TCE) (2.4 mL). The vial was sealed with a PTFE lined butyl rubber septum and aluminum crimp cap, and the solution was degassed by three consecutive freeze−pump−thaw cycles using liquid nitrogen and backfilled with pure argon. The mixture was stirred at 110 ºC in heating mantle for 2 h. After cooling to room temperature, the crude reaction mixture was extracted with a saturated solution of $NaHCO_3$ (40 mL) and DCM (50 mL). The organic phase was dried over anhydrous Na_2SO_4 and evaporated under reduced pressure. The residue was purified by flash column chromatography on silica gel with hexane/DCM (65:35) as eluent to afford the desired nitrile **3a** (137 mg, 0.608 mmol, 61%) as a yellow oil.

1-(Benzofuran-2-yl)cyclohexane-1-carbonitrile (3a). Following the general procedure above, using 2,3-benzofuran (**2a**) (45 µL, 0.41 mmol), 1,1'-azobis(cyclohexanecarbonitrile) (**1a**) (204 mg, 0.82 mmol), $Cu(OAc)_2$ (76 mg, 0.41 mmol) and 2,2,2-trichloroethanol (TCE) (1.0 mL), the crude reaction mixture was purified by flash column chromatography on silica gel with hexane/DCM (65:35) as eluent to afford the desired compound **3a** (67 mg, 0.297 mmol, 73%) as a yellow oil. **^{1}H NMR** (400 MHz, $CDCl_3$): δ 7.56 (d, *J* = 7.5 Hz, 1H), 7.48 (dd, *J* = 8.0, 0.6 Hz, 1H), 7.30 (td, *J* = 7.2, 1.2 Hz, 1H), 7.24 (td, *J* = 7.7, 1.2 Hz, 1H), 6.70 (d, *J* = 0.8 Hz, 1H), 2.34 (d, *J* = 13.1 Hz, 2H), 1.99 – 1.74 (m, 8H). **^{13}C NMR** (100 MHz, $CDCl_3$): δ 156.1, 154.8, 127.7, 124.6, 123.1, 121.1, 120.6, 111.3, 102.6, 39.8, 34.6, 24.9, 22.7. **FT-IR** (film) ν_{max}: 2937, 2861, 2236, 1583, 1473, 1453, 1351, 1309, 1255, 1185, 1168, 1107, 1021, 956, 877, 808, 751, 680, 452 cm^{-1}. **HRMS**-(DART) (*m*/*z*) calcd for $C_{15}H_{16}NO$ $[M+H]^+$: 226.1232; found: 226.1231.

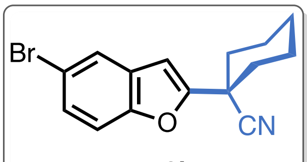

3b

1-(5-Bromobenzofuran-2-yl)cyclohexane-1-carbonitrile (3b). Following the general procedure above, using 5-bromobenzofuran (**2b**) (53 µL, 0.41 mmol), 1,1'-azobis(cyclohexanecarbonitrile) (**1a**) (204 mg, 0.82 mmol), $Cu(OAc)_2$ (76 mg, 0.41 mmol) and 2,2,2-trichloroethanol (TCE) (1.0 mL), the crude reaction mixture was purified by flash column chromatography on silica gel with hexane/DCM (65:35) as eluent to afford the desired compound **3b** (54 mg, 0.178 mmol, 43%) as a yellow oil. **^{1}H NMR** (400 MHz, $CDCl_3$): δ 7.68 (d, *J* = 1.9 Hz, 1H), 7.43 – 7.30 (m, 2H), 6.64 (d, *J* = 0.8 Hz, 1H), 2.32 (dd, *J* = 12.7, 1.6 Hz, 2H), 1.98 – 1.72 (m, 8H). **^{13}C NMR** (100 MHz, $CDCl_3$): δ 157.5, 153.6, 129.7, 127.6, 123.8, 120.3, 116.1, 112.8, 102.2, 39.8, 34.6, 24.8, 22.7. **FT-IR** (film) ν_{max}: 2937, 2860, 2237, 1725, 1645, 1611, 1590,

1444, 1351, 1258, 1245, 1185, 1159, 1118, 1049, 1022, 957, 940, 903, 869, 800, 672 cm^{-1}. **HRMS**-(DART) (*m*/*z*) calcd for $C_{15}H_{15}BrNO$ [M+H]$^+$: 304.0337; found: 304.0342.

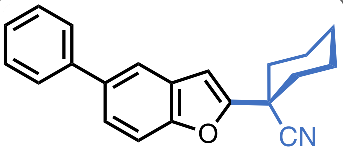

3c

1-(5-Phenylbenzofuran-2-yl)cyclohexane-1-carbonitrile (3c). Following the general procedure above, using 5-phenylbenzofuran (**2c**) (81 mg, 0.417 mmol), 1,1'-azobis(cyclohexanecarbonitrile) (**1a**) (208 mg, 0.834 mmol), $Cu(OAc)_2$ (77 mg, 0.417 mmol) and 2,2,2-trichloroethanol (TCE) (1.0 mL), the crude reaction mixture was purified by flash column chromatography on silica gel with hexane/DCM (65:35) as eluent to afford the desired compound **3c** (67 mg, 0.222 mmol, 53%) as a white solid. mp: 105-110 ºC. **^{1}H NMR** (400 MHz, $CDCl_3$): δ 7.74 (t, *J* = 1.3 Hz, 1H), 7.61 (dd, *J* = 8.4, 1.3 Hz, 2H), 7.53 (d, *J* = 1.3 Hz, 2H), 7.46 (t, *J* = 7.4 Hz, 2H), 7.36 (tt, *J* = 7.3, 1.4 Hz, 1H), 6.74 (s, 1H), 2.35 (d, *J* = 12.8 Hz, 2H), 2.01 – 1.74 (m, 8H). **^{13}C NMR** (100 MHz, $CDCl_3$): δ 156.7, 154.4, 141.4, 136.9, 128.8, 128.3, 127.4, 127.0, 124.3, 120.5, 119.6, 111.4, 102.8, 39.8, 34.6, 24.9, 22.7. **FT-IR** (ATR) ν_{max}: 2930, 2857, 2232, 1735, 1586, 1462, 1448, 1313, 1265, 1256, 1231, 1184, 1153, 1122, 1019, 951, 881, 842, 814, 761, 698, 629, 585, 524, 451 cm^{-1}. **HRMS**-(DART) (*m*/*z*) calcd for $C_{21}H_{20}NO$ [M+H]$^+$: 302.1545; found: 302.1539.

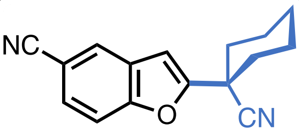

3d

2-(1-Cyanocyclohexyl)benzofuran-5-carbonitrile (3d). Following the general procedure above, using benzofuran-5-carbonitrile (**2d**) (60 mg, 0.418 mmol), 1,1'-azobis(cyclohexanecarbonitrile) (**1a**) (209 mg, 0.837 mmol), $Cu(OAc)_2$ (78 mg, 0.418 mmol) and 2,2,2-trichloroethanol (TCE) (1.0 mL), the crude reaction mixture was purified by flash column chromatography on silica gel with hexane/acetone (8:2) as eluent to afford the desired compound **3d** (37 mg, 0.148 mmol, 35%) as a yellow oil. **^{1}H NMR** (400 MHz, $CDCl_3$): δ 7.90 (d, *J* = 0.7 Hz, 1H), 7.61 – 7.53 (m, 2H), 6.77 (d, *J* = 0.8 Hz, 1H), 2.34 (dd, *J* = 12.7, 1.7 Hz, 2H), 1.97 – 1.73 (m, 8H). **^{13}C NMR** (100 MHz, $CDCl_3$): δ 158.7, 156.4, 128.4, 128.4, 126.2, 119.9, 119.1, 112.5, 107.2, 102.6, 39.8, 34.5, 24.7, 22.6. **FT-IR** (ATR) ν_{max}: 2948, 2934, 2859, 2225, 1731, 1589, 1466, 1451, 1267, 1201, 1178, 1143, 1121, 1020, 956, 937, 887, 827, 805, 736, 620, 546, 526, 489, 449 cm^{-1}. **HRMS**-(DART) (*m*/*z*) calcd for $C_{16}H_{15}N_2O$ [M+H]$^+$: 251.1184; found: 251.1174.

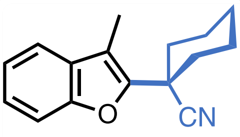

3e

1-(3-Methylbenzofuran-2-yl)cyclohexane-1-carbonitrile (3e). Following the general procedure above, using 3-methylbenzofuran (**2e**) (53 μL, 0.41 mmol), 1,1'-azobis(cyclohexanecarbonitrile) (**1a**) (204 mg, 0.82 mmol), $Cu(OAc)_2$ (76 mg, 0.41 mmol) and 2,2,2-trichloroethanol (TCE) (1.0 mL), the crude reaction mixture was purified by flash column chromatography on silica gel with hexane/DCM (65:35) as eluent to afford the desired compound **3e** (83 mg, 0.347 mmol, 85%) as an orange oil. **^{1}H NMR** (400 MHz, $CDCl_3$): δ 7.50 (dd, J = 7.7, 1.4 Hz, 1H), 7.41 (dd, J = 7.6, 1.3 Hz, 1H), 7.29 (td, J = 7.2, 1.4 Hz, 1H), 7.25 (td, J = 7.4, 1.4 Hz, 1H), 2.43 (s, 3H), 2.29 (dd, J = 13.2, 1.4 Hz, 2H), 2.12 – 2.02 (m, 2H), 1.93 – 1.75 (m, 6H). **^{13}C NMR** (100 MHz, $CDCl_3$): δ 153.0, 148.8, 130.4, 124.4, 122.5, 120.5, 119.3, 111.4, 110.8, 39.4, 34.7, 24.8, 22.7, 8.4. **FT-IR** (film) ν_{max}: 3063, 3040, 2937, 2861, 2235, 1934, 1893, 1766, 1689, 1606, 1589, 1476, 1453, 1389, 1343, 1266, 1245, 1193, 1156, 1106, 1007, 913, 903, 873, 845, 771, 746, 711, 674 cm^{-1}. **HRMS**-(DART) (*m*/*z*) calcd for $C_{16}H_{18}NO$ [M+H]$^+$: 240.1388; found: 240.1397.

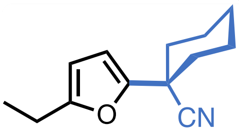

3f

1-(5-Ethylfuran-2-yl)cyclohexane-1-carbonitrile (3f). Following the general procedure above, using 2-ethylfuran (**2f**) (45 μL, 0.41 mmol), 1,1'-azobis(cyclohexanecarbonitrile) (**1a**) (204 mg, 0.82 mmol), $Cu(OAc)_2$ (76 mg, 0.41 mmol) and 2,2,2-trichloroethanol (TCE) (1.0 mL), the crude reaction mixture was purified by flash column chromatography on silica gel with hexane/DCM (8:2) as eluent to afford the desired compound **3f** (70 mg, 0.344 mmol, 84%) as a colorless oil. **^{1}H NMR** (400 MHz, $CDCl_3$): δ 6.12 (d, *J* = 3.2 Hz, 1H), 5.91 (dt, *J* = 3.2, 1.1 Hz, 1H), 2.62 (qd, *J* = 7.6, 1.1 Hz, 2H), 2.28 – 2.18 (m, 2H), 1.83 – 1.69 (m, 8H), 1.22 (t, *J* = 7.6 Hz, 3H). **^{13}C NMR** (100 MHz, $CDCl_3$): δ 157.9, 151.2, 121.2, 105.9, 104.5, 39.0, 34.7, 25.0, 22.6, 21.3, 11.9. **FT-IR** (ATR) ν_{max}: 2971, 2936, 2860, 2235, 1752, 1605, 1557, 1451, 1369, 1322, 1269, 1227, 1206, 1183, 1153, 1129, 1062, 1013, 963, 934, 902, 792, 779, 718, 574, 486 cm^{-1}. **HRMS**-(DART) (*m*/*z*) calcd for $C_{13}H_{18}NO$ [M+H]$^+$: 204.1388; found: 204.1386.

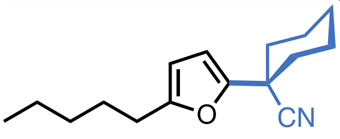

3g

1-(5-Pentylfuran-2-yl)cyclohexane-1-carbonitrile (3g). Following the general procedure above, using 2-pentylfuran (**2g**) (64 μL, 0.41 mmol), 1,1'-azobis(cyclohexanecarbonitrile) (**1a**) (204 mg, 0.82 mmol), $Cu(OAc)_2$ (76 mg, 0.41 mmol) and 2,2,2-trichloroethanol (TCE) (1.0 mL), the crude reaction mixture was purified by flash column chromatography on silica gel with hexane/DCM (8:2) as eluent to afford the desired compound **3g** (80 mg, 0.326 mmol, 80%) as an orange oil. **^{1}H NMR** (400 MHz, $CDCl_3$): δ 6.12 (d, *J* = 3.2 Hz, 1H), 5.91 (d, *J* = 3.1 Hz, 1H), 2.58 (t, *J* = 7.6 Hz, 2H), 2.28 – 2.17 (m, 2H), 1.83 – 1.69 (m, 8H), 1.67 – 1.57 (m, 2H), 1.36 – 1.29 (m, 4H), 0.90 (d, *J* = 7.0 Hz, 3H). **^{13}C NMR** (100 MHz, $CDCl_3$): δ 156.7, 151.2, 121.2, 105.9, 105.2, 39.0, 34.7, 31.3, 27.9, 27.5, 25.0, 22.7, 22.3, 14.0. **FT-IR** (film) ν_{max}: 2940, 2861, 2235, 1748, 1719, 1671, 1606, 1557, 1451, 1379, 1339, 1270, 1228, 1172, 1153, 1016, 967, 935, 903, 829, 784, 732 cm^{-1}. **HRMS**-(DART) (*m*/*z*) calcd for $C_{16}H_{24}NO$ [M+H]$^+$: 246.1857; found: 246.1864.

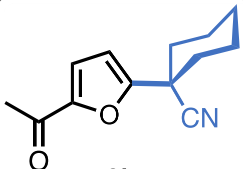

3h

1-(5-Acetylfuran-2-yl)cyclohexane-1-carbonitrile (3h). Following the general procedure above, using 2-furyl methyl ketone (**2h**) (75 μL, 0.41 mmol), 1,1'-azobis(cyclohexanecarbonitrile) (**1a**) (204 mg, 0.82 mmol), $Cu(OAc)_2$ (76 mg, 0.41 mmol) and 2,2,2-trichloroethanol (TCE) (1.0 mL), the crude reaction mixture was purified by flash column chromatography on silica gel with hexane/EtOAc (8:2) as eluent to afford the desired compound **3h** (49 mg, 0.226 mmol, 55%) as an orange oil. **^{1}H NMR** (400 MHz, $CDCl_3$): δ 7.13 (dd, *J* = 3.6, 1.0 Hz, 1H), 6.47 (dd, *J* = 3.7, 1.1 Hz, 1H), 2.46 (s, 3H), 2.25 (d, *J* = 12.5 Hz, 2H), 1.93 – 1.72 (m, 8H). **^{13}C NMR** (100 MHz, $CDCl_3$): δ 186.4, 157.4, 152.6, 120.0, 117.9, 108.5, 39.8, 34.6,

26.0, 24.6, 22.6. **FT-IR** (film) ν_{max}: 2938, 2862, 2237, 1774, 1714, 1679, 1584, 1513, 1452, 1378, 1356, 1293, 1267, 1220, 1106, 1033, 977, 964, 925, 904, 806, 629 cm^{-1}. **HRMS**-(DART) (*m*/*z*) calcd for $C_{13}H_{16}NO_2$ [M+H]$^+$: 218.1181; found: 218.1174.

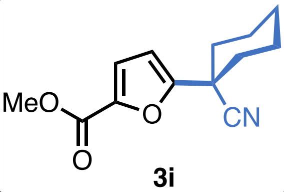

3i

Methyl 5-(1-cyanocyclohexyl)furan-2-carboxylate (3i). Following the general procedure above, using methyl 2-furoate (**2i**) (45 µL, 0.41 mmol), 1,1'-azobis(cyclohexanecarbonitrile) (**1a**) (204 mg, 0.82 mmol), $Cu(OAc)_2$ (76 mg, 0.41 mmol) and 2,2,2-trichloroethanol (TCE) (1.0 mL), the crude reaction mixture was purified by flash column chromatography on silica gel with hexane/acetone (8:2) as eluent to afford the desired compound **3i** (48 mg, 0.206 mmol, 50%) as an orange solid. mp: 70-75 ºC. **^{1}H NMR** (400 MHz, $CDCl_3$): δ 7.12 (d, *J* = 3.5 Hz, 1H), 6.44 (d, *J* = 3.6 Hz, 1H), 3.87 (s, 3H), 2.23 (dd, *J* = 13.1, 1.7 Hz, 2H), 1.94 – 1.71 (m, 8H). **^{13}C NMR** (100 MHz, $CDCl_3$): δ 158.7, 157.3, 144.4, 120.1, 118.6, 108.0, 51.9, 39.8, 34.6, 24.6, 22.6. **FT-IR** (film) ν_{max}: 3128, 2939, 2862, 2237, 1732, 1594, 1530, 1519, 1452, 1437, 1372, 1304, 1268, 1219, 1194, 1138, 1023, 989, 966, 925, 904, 874, 808, 797, 762 cm^{-1}. **HRMS**-(DART) (*m*/*z*) calcd for $C_{13}H_{16}NO_3$ [M+H]$^+$: 234.1130; found: 234.1127.

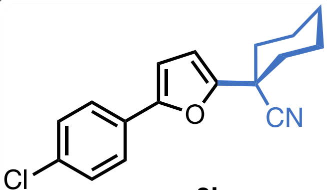

3j

1-(5-(4-Chlorophenyl)furan-2-yl)cyclohexane-1-carbonitrile (3j). Following the general procedure above, using 2-(4-chlorophenyl)furan (**2j**) (87 mg, 0.487 mmol), 1,1'-azobis(cyclohexanecarbonitrile) (**1a**) (243 mg, 0.974 mmol), $Cu(OAc)_2$ (90 mg, 0.487 mmol) and 2,2,2-trichloroethanol (TCE) (1.0 mL), the crude reaction mixture was purified by flash column chromatography on silica gel with hexane/DCM (65:35) as eluent to afford the desired compound **3j** (94 mg, 0.329 mmol, 68%) as a yellow solid. mp: 95-100 ºC. **^{1}H NMR** (400 MHz, $CDCl_3$): δ 7.57 (dt, *J* = 8.7, 2.5 Hz, 2H), 7.35 (dt, *J* = 8.7, 2.4 Hz, 2H), 6.58 (d, *J* = 3.4 Hz, 1H), 6.35 (d, *J* = 3.4 Hz, 1H), 2.32 (dd, *J* = 10.7, 2.2 Hz, 2H), 1.89 – 1.73 (m, 8H). **^{13}C NMR** (100 MHz, $CDCl_3$): δ 153.0, 152.8, 133.3, 128.9, 128.8, 125.0, 120.8, 107.8, 106.0, 39.3, 34.8, 24.9, 22.6. **FT-IR** (film) ν_{max}: 2939, 2860, 2234, 1749, 1668, 1590, 1571, 1539, 1482, 1452, 1407, 1346, 1300, 1283, 1269, 1211, 1155, 1093, 1022, 1011, 965, 920, 904, 874, 829, 786, 732, 653 cm^{-1}. **HRMS**-(DART) (*m*/*z*) calcd for $C_{17}H_{17}ClNO$ [M+H]$^+$: 286.0998; found: 286.0995.

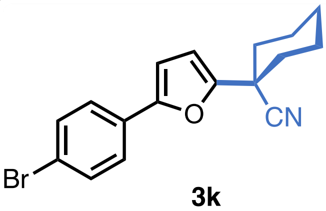

3k

1-(5-(4-Bromophenyl)furan-2-yl)cyclohexane-1-carbonitrile (3k). Following the general procedure above, using 2-(4-bromophenyl)furan (**2k**) (125 mg, 0.56 mmol), 1,1'-azobis(cyclohexanecarbonitrile) (**1a**) (279 mg, 1.121 mmol), $Cu(OAc)_2$ (104 mg, 0.56 mmol) and 2,2,2-trichloroethanol (TCE) (1.4 mL), the crude reaction mixture was purified by flash column chromatography on silica gel with hexane/DCM (65:35) as eluent to afford the desired compound **3k** (156 mg, 0.472 mmol, 84%) as a yellow solid. mp: 98-101 ºC. **^{1}H NMR** (400 MHz, $CDCl_3$): δ 7.50 (s, 4H), 6.59 (d, *J* = 3.4 Hz, 1H), 6.35 (d, *J* = 3.4 Hz, 1H), 2.32 (dd, *J* = 11.0, 2.3 Hz, 2H), 1.89 – 1.71 (m, 8H). **^{13}C NMR** (100 MHz, $CDCl_3$): δ 153.1, 152.8, 131.8, 129.3, 125.2, 121.4, 120.8, 107.9, 106.1, 39.3, 34.8, 24.9, 22.6. **FT-IR** (film) ν_{max}: 2939, 2928, 2907, 2856, 2232, 1747, 1681, 1655, 1636, 1538, 1477, 1452, 1402, 1341, 1268, 1210, 1156, 1071, 1022, 1004, 963, 904, 874, 842, 820, 771, 651 cm^{-1}. **HRMS**-(DART) (*m*/*z*) calcd for $C_{17}H_{17}BrNO$ [M+H]$^+$: 330.0493; found: 330.0486.

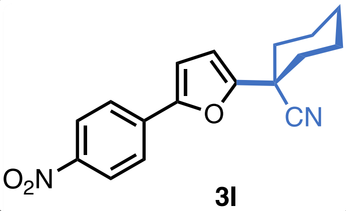

3l

1-(5-(4-Nitrophenyl)furan-2-yl)cyclohexane-1-carbonitrile (3l). Following the general procedure above, using 2-(4-nitrophenyl)furan (**2l**) (75 mg, 0.396 mmol), 1,1'-azobis(cyclohexanecarbonitrile) (**1a**) (198 mg, 0.793 mmol), $Cu(OAc)_2$ (73 mg, 0.396 mmol) and 2,2,2-trichloroethanol (TCE) (1.0 mL), the crude reaction mixture was purified by flash column chromatography on silica gel with hexane/acetone (9:1) as eluent to afford the desired compound **3l** (75 mg, 0.253 mmol, 64%) as a yellow solid. mp: 139-144 ºC. **^{1}H NMR** (400 MHz, $CDCl_3$): δ 8.24 (dt, *J* = 8.9, 2.5, 1.8 Hz, 2H), 7.76 (dt, *J* = 9.1, 2.3, 2.1 Hz, 2H), 6.83 (d, *J* = 3.5 Hz, 1H), 6.43 (d, *J* = 3.5 Hz, 1H), 2.33 (dd, *J* = 11.5, 2.5 Hz, 2H), 1.91 – 1.69 (m, 8H). **^{13}C NMR** (100 MHz, $CDCl_3$): δ 155.0, 151.6, 146.5, 135.9, 124.3, 123.9, 120.5, 109.6, 108.5, 39.4, 34.8, 24.8, 22.6. **FT-IR** (film) ν_{max}: 2939, 2860, 2235, 1633, 1606, 1539, 1515, 1452, 1414, 1337, 1286, 1214, 1180, 1109, 1025, 965, 920, 905, 852, 795, 751, 695 cm^{-1}. **HRMS**-(DART) (*m*/*z*) calcd for $C_{17}H_{17}N_2O_3$ [M+H]$^+$: 297.1239; found: 297.1231.

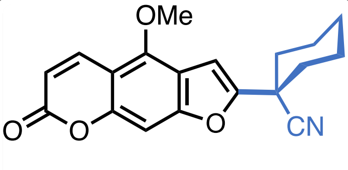

3m

1-(4-Methoxy-7-oxo-7*H*-furo[3,2-*g*]chromen-2-yl)cyclohexane-1-carbonitrile (3m). Following the general procedure above, using 5-methoxypsoralen (**2m**) (45 mg, 0.205 mmol), 1,1'-azobis(cyclohexanecarbonitrile) (**1a**) (102 mg, 0.41 mmol), $Cu(OAc)_2$ (38 mg, 0.205 mmol) and 2,2,2-trichloroethanol (TCE) (0.5 mL), the crude reaction mixture was purified by flash column chromatography on silica gel with DCM/hexane (9:1) as eluent to afford the desired compound **3m** (36 mg, 0.112 mmol, 55%) as a white solid. mp: 245-250 ºC. **^{1}H NMR** (400 MHz, $CDCl_3$): δ 8.14 (dd, *J* = 9.8, 0.7 Hz, 1H), 7.10 (t, *J* = 0.8 Hz, 1H), 6.94 (d, *J* = 0.9 Hz, 1H), 6.28 (d, *J* = 9.8 Hz, 1H), 4.26 (s, 3H), 2.29 (d, *J* = 14.3 Hz, 2H), 2.01 – 1.77 (m, 8H). **^{13}C NMR** (100 MHz, $CDCl_3$): δ 161.0, 158.2, 156.2, 152.9, 149.5, 139.1, 120.2, 112.9, 112.8, 106.7, 101.0, 93.8, 60.2, 40.0, 34.6, 24.7, 22.7. **FT-IR** (KBr) ν_{max}: 3127, 2941, 2859, 2234, 1721, 1626, 1595, 1474, 1459, 1431, 1380, 1356, 1337, 1276, 1222, 1129, 1108, 1024, 988, 892, 826, 809, 754, 720, 678, 606, 543, 464, 424 cm^{-1}. **HRMS**-(DART) (*m*/*z*) calcd for $C_{19}H_{18}NO_4$ [M+H]$^+$: 324.1235; found: 324.1244.

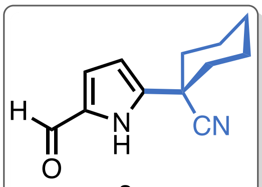

3n

1-(5-Formyl-1*H*-pyrrol-2-yl)cyclohexane-1-carbonitrile (3n). Following the general procedure above, using pyrrole-2-carboxaldehyde (**2n**) (40 mg, 0.41 mmol), 1,1'-azobis(cyclohexanecarbonitrile) (**1a**) (204 mg, 0.82 mmol), $Cu(OAc)_2$ (76 mg, 0.41 mmol) and 2,2,2-trichloroethanol (TCE) (1.0 mL), the crude reaction mixture was purified by flash column chromatography on silica gel with hexane/EtOAc (95:5) as eluent to afford the desired compound **3n** (28 mg, 0.138 mmol, 34%) as yellow crystals. mp: 100-105 ºC. ^{1}**H NMR** (500 MHz, $CDCl_3$): δ 9.89 (bs, 1H), 9.48 (s, 1H), 6.93 (dd, *J* = 4.0, 2.4 Hz, 1H), 6.32 (dd, *J* = 3.9, 2.6 Hz, 1H), 2.27 (d, *J* = 10.3 Hz, 2H), 1.90 – 1.75 (m, 8H). ^{13}**C NMR** (125 MHz, $CDCl_3$): δ 179.2, 140.2, 132.9, 121.6, 120.8, 108.5, 39.2, 36.1, 24.7, 23.0. **FT-IR** (ATR) ν_{max}: 3255, 2925, 2854, 2236, 1722, 1646, 1477, 1446, 1416, 1339, 1282, 1258, 1196, 1117, 1047, 1001, 968, 938, 904, 872, 827, 799, 769, 759, 613, 522, 472 cm^{-1}. **HRMS**-(DART) (*m/z*) calcd for $C_{12}H_{15}N_2O$ [M+H]$^+$: 203.1184; found: 203.1184.

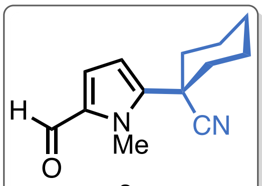

3o

1-(5-Formyl-1-methyl-1*H*-pyrrol-2-yl)cyclohexane-1-carbonitrile (3o). Following the general procedure above, using *N*-methyl-2-pyrrolecarboxaldehyde (**2o**) (46 mg, 0.41 mmol), 1,1'-azobis(cyclohexanecarbonitrile) (**1a**) (204 mg, 0.82 mmol), $Cu(OAc)_2$ (76 mg, 0.41 mmol) and 2,2,2-trichloroethanol (TCE) (1.0 mL), the crude reaction mixture was purified by flash column chromatography on silica gel with DCM/hexane (8:2 to 100:0) as eluent to afford the desired compound **3o** (21 mg, 0.097 mmol, 24%) as a colorless oil. ^{1}**H NMR** (500 MHz, $CDCl_3$): δ 9.54 (s, 1H), 6.87 (d, *J* = 4.2 Hz, 1H), 6.16 (d, *J* = 4.2 Hz, 1H), 4.17 (s, 3H), 2.39 (dd, *J* = 13.2, 1.6 Hz, 2H), 1.93 – 1.77 (m, 6H), 1.71 (td, *J* = 13.2, 5.2 Hz, 2H). ^{13}**C NMR** (125 MHz, $CDCl_3$): δ 180.0, 140.9, 133.7, 123.8, 120.1, 107.9, 37.5, 35.5, 34.4, 24.8, 22.7. **FT-IR** (ATR) ν_{max}: 2936, 2860, 2233, 1660, 1529, 1464, 1382, 1346, 1324, 1260, 1211, 1186, 1142, 1048, 1022, 950, 905, 875, 835, 770, 685, 628, 520, 444 cm^{-1}. **HRMS**-(DART) (*m/z*) calcd for $C_{13}H_{17}N_2O$ [M+H]$^+$: 217.1340; found: 217.1337.

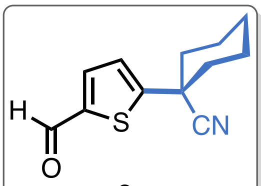

3p

1-(5-formylthiophen-2-yl)cyclohexane-1-carbonitrile (3p). Following the general procedure above, using 2-thiophenecarboxaldehyde (**2p**) (38 µL, 0.41 mmol), 1,1'-azobis(cyclohexanecarbonitrile) (**1a**) (204 mg, 0.82 mmol), $Cu(OAc)_2$ (76 mg, 0.41 mmol) and 2,2,2-trichloroethanol (TCE) (1.0 mL), the crude reaction mixture was purified by flash column chromatography on silica gel with hexane/acetone (99:1) as eluent to afford the desired compound **3p** (20 mg, 0.091 mmol, 22%) as a pale-yellow solid. mp: 48-50 ºC. ^{1}**H NMR** (500 MHz, $CDCl_3$): δ 9.88 (s, 1H), 7.67 (d, *J* = 3.9 Hz, 1H), 7.29 (d, *J* = 3.9 Hz, 1H), 2.35 – 2.29 (m, 2H), 1.92 – 1.77 (m, 8H). ^{13}**C NMR** (125 MHz, $CDCl_3$): δ 182.8, 156.2, 142.8, 136.1, 125.7, 120.9, 41.8, 38.8, 24.6, 23.3. **FT-IR** (ATR) ν_{max}: 2935, 2859, 2813, 2748, 2235, 1671, 1530, 1452, 1354, 1317, 1262, 1214, 1192, 1053, 996, 936, 906, 876, 811, 756, 668, 600, 528, 428 cm^{-1}. **HRMS**-(DART) (*m/z*) calcd for $C_{12}H_{14}NOS$ [M+H]$^+$: 220.0796; found: 220.0795.

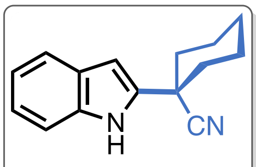

3q

1-(1*H*-indol-2-yl)cyclohexane-1-carbonitrile (3q). Following the general procedure above, using indole (**2q**) (48 mg, 0.41 mmol), 1,1'-azobis(cyclohexanecarbonitrile) (**1a**) (204 mg, 0.82 mmol), $Cu(OAc)_2$ (76 mg, 0.41 mmol) and 2,2,2-trichloroethanol (TCE) (1.0 mL), the crude reaction mixture was purified by flash column chromatography on silica gel with hexane/acetone (95:5) as eluent to afford the desired compound **3q** (16 mg, 0.071 mmol, 17%) as brown crystals. mp: 137-139 ºC. ^{1}**H NMR** (400 MHz, $CDCl_3$): δ 8.37 (bs, 1H), 7.59 (d, *J* = 7.8 Hz, 1H), 7.38 (d, *J* = 8.1 Hz, 1H), 7.21 (td, *J* = 7.2, 0.9 Hz, 1H), 7.13 (td, *J* = 7.8, 0.6 Hz, 1H), 6.45 (d, *J* = 2.2 Hz, 1H), 2.42 – 2.30 (m, 2H), 1.96 – 1.75 (m, 7H). ^{13}**C NMR** (100 MHz, $CDCl_3$): δ 137.9, 135.9, 127.8, 122.5, 121.7, 120.6, 120.3, 111.0, 99.3, 39.3, 36.4, 24.9, 23.1. **FT-IR** (ATR) ν_{max}: 3346, 3300, 3056, 2935, 2855, 2240, 1920, 1887, 1854, 1804, 1715, 1618, 1584, 1533, 1493, 1451, 1423, 1340, 1291, 1261, 1243, 1223, 1166, 1137, 1113, 1039, 1003, 969, 933, 903, 880, 840, 790, 749, 733, 682, 652, 625, 599, 584, 564, 483, 455, 428 cm^{-1}. **HRMS**-(DART) (*m/z*) calcd for $C_{15}H_{17}N_2$ [M+H]$^+$: 225.1391; found: 225.1392.

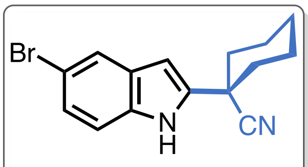

3r

1-(5-bromo-1*H*-indol-2-yl)cyclohexane-1-carbonitrile (3r). Following the general procedure above, using 5-bromoindole (**2r**) (80 mg, 0.406 mmol), 1,1'-azobis(cyclohexanecarbonitrile) (**1a**) (202 mg, 0.812 mmol), $Cu(OAc)_2$ (75 mg, 0.406 mmol) and 2,2,2-trichloroethanol (TCE) (1.0 mL), the crude reaction mixture was purified by flash column chromatography on silica gel with hexane/acetone (95:5) as eluent to afford the desired compound **3r** (20 mg, 0.066 mmol, 16%) as a white solid. mp: 140-144 ºC. ^{1}**H NMR** (400 MHz, $CDCl_3$): δ 8.42 (bs, 1H), 7.69 (d, *J* = 1.8 Hz, 1H), 7.30 – 7.21 (m, 2H), 6.37 (dd, *J* = 2.2, 0.7 Hz, 1H), 2.39 – 2.30 (m, 2H), 1.94 – 1.73 (m, 8H). ^{13}**C NMR** (100 MHz, $CDCl_3$): δ 139.2, 134.5, 129.6, 125.4, 123.1, 121.5, 113.4, 112.4, 98.9, 39.3, 36.4, 24.9, 23.1. **FT-IR** (ATR) ν_{max}: 2943, 2930, 2856, 2240, 1725, 1577, 1467, 1447, 1412, 1342, 1309, 1263, 1235, 1217, 1168, 1046, 973, 933, 903, 855, 795, 764, 731, 686, 668, 583, 558, 521, 498, 453, 429 cm^{-1}. **HRMS**-(DART) (*m/z*) calcd for $C_{15}H_{16}BrN_2$ [M+H]$^+$: 303.0496; found: 303.0490.

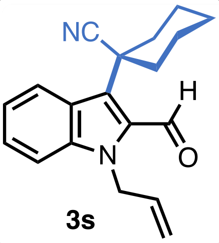

3s

1-(1-Allyl-2-formyl-1*H*-indol-3-yl)cyclohexane-1-carbonitrile (3s). Following the general procedure above, using 1-allyl-1*H*-indole-2-carbaldehyde (**2s**) (76 mg, 0.41 mmol), 1,1'-azobis(cyclohexanecarbonitrile) (**1a**) (204 mg, 0.82 mmol), $Cu(OAc)_2$ (76 mg, 0.41 mmol) and 2,2,2-trichloroethanol (TCE) (1.0 mL), the crude reaction mixture was purified by flash column chromatography on silica gel with hexane/acetone (9:1) as eluent to afford the desired compound **3s** (33 mg, 0.113 mmol, 28%) as a brown oil. ^{1}**H NMR** (400 MHz, $CDCl_3$): δ 10.72 (s, 1H), 8.11 (d, *J* = 8.5 Hz, 1H), 7.45 – 7.35 (m, 2H), 7.19 (ddd, *J* = 8.2, 5.3, 2.7 Hz, 1H), 5.98 (ddt, *J* = 17.2, 10.2, 5.0 Hz, 1H), 5.20 (dt, *J* = 5.1, 1.7 Hz, 2H), 5.13 (dq, *J* = 10.3, 1.4 Hz, 1H), 4.92 (dq, *J* = 17.0, 1.6 Hz, 1H), 2.55 (dd, *J* = 13.5, 1.5 Hz, 2H), 2.25 (td, *J* = 13.1, 5.0 Hz, 2H), 2.05 – 1.83 (m, 6H). ^{13}**C NMR** (100 MHz, $CDCl_3$): δ 183.0, 138.8, 133.3, 130.6,

126.8, 126.4, 124.0, 123.2, 122.7, 121.3, 116.5, 111.2, 47.4, 40.4, 38.9, 24.9, 23.4. **FT-IR** (film) ν_{max}: 2935, 2861, 2230, 1718, 1662, 1610, 1502, 1485, 1461, 1410, 1395, 1370, 1358, 1328, 1256, 1224, 1192, 1158, 1064, 1026, 994, 924, 862, 745, 634 cm^{-1}. **HRMS**-(DART) (*m*/*z*) calcd for $C_{19}H_{21}N_2O$ [M+H]$^+$: 293.1653; found: 293.1645.

1.3 Procedure for the synthesis of α-heteroaryl nitriles 4

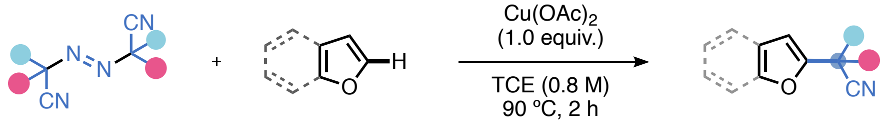

1b–e (1.0 equiv.) **2** (3.0 equiv.) **4**

b,**c** R^1= R^2= Me, Et

d,**e** R^1= Me R^2= $(CH_2)_2COOH$, $(CH_2)_2COOMe$

In a microwave reaction vial equipped with a stir bar was charged with azobis(alkylcarbonitrile) **1b**–**e** (1.0 equiv.), heteroarene **2** (3.0 equiv.), $Cu(OAc)_2$ (1.0 equiv.) and 2,2,2-trichloroethanol (TCE) (0.8 M). The vial was sealed with a PTFE lined butyl rubber septum and aluminum crimp cap, and the solution was degassed by three consecutive freeze−pump−thaw cycles using liquid nitrogen and backfilled with pure argon. The mixture was stirred at 90 ºC in heating mantle for 2 h. After cooling to room temperature, the crude reaction mixture was extracted with a saturated solution of $NaHCO_3$ and DCM, unless the extraction procedure states otherwise. The organic phase was dried over anhydrous Na_2SO_4 and evaporated under reduced pressure. The residue was purified by flash column chromatography on silica gel to afford the desired α-heteroaryl nitrile **4**.

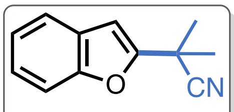

4a

2-(Benzofuran-2-yl)-2-methylpropanenitrile (4a). Following the general procedure above, using 2,3-benzofuran (**2a**) (264 μL, 2.375 mmol), 2,2'-azobis(2-methylpropionitrile) (**1b**) (130 mg, 0.792 mmol), $Cu(OAc)_2$ (147 mg, 0.792 mmol) and 2,2,2-trichloroethanol (TCE) (1.5 mL), the crude reaction mixture was purified by flash column chromatography on silica gel with hexane/DCM (65:35) as eluent to afford the desired compound **4a** (54 mg, 0.292 mmol, 37%) as a yellow oil. ^{1}**H NMR** (400 MHz, $CDCl_3$): δ 7.55 (dd, *J* = 7.2, 1.1 Hz, 1H), 7.48 (dd, *J* = 8.2, 1.0 Hz, 1H), 7.31 (td, *J* = 7.3, 1.4 Hz, 1H), 7.24 (td, *J* = 7.4, 0.9 Hz, 1H), 6.69 (d, *J* = 0.9 Hz, 1H), 1.82 (s, 6H). ^{13}**C NMR** (100 MHz, $CDCl_3$): δ 155.9, 155.0, 127.7, 124.7, 123.2, 122.1, 121.2, 111.3, 102.5, 33.1, 26.3. **FT-IR** (ATR) ν_{max}: 2988, 2918, 2849, 2238, 1624, 1599, 1584, 1543, 1453, 1368, 1304, 1278, 1252, 1216, 1196, 1169, 1145, 1110, 1089, 1008, 973, 944, 881, 809, 749, 688, 612, 577, 527, 497, 446, 416 cm^{-1}. **HRMS**-(DART) (*m*/*z*) calcd for $C_{12}H_{12}NO$ [M+H]$^+$: 186.0918; found: 186.0910.

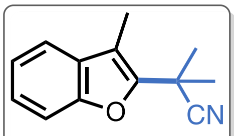

4b

2-Methyl-2-(3-methylbenzofuran-2-yl)propanenitrile (4b). Following the general procedure above, using 3-methylindole (**2e**) (328 mg, 2.46 mmol), 2,2'-azobis(2-methylpropionitrile) (**1b**) (135 mg, 0.82 mmol), $Cu(OAc)_2$ (152 mg, 0.82 mmol) and 2,2,2-trichloroethanol (TCE) (1.0 mL), the crude reaction mixture was purified by flash column chromatography on silica gel with hexane/DCM (65:35) as eluent to afford the desired compound **4b** (78 mg, 0.391 mmol, 48%) as a yellow oil. ^{1}**H NMR** (400 MHz, $CDCl_3$): δ 7.50 (ddd, *J* = 7.3, 1.6, 0.7 Hz, 1H), 7.41 (ddd, *J* = 7.7, 1.5, 0.6 Hz, 1H), 7.30 (td, *J* = 7.2, 1.5 Hz, 1H), 7.25 (td, *J* = 7.4, 1.3 Hz, 1H), 2.43 (s, 3H), 1.84 (s, 6H). ^{13}**C NMR** (100 MHz, $CDCl_3$): δ 153.0, 148.5, 130.3, 124.6, 122.6, 122.4, 119.3, 111.2, 110.9, 32.7, 26.9, 8.3. **FT-IR** (film) ν_{max}: 3064, 3040, 2988, 2936, 2873, 2240, 1766, 1647, 1608, 1455, 1391, 1368, 1285, 1261, 1216, 1200, 1153, 1127, 1108, 1094, 1007, 929, 875, 857, 747, 711, 683 cm^{-1}. **HRMS**-(DART) (*m*/*z*) calcd for $C_{13}H_{14}NO$ [M+H]$^+$: 200.1075; found: 200.1067.

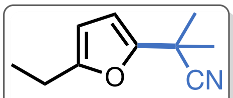

4c

2-(5-Ethylfuran-2-yl)-2-methylpropanenitrile (4c). Following the general procedure above, using 2-ethylfuran (**2f**) (239 mg, 2.46 mmol), 2,2'-azobis(2-methylpropionitrile) (**1b**) (135 mg, 0.82 mmol), $Cu(OAc)_2$ (152 mg, 0.82 mmol) and 2,2,2-trichloroethanol (TCE) (1.0 mL), the crude reaction mixture was purified by flash column chromatography on silica gel with hexane/DCM (8:2) as eluent to afford the desired compound **4c** (74 mg, 0.453 mmol, 55%) as a colorless oil (volatile compound). ^{1}**H NMR** (400 MHz, $CDCl_3$): δ 6.12 (d, *J* = 3.2 Hz, 1H), 5.91 (dt, *J* = 3.2, 1.1 Hz, 1H), 2.63 (qd, *J* = 7.5, 1.1 Hz, 2H), 1.68 (s, 6H), 1.22 (t, *J* = 7.6 Hz, 3H). ^{13}**C NMR** (100 MHz, $CDCl_3$): δ 158.2, 151.0, 122.7, 106.0, 104.6, 32.5, 26.3, 21.3, 11.9. **FT-IR** (ATR) ν_{max}: 2978, 2940, 2878, 2237, 1763, 1606, 1557, 1460, 1388, 1366, 1323, 1260, 1205, 1127, 1055, 1014, 981, 957, 936, 793, 780, 720, 691, 601, 564, 487, 472, 439 cm^{-1}. **HRMS**-(DART) (*m*/*z*) calcd for $C_{10}H_{14}NO$ [M+H]$^+$: 164.1075; found: 164.1076.

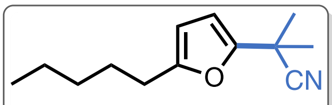

4d

2-Methyl-2-(5-pentylfuran-2-yl)propanenitrile (4d). Following the general procedure above, using 2-pentylfuran (**2g**) (343 mg, 2.46 mmol), 2,2'-azobis(2-methylpropionitrile) (**1b**) (135 mg, 0.82 mmol), $Cu(OAc)_2$ (152 mg, 0.82 mmol) and 2,2,2-trichloroethanol (TCE) (1.0 mL), the crude reaction mixture was purified by flash column chromatography on silica gel with hexane/DCM (8:2) as eluent to afford the desired compound **4d** (74 mg, 0.360 mmol, 44%) as an orange oil. ^{1}**H NMR** (400 MHz, $CDCl_3$): δ 6.11 (d, *J* = 3.2 Hz, 1H), 5.90 (dt, *J* = 3.2, 1.0 Hz, 1H), 2.59 (t, *J* = 7.6 Hz, 2H), 1.68 (s, 6H), 1.67 – 1.58 (m, 2H), 1.38 – 1.28 (m, 4H), 0.90 (t, *J* = 7.0 Hz, 3H). ^{13}**C NMR** (100 MHz, $CDCl_3$): δ 157.1, 151.0, 122.7, 105.9, 105.3, 32.5, 31.3, 27.9, 27.5, 26.3, 22.3, 14.0. **FT-IR** (film) ν_{max}: 2987, 2957, 2933, 2861, 2238, 1804, 1760, 1713, 1647, 1607, 1557, 1466, 1388, 1367, 1261, 1220, 1194, 1174, 1128, 1016, 966, 950, 786, 732, 690 cm^{-1}. **HRMS**-(DART) (*m*/*z*) calcd for $C_{13}H_{20}NO$ [M+H]$^+$: 206.1544; found: 206.1535.

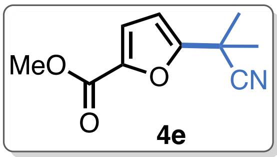

4e

Methyl 5-(2-cyanopropan-2-yl)furan-2-carboxylate (4e). Following the general procedure above, using methyl 2-furoate (**2i**) (269 μL, 2.46 mmol), 2,2'-azobis(2-methylpropionitrile) (**1b**) (135 mg, 0.82 mmol), $Cu(OAc)_2$ (152 mg, 0.82 mmol) and 2,2,2-trichloroethanol (TCE) (1.0 mL), the crude reaction mixture was purified by flash column chromatography on silica gel with hexane/EtOAc (9:1) as eluent to afford the desired compound **4e** (35 mg, 0.181 mmol, 22%) as an orange oil. **^{1}H NMR** (500 MHz, $CDCl_3$): δ 7.11 (d, *J* = 3.5 Hz, 1H), 6.43 (d, *J* = 3.5 Hz, 1H), 3.88 (s, 3H), 1.76 (s, 6H). **^{13}C NMR** (125 MHz, $CDCl_3$): δ 158.7, 157.0, 144.7, 121.6, 118.6, 107.9, 52.0, 33.1, 26.3. **FT-IR** (ATR) ν_{max}: 3129, 2991, 2952, 2849, 2239, 1728, 1549, 1520, 1459, 1436, 1391, 1367, 1300, 1252, 1221, 1196, 1137, 1123, 1022, 987, 961, 925, 808, 796, 761, 722, 609, 513, 417 cm^{-1}. **HRMS**-(DART) (*m*/*z*) calcd for $C_{10}H_{12}NO_3$ [M+H]$^+$: 194.0817; found: 194.0816.

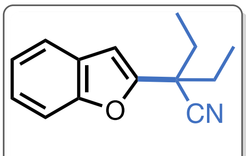

4f

2-(Benzofuran-2-yl)-2-ethylbutanenitrile (4f). Following the general procedure above, using 2,3-benzofuran (**2a**) (274 μL, 2.46 mmol), 2,2'-azobis(2-ethylbutanenitrile) (**1c**) (184 mg, 0.82 mmol), $Cu(OAc)_2$ (152 mg, 0.82 mmol) and 2,2,2-trichloroethanol (TCE) (1.0 mL), the crude reaction mixture was purified by flash column chromatography on silica gel with hexane/DCM (9:1) as eluent to afford the desired compound **4f** (27 mg, 0.127 mmol, 15%) as a colorless oil. **^{1}H NMR** (500 MHz, $CDCl_3$): δ 7.56 (dd, *J* = 7.6, 1.4 Hz, 1H), 7.45 (d, *J* = 8.1 Hz, 1H), 7.29 (td, *J* = 8.0, 7.4, 1.5 Hz, 1H), 7.25 (td, *J* = 7.4, 1.0 Hz, 1H), 6.83 (s, 1H), 2.17 – 2.00 (m, 4H), 1.01 (t, *J* = 7.4 Hz, 6H). **^{13}C NMR** (125 MHz, $CDCl_3$): δ 155.1, 153.5, 127.7, 124.4, 123.1, 121.0, 120.5, 111.2, 105.6, 45.9, 31.0, 9.7. **FT-IR** (ATR) ν_{max}: 2972, 2936, 2880, 2240, 1664, 1645, 1616, 1598, 1580, 1545, 1453, 1384, 1351, 1304, 1246, 1190, 1166, 1147, 1110, 1092, 1032, 1009, 955, 930, 871, 808, 749, 685, 603, 578, 489, 448, 415 cm^{-1}. **HRMS**-(DART) (*m*/*z*) calcd for $C_{14}H_{16}NO$ [M+H]$^+$: 214.1231; found: 214.1224.

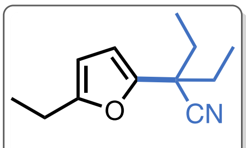

4g

2-Ethyl-2-(5-ethylfuran-2-yl)butanenitrile (4g). Following the general procedure above, using 2-ethylfuran (**2f**) (262 μL, 2.46 mmol), 2,2'-azobis(2-ethylbutanenitrile) (**1c**) (181 mg, 0.82 mmol), $Cu(OAc)_2$ (152 mg, 0.82 mmol) and 2,2,2-trichloroethanol (TCE) (1.0 mL), the crude reaction mixture was purified by flash column chromatography on silica gel with hexane/DCM (9:1) as eluent to afford the desired compound **4g** (45 mg, 0.235 mmol, 29%) as a yellow oil (volatile compound). **^{1}H NMR** (500 MHz, $CDCl_3$): δ 6.23 (d, *J* = 3.1 Hz, 1H), 5.90 (dt, *J* = 3.2, 1.1 Hz, 1H), 2.60 (qd, *J* = 7.6, 1.1 Hz, 2H), 2.00 – 1.87 (m, 4H), 1.20 (t, *J* = 7.6 Hz, 3H), 0.95 (t, *J* = 7.4 Hz, 6H). **^{13}C NMR** (125 MHz, $CDCl_3$): δ 158.0, 148.5, 121.1, 108.6, 104.3, 45.0, 30.9, 21.3, 12.0, 9.5. **FT-IR** (ATR) ν_{max}: 2972, 2937, 2880, 2239, 1752, 1606, 1557, 1469, 1383, 1324, 1264, 1208, 1178, 1125, 1056, 1013, 982, 965, 927, 868, 788, 719, 572, 429 cm^{-1}. **HRMS**-(DART) (*m*/*z*) calcd for $C_{12}H_{18}NO$ [M+H]$^+$: 192.1388; found: 192.1390.

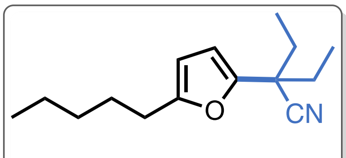

4h

2-Ethyl-2-(5-pentylfuran-2-yl)butanenitrile (4h). Following the general procedure above, using 2-pentylfuran (**2g**) (387 μL, 2.46 mmol), 2,2'-azobis(2-ethylbutanenitrile) (**1c**) (181 mg, 0.82 mmol), $Cu(OAc)_2$ (152 mg, 0.82 mmol) and 2,2,2-trichloroethanol (TCE) (1.0 mL), the crude reaction mixture was purified by flash column chromatography on silica gel with hexane/DCM (9:1) as eluent to afford the desired compound **4h** (114 mg, 0.489 mmol, 60%) as a yellowish oil. **^{1}H NMR** (500 MHz, $CDCl_3$): δ 6.23 (d, *J* = 3.1 Hz, 1H), 5.90 (dt, *J* = 3.2, 0.9 Hz, 1H), 2.57 (t, *J* = 7.6 Hz, 2H), 1.99 – 1.87 (m, 4H), 1.60 (p, *J* = 7.5 Hz, 2H), 1.37 – 1.26 (m, 4H), 0.95 (t, *J* = 7.4 Hz, 6H), 0.89 (t, *J* = 7.0 Hz, 3H). **^{13}C NMR** (125 MHz, $CDCl_3$): δ 156.8, 148.5, 121.1, 108.7, 105.1, 45.0, 31.2, 31.0, 27.9, 27.6, 22.3, 13.9, 9.5. **FT-IR** (ATR) ν_{max}: 2969, 2932, 2860, 2875, 2860, 2239, 1752, 1606, 1556, 1459, 1382, 1342, 1305, 1270, 1219, 1171, 1126, 1015, 968, 934, 867, 784, 720, 572, 433 cm^{-1}. **HRMS**-(DART) (*m*/*z*) calcd for $C_{15}H_{24}NO$ [M+H]$^+$: 234.1857; found: 234.1853.

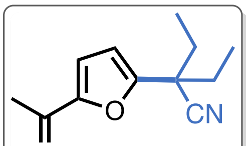

4i

2-(5-Acetylfuran-2-yl)-2-ethylbutanenitrile (4i). Following the general procedure above, using 2-furyl methyl ketone (**2h**) (192 μL, 1.879 mmol), 2,2'-azobis(2-ethylbutanenitrile) (**1c**) (138 mg, 0.626 mmol), $Cu(OAc)_2$ (116 mg, 0.626 mmol) and 2,2,2-trichloroethanol (TCE) (0.76 mL), the crude reaction mixture was purified by flash column chromatography on silica gel with hexane/acetone (95:5) as eluent to afford the desired compound **4i** (30 mg, 0.146 mmol, 23%) as a colorless oil. **^{1}H NMR** (700 MHz, $CDCl_3$): δ 7.13 (d, *J* = 3.5 Hz, 1H), 6.57 (d, *J* = 3.5 Hz, 1H), 2.45 (s, 3H), 2.10 – 1.98 (m, 4H), 0.98 (t, *J* = 7.4 Hz, 6H). **^{13}C NMR** (175 MHz, $CDCl_3$): δ 186.1, 155.6, 152.9, 120.0, 118.0, 111.1, 45.9, 31.2, 25.9, 9.7. **FT-IR** (ATR) ν_{max}: 2974, 2938, 2881, 2239, 1712, 1679, 1583, 1510, 1459, 1382, 1356, 1291, 1279, 1215, 1102, 1084, 1029, 979, 964, 920, 867, 804, 718, 631, 567, 546, 484, 433 cm^{-1}. **HRMS**-(DART) (*m*/*z*) calcd for $C_{12}H_{16}NO_2$ [M+H]$^+$: 206.1181; found: 206.1183.

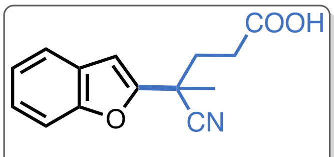

4j

4-(Benzofuran-2-yl)-4-cyanopentanoic acid (4j). Following the general procedure above, using 2,3-benzofuran (**2a**) (274 μL, 2.46 mmol), 4,4'-azobis(4-cyanovaleric acid) (**1d**) (235 mg, 0.82 mmol), $Cu(OAc)_2$ (152 mg, 0.82 mmol) and 2,2,2-trichloroethanol (TCE) (1.5 mL), the crude reaction mixture was extracted with EtOAc. The organic phase was washed with water, then brine, dried over anhydrous Na_2SO_4 and evaporated in vacuo. The residue was purified by flash column chromatography on silica gel with hexane/EtOAc (7:3 + 1% formic acid) as eluent to afford the desired compound **4j** (35 mg, 0.144 mmol, 18%) as a brown oil. **^{1}H NMR** (400 MHz, $CDCl_3$): δ 7.55 (ddd, *J* = 7.6, 1.5, 0.7 Hz, 1H), 7.46 (dd, *J* = 8.2, 0.9 Hz, 1H), 7.30 (td, *J* = 7.3, 1.3 Hz, 1H), 7.24 (td, *J* = 7.3, 1.0 Hz, 1H), 6.79 (d, *J* = 0.9 Hz, 1H), 2.64 – 2.54 (m, 1H), 2.49 – 2.27 (m, 3H), 1.83 (s, 3H). **^{13}C NMR** (100 MHz, $CDCl_3$): δ 177.1, 155.1, 153.5, 127.4, 124.9, 123.3, 121.3, 120.6, 111.4, 104.4, 37.7, 33.8, 30.0, 24.8. **FT-IR** (ATR) ν_{max}: 3201, 2923, 2852, 2240, 1710, 1664, 1580, 1452, 1414, 1297, 1251, 1206, 1167, 1142, 1075, 1037, 1008, 938, 881, 852, 811, 750, 682, 612, 577, 496, 446 cm^{-1}. **HRMS**-(DART) (*m*/*z*) calcd for $C_{14}H_{14}NO_3$ [M+H]$^+$: 244.0973; found: 244.0969.

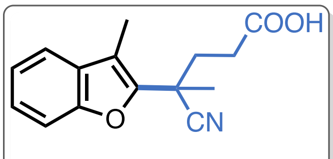

4k

4-Cyano-4-(3-methylbenzofuran-2-yl)pentanoic acid (4k). Following the general procedure above, using 3-methylindole (**2e**) (314 µL, 2.46 mmol), 4,4'-azobis(4-cyanovaleric acid) (**1d**) (235 mg, 0.82 mmol), $Cu(OAc)_2$ (152 mg, 0.82 mmol) and 2,2,2-trichloroethanol (TCE) (1.0 mL), the crude reaction mixture was extracted with EtOAc. The organic phase was washed with water, then brine, dried over anhydrous Na_2SO_4 and evaporated in vacuo. The residue was purified by flash column chromatography on silica gel with hexane/EtOAc (7:3 + 1% formic acid) as eluent to afford the desired compound **4k** (72 mg, 0.280 mmol, 34%) as a yellow solid. mp: 62-67 ºC. **^{1}H NMR** (400 MHz, $CDCl_3$): δ 7.49 (ddd, J = 7.6, 1.6, 0.7 Hz, 1H), 7.40 (ddd, J = 7.9, 1.1, 0.7 Hz, 1H), 7.30 (td, J = 7.3, 1.4 Hz, 1H), 7.24 (td, J = 7.6, 1.0 Hz, 1H), 2.63 – 2.46 (m, 2H), 2.45 (s, 3H), 2.44 – 2.26 (m, 2H), 1.86 (s, 3H). **^{13}C NMR** (100 MHz, $CDCl_3$): δ 177.6, 153.0, 146.0, 130.0, 124.9, 122.7, 120.8, 119.5, 112.9, 111.0, 36.9, 34.7, 30.2, 25.7, 8.3. **FT-IR** (film) ν_{max}: 3394, 3042, 2987, 2933, 2242, 1711, 1455, 1418, 1391, 1298, 1275, 1259, 1218, 1151, 1097, 1007, 932, 873, 811, 748, 712, 635 cm^{-1}. **HRMS**-(DART) (m/z) calcd for $C_{15}H_{16}NO_3$ [M+H]$^+$: 258.1130; found: 258.1134.

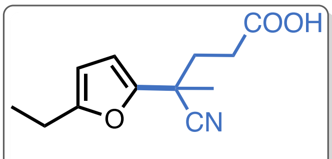

4l

4-Cyano-4-(5-ethylfuran-2-yl)pentanoic acid (4l). Following the general procedure above, using 2-ethylfuran (**2f**) (262 µL, 2.46 mmol), 4,4'-azobis(4-cyanovaleric acid) (**1d**) (235 mg, 0.82 mmol), $Cu(OAc)_2$ (152 mg, 0.82 mmol) and 2,2,2-trichloroethanol (TCE) (1.0 mL), the crude reaction mixture was extracted with EtOAc. The organic phase was washed with water, then brine, dried over anhydrous Na_2SO_4 and evaporated in vacuo. The residue was purified by flash column chromatography on silica gel with hexane/EtOAc (7:3 + 1% formic acid) as eluent to afford the desired compound **4l** (97 mg, 0.438 mmol, 54%) as a brown oil. **^{1}H NMR** (400 MHz, $CDCl_3$): δ 6.21 (d, J = 3.2 Hz, 1H), 5.91 (dt, J = 3.1, 1.1 Hz, 1H), 2.61 (qd, J = 7.6, 1.1 Hz, 2H), 2.55 (ddd, J = 16.6, 10.5, 5.8 Hz, 1H), 2.42 (ddd, J = 16.8, 10.6, 5.4 Hz, 1H), 2.34 – 2.17 (m, 2H), 1.70 (s, 3H), 1.21 (t, J = 7.5 Hz, 3H). **^{13}C NMR** (100 MHz, $CDCl_3$): δ 177.6, 158.6, 148.7, 121.1, 107.7, 104.7, 37.0, 34.0, 30.0, 24.6, 21.3, 11.9. **FT-IR** (ATR) ν_{max}: 3206, 2977, 2939, 2241, 1709, 1556, 1514, 1453, 1415, 1382, 1291, 1202, 1176, 1111, 1017, 980, 953, 932, 797, 720, 621, 571, 486 cm^{-1}. **HRMS**-(DART) (m/z) calcd for $C_{12}H_{16}NO_3$ [M+H]$^+$: 222.1130; found: 222.1123.

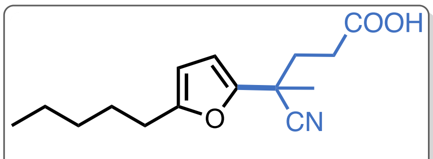

4m

4-Cyano-4-(5-pentylfuran-2-yl)pentanoic acid (4m). Following the general procedure above, using 2-pentylfuran (**2g**) (392 µL, 2.46 mmol), 4,4'-azobis(4-cyanovaleric acid) (**1d**) (235 mg, 0.82 mmol), $Cu(OAc)_2$ (152 mg, 0.82 mmol) and 2,2,2-trichloroethanol (TCE) (1.0 mL), the crude reaction mixture was extracted with EtOAc. The organic phase was washed with water, then brine, dried over anhydrous Na_2SO_4 and evaporated in vacuo. The residue was purified by flash column chromatography on silica gel with hexane/EtOAc (7:3 + 1% formic acid) as eluent to afford the desired compound **4m** (98 mg, 0.372 mmol, 45%) as a brown oil. **^{1}H NMR** (400 MHz, $CDCl_3$): δ 6.21 (d, J = 3.2 Hz, 1H), 5.91 (dt, J = 3.2, 1.0 Hz, 1H), 2.61 – 2.49 (m, 3H), 2.40 (ddd, J = 16.6, 10.7, 5.3 Hz, 1H), 2.34 – 2.17 (m, 2H), 1.69 (s, 3H), 1.61 (p, J = 7.4 Hz, 2H), 1.35 – 1.28 (m, 4H), 0.89 (t, J = 6.9 Hz, 3H). **^{13}C NMR** (100 MHz, $CDCl_3$): δ 177.9, 157.5, 148.6, 121.1, 107.7, 105.4, 37.0, 34.0, 31.3, 30.0, 27.9, 27.5, 24.6, 22.3, 14.0. **FT-IR** (ATR) ν_{max}: 3127, 2955, 2930, 2860, 2241, 1710, 1555, 1455, 1417, 1381, 1293, 1218, 1173, 1112, 1017, 949, 786, 725, 620, 498, 468, 428 cm^{-1}. **HRMS**-(DART) (m/z) calcd for $C_{15}H_{22}NO_3$ [M+H]$^+$: 264.1599; found: 264.1598.

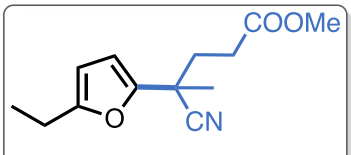

4n

Methyl 4-cyano-4-(5-ethylfuran-2-yl)pentanoate (4n). Following the general procedure above, using 2-ethylfuran (**2f**) (204 µL, 1.917 mmol), 4,4'-azobis(4-cyanovaleric acid)dimethyl ester (**1e**) (197 mg, 0.639 mmol), $Cu(OAc)_2$ (118 mg, 0.639 mmol) and 2,2,2-trichloroethanol (TCE) (0.78 mL), the crude reaction mixture was purified by flash column chromatography on silica gel with DCM/hexane (6:4) as eluent to afford the desired compound **4n** (107 mg, 0.455 mmol, 71%) as a colorless oil. **^{1}H NMR** (700 MHz, $CDCl_3$): δ 6.19 (d, J = 3.1 Hz, 1H), 5.90 (d, J = 3.2 Hz, 1H), 3.65 (s, 3H), 2.61 (qd, J = 7.5, 1.2 Hz, 2H), 2.49 (ddd, J = 16.3, 10.9, 5.5 Hz, 1H), 2.37 (ddd, J = 16.3, 10.9, 5.3 Hz, 1H), 2.30 (ddd, J = 13.9, 10.9, 5.3 Hz, 1H), 2.21 (ddd, J = 13.9, 10.9, 5.5 Hz, 1H), 1.68 (s, 3H), 1.20 (t, J = 7.6 Hz, 3H). **^{13}C NMR** (175 MHz, $CDCl_3$): δ 172.5, 158.5, 148.9, 121.2, 107.5, 104.6, 51.8, 37.0, 34.3, 30.1, 24.6, 21.3, 11.9. **FT-IR** (ATR) ν_{max}: 2975, 2942, 2880, 2851, 2239, 1737, 1606, 1556, 1437, 1383, 1370, 1298, 1260, 1239, 1199, 1174, 1111, 1015, 982, 954, 887, 843, 796, 781, 718, 628, 568, 482, 431 cm^{-1}. **HRMS**-(DART) (m/z) calcd for $C_{13}H_{18}NO_3$ [M+H]$^+$: 236.1286; found: 236.1282.

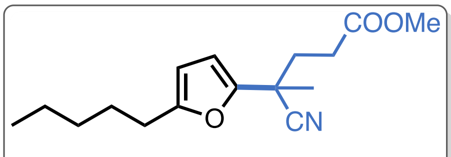

4o

Methyl 4-cyano-4-(5-pentylfuran-2-yl)pentanoate (4o). Following the general procedure above, using 2-pentylfuran (**2g**) (279 µL, 1.771 mmol), 4,4'-azobis(4-cyanovaleric acid)dimethyl ester (**1e**) (182 mg, 0.59 mmol), $Cu(OAc)_2$ (109 mg, 0.59 mmol) and 2,2,2-trichloroethanol (TCE) (0.72 mL), the crude reaction mixture was purified by flash column chromatography on silica gel with DCM/hexane (6:4) as eluent to afford the desired compound **4o** (126 mg, 0.454 mmol, 77%) as a yellow oil. **^{1}H NMR** (700 MHz, $CDCl_3$): δ 6.18 (d, J = 3.3 Hz, 1H), 5.89 (d, J = 3.3 Hz, 1H), 3.64 (s, 3H), 2.56 (t, J = 7.7 Hz, 2H), 2.47 (ddd, J = 16.3, 10.9, 5.5 Hz, 1H), 2.34 (ddd, J = 16.3, 10.9, 5.3 Hz, 1H), 2.29 (ddd, J = 13.9, 10.9, 5.2 Hz, 1H), 2.20 (ddd, J = 13.9, 10.9, 5.5 Hz, 1H), 1.66 (s, 3H), 1.60 (p, J = 7.4 Hz, 2H), 1.35 – 1.26 (m, 4H), 0.87 (t, J = 7.0 Hz, 3H). **^{13}C NMR** (175 MHz, $CDCl_3$): δ 172.4, 157.3, 148.8, 121.1, 107.5, 105.3, 51.7, 37.0, 34.2, 31.2, 30.0, 27.8, 27.4, 24.6, 22.3, 13.9. **FT-IR** (ATR) ν_{max}: 2953, 2931, 2860, 2238, 1740, 1606, 1556, 1454, 1437, 1380, 1299, 1199, 1174, 1112, 1017, 989, 951, 887, 843, 787, 721, 683 cm^{-1}. **HRMS**-(DART) (m/z) calcd for $C_{16}H_{24}NO_3$ [M+H]$^+$: 278.1756; found: 278.1755.

1.4 Procedure for the intramolecular cyclization of indole-containing azobis(alkylcarbonitriles) 5 for the construction of pyrido[1,2-*a*]indolones 6

1.4.1 Synthesis of indole-containing azobis(alkylcarbonitriles) 5a–c

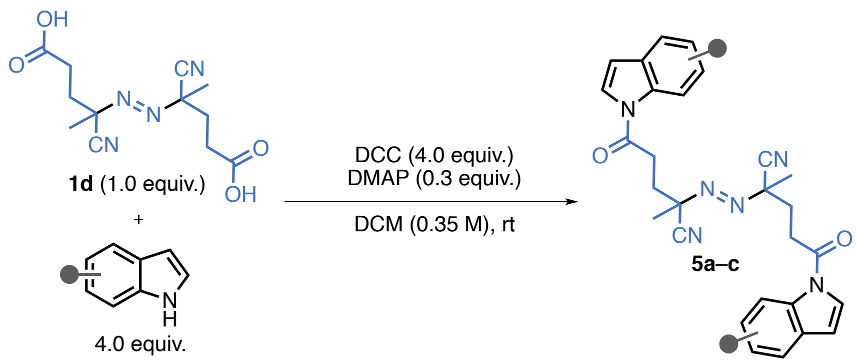

Following the procedure described by Wu and co-workers.[6] To a solution of indole (4.0 equiv.), 4,4'-azobis(4-cyanovaleric acid) (**1d**) (1.0 equiv.) and 4-dimethylaminopyridine (DMAP) (0.3 equiv.) in DCM (0.35 M) was added dicyclohexyl carbodiimide (DCC) (4.0 equiv.) in one portion at 0 °C. After 10 min at 0 °C, the resulting mixture was warmed up to room temperature and stirred for 20 h. The reaction was filtered through a pad of Celite and washed with DCM (10 mL). The filtrate was washed with saturated solution of $NaHCO_3$, dried over anhydrous Na_2SO_4 and concentrated under reduced pressure at room temperature. The crude product was purified by flash column chromatography on silica gel to afford the indole-containing azobis(alkylcarbonitriles) **5**.

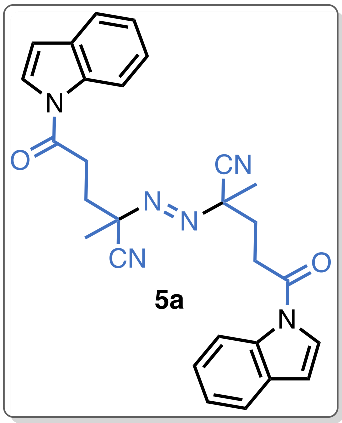

5a

2,2'-(Diazene-1,2-diyl)bis(5-(1*H*-indol-1-yl)-2-methyl-5-oxopentanenitrile) (5a). Following the general procedure above, using indole (891 mg, 6.993 mmol), 4,4'-azobis(4-cyanovaleric acid) (**1d**) (500 mg, 1.748 mmol), DMAP (64 mg, 0.524 mmol), DCC (1.443 g, 6.993 mmol) and DCM (5.0 mL), the crude reaction mixture was purified by flash column chromatography on silica gel with DCM/hexane (8:2) as eluent to afford the desired compound **5a** (437 mg, 0.913 mmol, 52%) as a pinkish solid. mp: 115-120 ºC (dec.). **^{1}H NMR** (400 MHz, $CDCl_3$): δ 8.41 (d, *J* = 8.2 Hz, 2H), 7.54 (dt, *J* = 7.7, 1.1 Hz, 2H), 7.42 (d, *J* = 3.9 Hz, 2H), 7.35 (td, *J* = 7.3, 1.2 Hz, 2H), 7.27 (td, *J* = 7.6, 1.1 Hz, 2H), 6.62 (dd, *J* = 3.8, 0.8 Hz, 2H), 3.19 (ddd, *J* = 16.4, 9.9, 5.7 Hz, 2H), 3.06 (ddd, *J* = 16.4, 10.1, 5.5 Hz, 2H), 2.74 (ddd, *J* = 14.4, 10.0, 5.6 Hz, 2H), 2.66 (ddd, *J* = 14.5, 10.1, 5.8 Hz, 2H), 1.77 (s, 6H). **^{13}C NMR** (100 MHz, $CDCl_3$): δ 168.7, 135.6, 130.4, 125.4, 124.0, 124.0, 121.0, 117.7, 116.5, 110.0, 71.7, 32.9, 30.6, 23.8. **FT-IR** (KBr) ν_{max}: 3053, 2994, 2932, 2852, 2241, 2116, 2013, 1708, 1693, 1628, 1605, 1583, 1538, 1455, 1398, 1342, 1308, 1206, 1154, 1113, 1085, 1014, 931, 880, 761, 748, 713, 617, 576, 481, 422 cm^{-1}. **HRMS**-(ESI) (*m*/*z*) calcd for $C_{28}H_{27}N_6O_2$ $[M+H]^+$: 479.2195; found: 479.2189.

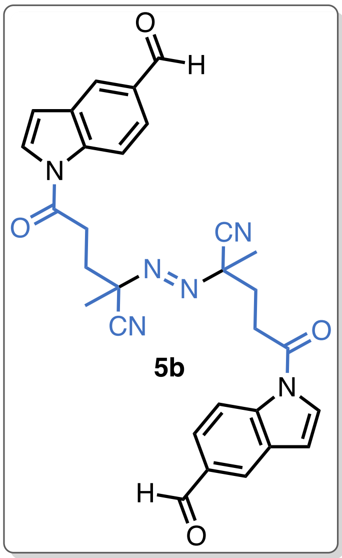

5b

2,2'-(Diazene-1,2-diyl)bis(5-(5-formyl-1*H*-indol-1-yl)-2-methyl-5-oxopentanenitrile) (5b). Following the general procedure above, using 5-formylindole (518 mg, 3.496 mmol), 4,4'-azobis(4-cyanovaleric acid) (**1d**) (250 mg, 0.874 mmol), DMAP (32 mg, 0.262 mmol), DCC (721 mg, 3.496 mmol) and DCM (2.5 mL), the crude reaction mixture was purified by flash column chromatography on silica gel with DCM/acetone (99:1) as eluent to afford the desired compound **5b** (157 mg, 0.294 mmol, 34%) as a white solid. mp: 110-115 ºC (dec.). **^{1}H NMR** (500 MHz, $CDCl_3$): δ 10.04 (s, 2H), 8.53 (d, *J* = 8.6 Hz, 2H), 8.07 (d, *J* = 1.6 Hz, 2H), 7.86 (dd, *J* = 8.6, 1.6 Hz, 2H), 7.56 (d, *J* = 3.8 Hz, 2H), 6.76 (d, *J* = 3.8 Hz, 2H), 3.24 (ddd, *J* = 16.9, 9.7, 5.8 Hz, 2H), 3.10 (ddd, *J* = 16.9, 9.9, 5.6 Hz, 2H), 2.76 (ddd, *J* = 15.1, 9.7, 5.5 Hz, 2H), 2.69 (ddd, *J* = 15.6, 9.7, 5.9 Hz, 2H), 1.79 (s, 6H). **^{13}C NMR** (125 MHz, $CDCl_3$): δ 191.9, 168.9, 139.0, 132.7, 130.6, 126.6, 125.8, 123.8, 117.6, 116.9, 110.4, 71.6, 32.8, 30.7, 23.8. **FT-IR** (ATR) ν_{max}: 2993, 2851, 2728, 2251, 1678, 1606, 1578, 1539, 1464, 1440, 1388, 1360, 1327, 1309, 1282, 1196, 1157, 1140, 1090, 929, 910, 823, 767, 727, 646, 602, 529, 480, 426 cm^{-1}. **HRMS**-(ESI) (*m*/*z*) calcd for $C_{30}H_{27}N_6O_4$ $[M+H]^+$: 535.2094; found: 535.2095.

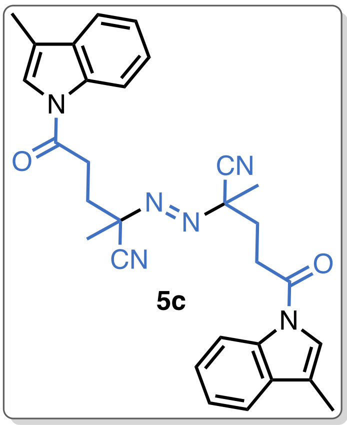

2,2'-(Diazene-1,2-diyl)bis(2-methyl-5-(3-methyl-1*H*-indol-1-yl)-5-oxopentanenitrile) (5c). Following the general procedure above, using 3-methylindole (917 mg, 6.993 mmol), 4,4'-azobis(4-cyanovaleric acid) (**1d**) (500 mg, 1.748 mmol), DMAP (64 mg, 0.524 mmol), DCC (1.443 mg, 6.993 mmol) and DCM (5.0 mL), the crude reaction mixture was purified by flash column chromatography on silica gel with DCM as eluent to afford the desired compound **5c** (200 mg, 0.395 mmol, 23%) as a white solid. mp: 135-138 ºC (dec.). **^{1}H NMR** (700 MHz, $CDCl_3$): δ 8.38 (bs, 2H), 7.45 (d, *J* = 7.7 Hz, 2H), 7.34 (ddd, *J* = 8.3, 7.2, 1.3 Hz, 2H), 7.27 (td, *J* = 7.8, 1.3 Hz, 2H), 7.17 (bs, 2H), 3.14 (ddd, *J* = 16.1, 10.5, 5.4 Hz, 2H), 2.99 (ddd, *J* = 16.2, 10.6, 5.3 Hz, 2H), 2.73 (ddd, *J* = 14.3, 10.4, 5.2 Hz, 2H), 2.65 (ddd, *J* = 14.4, 10.6, 5.4 Hz, 2H), 2.21 (d, *J* = 1.4 Hz, 6H), 1.78 (s, 6H). **^{13}C NMR** (175 MHz, $CDCl_3$): δ 168.2, 135.9, 131.4, 125.4, 123.7, 120.9, 119.5, 118.9, 117.7, 116.5, 71.8, 33.0, 30.6, 23.9, 9.6. **FT-IR** (ATR) ν_{max}: 3052, 2987, 2944, 2920, 2862, 2241, 1701, 1609, 1445, 1394, 1343, 1332, 1309, 1230, 1211, 1119, 1072, 1054, 1014, 930, 761, 749, 691, 646, 594, 557, 531, 487, 429 cm^{-1}. **HRMS**-(ESI) (*m*/*z*) calcd for $C_{30}H_{31}N_6O_2$ [M+H]$^+$: 507.2508; found: 507.2516.

1.4.2 6-*Exo-trig* radical cyclization of indole-containing azobis(alkylcarbonitriles) 6a–c

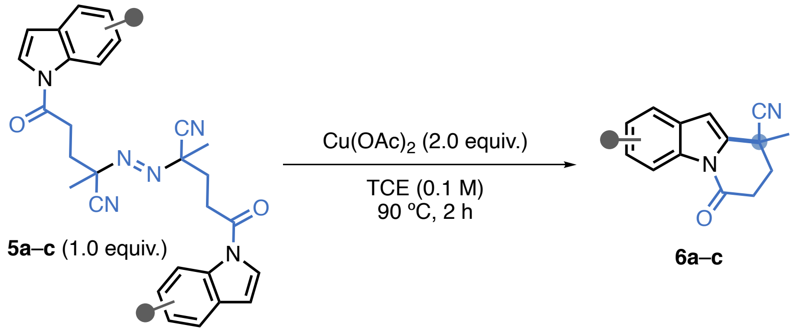

In a microwave reaction vial equipped with a stir bar was charged with indole-containing azobis(alkylcarbonitrile) **5a–c** (1.0 equiv.), $Cu(OAc)_2$ (2.0 equiv.) and 2,2,2-trichloroethanol (TCE) (0.1 M). The vial was sealed with a PTFE lined butyl rubber septum and aluminum crimp cap, and the solution was degassed by three consecutive freeze−pump−thaw cycles using liquid nitrogen and backfilled with pure argon. The mixture was stirred at 90 ºC in heating mantle for 2 h. After cooling to room temperature, the crude reaction mixture was extracted with a saturated solution of $NaHCO_3$ and DCM. The organic phase was dried over anhydrous Na_2SO_4 and evaporated under reduced pressure. The residue was purified by flash column chromatography on silica gel to afford the desired pyrido[1,2-*a*]indolone **6**.

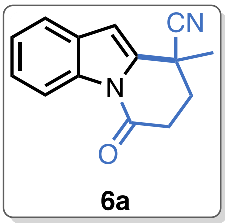

9-Methyl-6-oxo-6,7,8,9-tetrahydropyrido[1,2-*a*]indole-9-carbonitrile (6a). Following the general procedure above, using indole-containing azobis(alkylcarbonitrile) **5a** (100 mg, 0.205 mmol), $Cu(OAc)_2$ (76 mg, 0.41 mmol) and 2,2,2-trichloroethanol (TCE) (2.0 mL), the crude reaction mixture was purified by flash column chromatography on silica gel with hexane/EtOAc (75:25) as eluent to afford the desired compound **6a** (78 mg, 0.348 mmol, 85%) as a brown solid. mp: 85-90 ºC. **^{1}H NMR** (400 MHz, $CDCl_3$): δ 8.48 (dq, *J* = 8.2, 0.9 Hz, 1H), 7.55 (dt, *J* = 7.7, 1.1 Hz, 1H), 7.39 (td, *J* = 7.4, 1.4 Hz, 1H), 7.31 (td, *J* = 7.5, 1.2 Hz, 1H), 6.68 (s, 1H), 3.17 (ddd, *J* = 17.9, 11.8, 5.0 Hz, 1H), 2.95 (dt, *J* = 18.1, 4.5 Hz, 1H), 2.46 (dt, *J* = 13.6, 4.8 Hz, 1H), 2.19 (ddd, *J* = 13.3, 11.8, 4.4 Hz, 1H), 1.92 (s, 3H). **^{13}C NMR** (100 MHz, $CDCl_3$): δ 167.0, 135.9, 135.2, 128.5, 125.9, 124.6, 120.8, 120.7, 116.6, 107.1, 33.3, 32.7, 31.3, 25.1. **FT-IR** (ATR) ν_{max}: 2984, 2960, 2934, 2870, 2853, 2233, 1701, 1590, 1571, 1537, 1451, 1370, 1348, 1309, 1205, 1175, 1151, 1108, 1093, 1016, 988, 941, 867, 820, 756, 717, 670, 581, 560, 541, 480, 450, 422 cm^{-1}. **HRMS**-(DART) (*m*/*z*) calcd for $C_{14}H_{13}N_2O$ [M+H]$^+$: 225.1027; found: 225.1031.

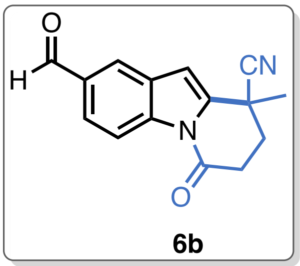

2-Formyl-9-methyl-6-oxo-6,7,8,9-tetrahydropyrido[1,2-*a*]indole-9-carbonitrile (6b). Following the general procedure above, using indole-containing azobis(alkylcarbonitrile) **5b** (150 mg, 0.275 mmol), $Cu(OAc)_2$ (102 mg, 0.55 mmol) and 2,2,2-trichloroethanol (TCE) (2.7 mL), the crude reaction mixture was purified by flash column chromatography on silica gel with hexane/EtOAc (6:4) as eluent to afford the desired compound **6b** (86 mg, 0.341 mmol, 62%) as an orange solid. mp: 195-200 ºC. **^{1}H NMR** (500 MHz, $CDCl_3$): δ 10.07 (s, 1H), 8.62 (d, *J* = 8.5 Hz, 1H), 8.09 (d, *J* = 1.1 Hz, 1H), 7.91 (dd, *J* = 8.6, 1.6 Hz, 1H), 6.80 (s, 1H), 3.21 (ddd, *J* = 18.2, 11.9, 5.0 Hz, 1H), 3.01 (dt, *J* = 18.1, 4.4 Hz, 1H), 2.50 (dt, *J* = 13.6, 4.8 Hz, 1H), 2.24 (ddd, *J* = 13.7, 11.9, 4.4 Hz, 1H), 1.95 (s, 3H). **^{13}C NMR** (125 MHz, $CDCl_3$): δ 191.8, 167.0, 138.6, 137.9, 133.2, 128.8, 127.2, 123.3, 120.3, 117.1, 107.4, 33.1, 32.8, 31.3, 25.0. **FT-IR** (ATR) ν_{max}: 2921, 2850, 2816, 2724, 2236, 1717, 1683, 1607, 1587, 1538, 1462, 1440, 1383, 1338, 1296, 1194, 1175, 1156, 1114, 992, 928, 891, 825, 768, 715, 676, 624, 596, 549, 454, 425 cm^{-1}. **HRMS**-(DART) (*m*/*z*) calcd for $C_{15}H_{13}N_2O_2$ [M+H]$^+$: 253.0977; found: 253.0966.

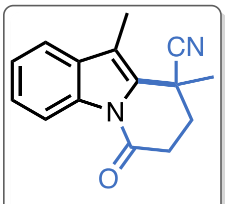

6c

9,10-Dimethyl-6-oxo-6,7,8,9-tetrahydropyrido[1,2-*a*]indole-9-carbonitrile (6c). Following the general procedure above, using indole-containing azobis(alkylcarbonitrile) **5c** (94 mg, 0.182 mmol), $Cu(OAc)_2$ (66 mg, 0.364 mmol) and 2,2,2-trichloroethanol (TCE) (1.8 mL), the crude reaction mixture was purified by flash column chromatography on silica gel with hexane/EtOAc (75:25) as eluent to afford the desired compound **6c** (42 mg, 0.176 mmol, 48%) as a yellow oil. **^{1}H NMR** (700 MHz, $CDCl_3$): δ 8.46 (d, *J* = 8.2 Hz, 1H), 7.51 (d, *J* = 7.8 Hz, 1H), 7.38 (td, *J* = 7.3, 1.1 Hz, 1H), 7.33 (td, *J* = 7.5, 1.1 Hz, 1H), 2.94 (ddd, *J* = 17.6, 6.4, 4.7 Hz, 1H), 2.87 (ddd, *J* = 17.6, 10.4, 4.7 Hz, 1H), 2.60 (ddd, *J* = 13.6, 10.4, 4.8 Hz, 1H), 2.46 (s, 3H), 2.32 (ddd, *J* = 13.7, 6.5, 4.8 Hz, 1H), 1.85 (s, 3H). **^{13}C NMR** (175 MHz, $CDCl_3$): δ 166.6, 134.2, 130.5, 129.2, 126.0, 124.3, 121.2, 118.5, 116.7, 116.3, 33.3, 31.7, 30.0, 24.9, 9.2. **FT-IR** (ATR) ν_{max}: 3051, 2982, 2923, 2871, 2237, 1701, 1607, 1453, 1388, 1362, 1339, 1315, 1211, 1200, 1166, 1156, 1141, 1098, 1017, 932, 912, 827, 809, 748, 678, 586, 562, 541, 450, 426 cm^{-1}. **HRMS**-(DART) (*m*/*z*) calcd for $C_{15}H_{15}N_2O$ $[M+H]^+$: 239.1184; found: 239.1179.

1.5 Derivatization of 1-(benzofuran-2-yl)cyclohexane-1-carbonitrile (3a)

1.5.1 Hydrogenation of 3a over Raney Ni to Boc-amine 7

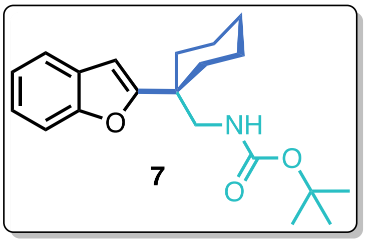

***Tert*-butyl ((1-(benzofuran-2-yl)cyclohexyl)methyl)carbamate (7).** Following the procedure described by Reisman and Kadunce.[7] Raney Ni (333 mg) was rinsed with dry MeOH 3 times to remove excess water and added to a flame-dried flask. To this was added dry MeOH (25 mL), 1-(benzofuran-2-yl)cyclohexane-1-carbonitrile (**3a**) (100 mg, 0.444 mmol), and Boc anhydride (147 mg, 0.666 mmol). The flask was purged with N_2 for 15 min, then flushed with two balloons of H_2. The flask was equipped with a balloon of H_2 and stirred for 12 h. The reaction was then filtered over Celite with EtOAc. The reaction mixture was then evaporated under vacuum and the residue was purified by flash column chromatography on silica gel with hexane/EtOAc (9:1) as eluent to afford **7** (0.107 g, 0.325 mmol, 73%) as a colorless oil. **^{1}H NMR** (400 MHz, $CDCl_3$): δ 7.52 (dd, *J* = 7.0, 1.8 Hz, 1H), 7.44 (d, *J* = 7.8 Hz, 1H), 7.27 – 7.17 (m, 2H), 6.51 (s, 1H), 4.42 (bs, 1H), 3.37 (d, *J* = 5.7 Hz, 2H), 2.20 – 2.03 (m, 2H), 1.70 – 1.50 (m, 5H), 1.49 – 1.30 (m, 12H). **^{13}C NMR** (100 MHz, $CDCl_3$): δ 162.1, 156.1, 154.6, 128.6, 123.4, 122.5, 120.4, 111.0, 103.5, 79.1, 49.3, 41.6, 32.5, 28.3, 26.0, 22.1. **FT-IR** (ATR) ν_{max}: 3364, 2975, 2931, 2856, 1699, 1577, 1504, 1453, 1391, 1364, 1245, 1163, 1045, 1006, 964, 928, 880, 851, 801, 775, 749, 738, 702, 585, 489, 429 cm^{-1}. **HRMS**-(DART) (*m*/*z*) calcd for $C_{20}H_{28}NO_3$ $[M+H]^+$: 330.2069; found: 330.2071.

1.5.2 DIBAL-H reduction of 3a to carboxaldehyde 8

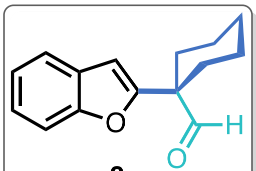

1-(Benzofuran-2-yl)cyclohexane-1-carbaldehyde (8). Following the procedure described by Feng and co-workers.[8] In a flame-dried flask with 1-(benzofuran-2-yl)cyclohexane-1-carbonitrile (**3a**) (196 mg, 0.870 mmol) under argon atmosphere, 10.0 mL of anhydrous DCM was added. The solution was cooled to −78 °C and DIBAL-H (1.0 M in hexane, 1.0 mL, 1.044 mmol) was added dropwise, the resulting mixture was warmed up to room temperature and stirred for 2 h. The reaction was quenched with HCl (aq. 1.0 M) and extracted with DCM. The organic phase was washed with brine, dried over anhydrous Na_2SO_4, and evaporated under vacuum. The residue was purified by flash column chromatography on silica gel with hexane/EtOAc (9:1) as eluent to afford **8** (0.152 g, 0.666 mmol, 77%) as a colorless oil. **^{1}H NMR** (400 MHz, $CDCl_3$): δ 9.52 (s, 1H), 7.55 (dd, *J* = 7.5, 1.2 Hz, 1H), 7.45 (dd, *J* = 8.2, 1.0 Hz, 1H), 7.26 (td, *J* = 7.3, 1.3 Hz, 1H), 7.22 (td, *J* = 7.4, 1.2 Hz, 1H), 6.62 (d, *J* = 1.0 Hz, 1H), 2.12 (t, *J* = 6.0 Hz, 4H), 1.64 – 1.56 (m, 4H), 1.52 – 1.46 (m, 2H). **^{13}C NMR** (100 MHz, $CDCl_3$): δ 199.9, 156.7, 155.1, 128.3, 124.1, 122.8, 120.8, 111.2, 104.7, 52.8, 29.2, 25.5, 22.1. **FT-IR** (film) ν_{max}: 2929, 2854, 1728, 1663, 1575, 1452, 1375, 1348, 1303, 1255, 1180, 1166, 1110, 1090, 1027, 1008, 928, 881, 800, 748, 738, 613, 528, 433 cm^{-1}. **HRMS**-(DART) (*m*/*z*) calcd for $C_{15}H_{17}O_2$ $[M+H]^+$: 229.1228; found: 229.1229.

1.5.3 MeMgBr addition on 3a to ketone 9

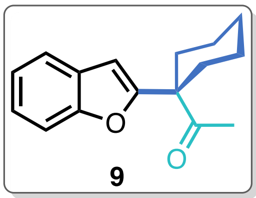

1-(1-(Benzofuran-2-yl)cyclohexyl)ethan-1-one (9). Following the procedure described by Huo and co-workers.[2] To a solution of 1-(benzofuran-2-yl)cyclohexane-1-carbonitrile (**3a**) (91 mg, 0.404 mmol) in toluene (4.8 mL) was added dropwise MeMgBr (3.0 M in Et_2O, 0.404 mL, 1.212 mmol) at room temperature under argon atmosphere. The reaction was stirred at 60 °C for 1 h. After cooling to room temperature, a saturated solution of NH_4Cl was added to the reaction and the mixture was acidified with 1N HCl. The resulting mixture was refluxed for 1 h, and it was extracted with ether. The organic phase was washed with a saturated solution of $NaHCO_3$ and brine, dried over anhydrous Na_2SO_4, and evaporated under reduced pressure. The residue was purified by flash column chromatography on silica gel with hexane/EtOAc (98:2) as eluent to afford **9** (33 mg, 0.136 mmol, 34%) as a pale-yellow oil. **^{1}H NMR** (400 MHz, $CDCl_3$): δ 7.55 (dd, *J* = 7.7, 1.1 Hz, 1H), 7.46 (dd, *J* = 7.2, 0.7 Hz, 1H), 7.29 – 7.19 (m, 2H), 6.63 (d, *J* = 0.9 Hz, 1H), 2.23 – 2.14 (m, 2H), 2.13 – 2.05 (m, 2H), 2.05 (s, 3H), 1.67 – 1.44 (m, 6H). **^{13}C NMR** (100 MHz, $CDCl_3$): δ 208.2, 158.9, 154.7, 128.5, 123.9, 122.7, 120.7, 111.2, 104.3, 54.3, 31.4, 25.7, 25.6, 22.5. **FT-IR** (film) ν_{max}: 2936, 2857, 1711, 1574, 1453, 1420, 1352, 1304, 1256, 1207, 1178, 1122, 1027, 1006, 947, 876, 802, 751, 682, 647 cm^{-1}. **HRMS**-(DART) (*m*/*z*) calcd for $C_{16}H_{19}O_2$ $[M+H]^+$: 243.1385; found: 243.1378.

1.5.4 Cycloaddition of azide and 3a to tetrazole 10

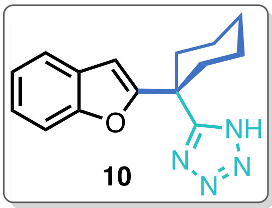

10

5-(1-(benzofuran-2-yl)cyclohexyl)-1*H*-tetrazole (10). Following the procedure described by Harusawa and co-workers.[9] NaN_3 (138mg, 2.117 mmol) and $Et_3N{\cdot}HCl$ (340 mg, 2.47 mmol) were added to a solution of 1-(benzofuran-2-yl)cyclohexane-1-carbonitrile (**3a**) (159 mg, 0.706 mmol) in DMF (2.0 mL). The reaction mixture was exposed to microwave irradiation (130 ºC, 300 W) for 3 h. EtOAc and 6N HCl were added to the mixture at 0 °C. The organic phase was separated, washed with 2N HCl, dried, and evaporated under reduced pressure. The residue was purified by flash column chromatography on silica gel with DCM/MeOH (95:5) as eluent to afford **10** (170 mg, 0.634 mmol, 90%) as colorless crystals. mp: 150-155 ºC. **^{1}H NMR** (400 MHz, $CDCl_3$): δ 10.09 (bs, 1H), 7.44 (d, *J* = 7.1 Hz, 1H), 7.33 (d, *J* = 7.2 Hz, 1H), 7.22 – 7.11 (m, 2H), 6.60 (s, 1H), 2.55 – 2.36 (m, 4H), 1.65 – 1.37 (m, 6H). **^{13}C NMR** (100 MHz, $CDCl_3$): δ 160.7, 158.0, 154.6, 128.0, 124.3, 122.9, 121.0, 111.2, 104.1, 40.0, 34.0, 25.2, 22.1. **FT-IR** (ATR) ν_{max}: 3088, 2935, 2859, 2709, 2613, 1583, 1542, 1447, 1403, 1350, 1305, 1257, 1247, 1166, 1082, 1042, 1025, 995, 952, 917, 902, 879, 842, 810, 792, 749, 660, 582, 503, 476, 439 cm^{-1}. **HRMS**-(DART) (*m*/*z*) calcd for $C_{15}H_{17}N_4O$ $[M+H]^+$: 269.1402; found: 269.1392.

1.6 Radical trapping experiments

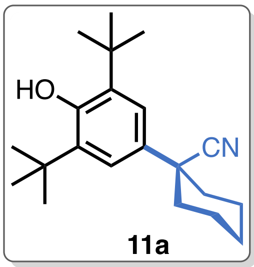

11a

1-(3,5-di-*tert*-butyl-4-hydroxyphenyl)cyclohexane-1-carbonitrile (11a). Following the general procedure 1.2, using 2,6-di-*tert*-butylphenol (85 mg, 0.41 mmol), 1,1'-azobis(cyclohexanecarbonitrile) (**1a**) (204 mg, 0.82 mmol), $Cu(OAc)_2$ (76 mg, 0.41 mmol) and 2,2,2-trichloroethanol (TCE) (1.0 mL), the crude reaction mixture was purified by flash column chromatography on silica gel with hexane/DCM (65:35) as eluent to afford the desired compound **11a** (104 mg, 0.332 mmol, 81%) as yellow crystals. mp: 110-115 ºC. **^{1}H NMR** (500 MHz, $CDCl_3$): δ 7.27 (s, 2H), 5.22 (s, 1H), 2.15 (d, *J* = 13.2 Hz, 2H), 1.93 – 1.64 (m, 8H), 1.45 (s, 18H). **^{13}C NMR** (125 MHz, $CDCl_3$): δ 153.2, 136.0, 132.1, 123.2, 122.2, 44.0, 37.6, 34.6, 30.2, 25.0, 23.7. **FT-IR** (ATR) ν_{max}: 3568, 3005, 2966, 2945, 2929, 2860, 2237, 1588, 1434, 1388, 1358, 1318, 1304, 1240, 1213, 1118, 1024, 941, 886, 868, 811, 773, 734, 687, 632, 621, 536, 479, 439 cm^{-1}. **HRMS**-(DART) (*m*/*z*) calcd for $C_{21}H_{32}NO$ $[M+H]^+$: 314.2483; found: 314.2488.

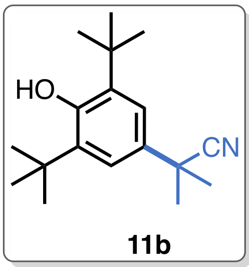

11b

2-(3,5-di-*tert*-butyl-4-hydroxyphenyl)-2-methylpropanenitrile (11b). Following the general procedure 1.2, using 2,6-di-*tert*-butylphenol (85 mg, 0.41 mmol), 2,2'-azobis(2-methylpropionitrile) (**1b**) (202 mg, 1.23 mmol), $Cu(OAc)_2$ (76 mg, 0.41 mmol) and 2,2,2-trichloroethanol (TCE) (1.0 mL), the crude reaction mixture was purified by flash column chromatography on silica gel with hexane/DCM (65:35) as eluent to afford the desired compound **11b** (72 mg, 0.263 mmol, 64%) as brown crystals. mp: 115-120 ºC. **^{1}H NMR** (400 MHz, $CDCl_3$): δ 7.26 (s, 2H), 5.24 (s, 1H), 1.71 (s, 6H), 1.46 (s, 18H). **^{13}C NMR** (100 MHz, $CDCl_3$): δ 153.1, 136.1, 131.9, 125.1, 121.7, 36.9, 34.6, 30.2, 29.4. **FT-IR** (ATR) ν_{max}: 3573, 2955, 2922, 2871, 2239, 1809, 1766, 1722, 1639, 1602, 1566, 1453, 1431, 1386, 1359, 1304, 1227, 1197, 1105, 1022, 931, 880, 809, 774, 702, 639, 487, 433 cm^{-1}. **HRMS**-(DART) (*m*/*z*) calcd for $C_{18}H_{28}NO$ $[M+H]^+$: 274.2170; found: 274.2173.

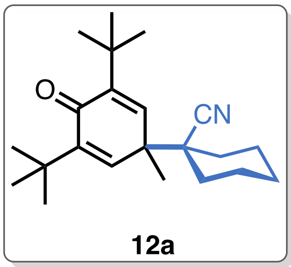

12a

3',5'-di-*tert*-butyl-1'-methyl-4'-oxo-[1,1'-bi(cyclohexane)]-2',5'-diene-1-carbonitrile (12a). Following the general procedure 1.2, using dibutylhydroxytoluene (90 mg, 0.41 mmol), 1,1'-azobis(cyclohexanecarbonitrile) (**1a**) (204 mg, 0.82 mmol), $Cu(OAc)_2$ (76 mg, 0.41 mmol) and 2,2,2-trichloroethanol (TCE) (1.0 mL), the crude reaction mixture was purified by flash column chromatography on silica gel with hexane/DCM (65:35) as eluent to afford the desired compound **12a** (98 mg, 0.299 mmol, 73%) as yellow crystals. mp: 85-90 ºC. **^{1}H NMR** (400 MHz, $CDCl_3$): δ 6.54 (s, 2H), 1.89 (d, *J* = 15.1 Hz, 2H), 1.82 – 1.51 (m, 6H), 1.37 (s, 3H), 1.22 (s, 18H), 1.25 – 1.15 (m, 2H). **^{13}C NMR** (100 MHz, $CDCl_3$): δ 186.0, 149.0, 141.1, 121.6, 47.2, 43.1, 35.1, 30.6, 29.4, 25.1, 23.4, 22.2. **FT-IR** (ATR) ν_{max}: 2998, 2950, 2864, 2228, 1794, 1660, 1643, 1484, 1453, 1389, 1376, 1365, 1342, 1309, 1248, 1201, 1176, 1159, 1082, 1027, 932, 914, 880, 815, 740, 655 cm^{-1}. **HRMS**-(neat) (*m*/*z*) calcd for $C_{22}H_{34}NO$ $[M+H]^+$: 328.2640; found: 328.2626.

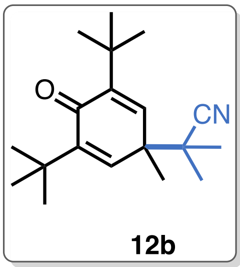

12b

2-(3,5-di-*tert*-butyl-1-methyl-4-oxocyclohexa-2,5-dien-1-yl)-2-methylpropanenitrile (12b). Following the general procedure 1.2, using dibutylhydroxytoluene (90 mg, 0.41 mmol), 2,2'-azobis(2-methylpropionitrile) (**1b**) (206 mg, 1.23 mmol), $Cu(OAc)_2$ (76 mg, 0.41 mmol) and 2,2,2-trichloroethanol (TCE) (1.0 mL), the crude reaction mixture was purified by flash column chromatography on silica gel with hexane/DCM (65:35) as eluent to afford the desired compound **12b** (108 mg, 0.376 mmol, 92%) as yellow crystals. mp: 50-55 ºC. **^{1}H NMR** (400 MHz, $CDCl_3$): δ 6.53 (s, 2H), 1.40 (s, 3H), 1.29 (s, 6H), 1.24 (s, 18H). **^{13}C NMR** (100 MHz, $CDCl_3$): δ 185.5, 149.3, 140.7, 123.4, 42.7, 39.8, 35.1, 29.4, 22.9, 22.6. **FT-IR** (film) ν_{max}: 2958, 2912, 2870, 2233, 1804, 1662, 1484, 1460, 1390, 1365, 1308, 1249, 1204, 1175, 1156, 1079, 1057, 1022, 931, 916, 902, 880, 816, 741, 652 cm^{-1}. **HRMS**-(DART) (*m*/*z*) calcd for $C_{19}H_{30}NO$ $[M+H]^+$: 288.2327; found: 288.2316.

1.7 X-ray crystallographic information of 3n

The structure of **3n** was determined by X-ray diffraction on a Bruker D8 Venture diffractometer using CuKα radiation (λ= 1.5417 Å). Proper crystals were obtained in hexane/dichloromethane (30/1) by slow evaporation of the solvent at room temperature. The obtained data sets were processed with APEX3 software.[10] The phase problem was solved by direct methods using SHELXS-2014.[11] Parameters of obtained models were refined by full-matrix least-squares on F^2 using SHELXL-2016/4.[12] Non-hydrogen atoms were refined anisotropically, and hydrogen atoms were allowed to ride on the respective atoms. Calculations were performed using SHELXL-2014/7.[13] Figures were prepared with ORTEP-3 (ver. 2014.1).[14] Molecular geometry of compound **3n** observed in the crystal structure is shown in Figure S1, and crystal data and structure refinement results are shown in Table S1. Crystallographic data for the structure presented in this paper has been deposited with the Cambridge Crystallographic Data Centre as a supplementary publication number. CCDC 2304685 (**3n**). Copies of the data can be obtained, free of charge, on application to CCDC, 12 Union Road, Cambridge CB2 1EZ, UK, (fax: +44-(0)1223-336033 or e-mail: deposit@ccdc.cam.ac.uk/data_request/cif).

Table S1. Crystal data and structure refinement results for compound **3n**.

Compound	1-(5-Formyl-1*H*-pyrrol-2-yl) cyclohexane-1-carbonitrile (**3n**)
Empirical moiety formula	$C_{12}H_{14}N_2O$
Formula weight (g/mol)	202.25
Temperature (K)	150
Wavelength (Å)	1.54178
Crystal system	Monoclinic
Space group	$P2_1/c$
Unite cell dimensions	a= 13.3135(3) Å<br>b= 6.8050(2) Å<br>c= 12.2112(3) Å<br>α= 90º<br>ß= 91.7890(12)º<br>γ= 90º
Volume (Å^3)	1105.78(5)
Z	4
D_{calc} (g/cm^3)	1.215
μ (mm^{-1})	0.629
F(000)	432
Crystal size (mm^3)	0.084 × 0.346 × 0.403
Θ range	3.32º to 68.23º
Index ranges	–15 ≤h ≤ 16,<br>–8 ≤ k ≤ 8,<br>–14 ≤ l ≤ 13
Refl. collected	6978
Refl. unique	2014
Absorption correction	Multi-scan
Refinement method	Full-matrix least-squares on F^2
Data/restraints/parameters	2014/0/139
GooF on F2	1.784
Final R indices [I>2sigma(I)]	R1= 0.0375,<br>wR2= 0.1049
R indices (all data)	R1= 0.0418,<br>wR2= 0.1095
$\Delta\rho_{max}$, $\Delta\rho_{min}$ (e·Å^{-3})	0.239 and –0.173

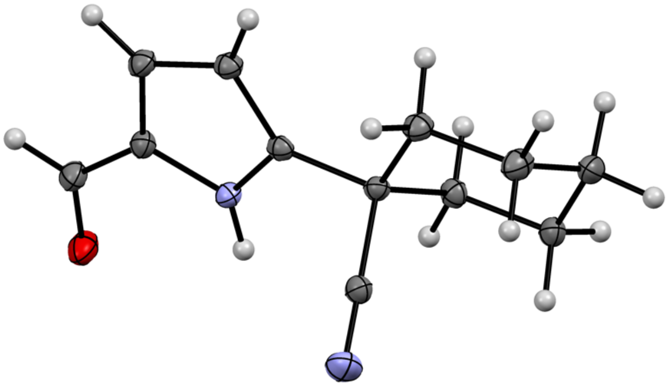

Figure S1. Molecular geometry observed in the crystal structure of compound **3n**. Displacement ellipsoids of non-hydrogen atoms are drawn at the 30% probability level. Hydrogen atoms are presented as small spheres with an arbitrary radius.

Part II ^{1}H and ^{13}C NMR spectra

^{1}H NMR (300 MHz, $CDCl_3$)

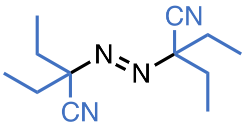

1c

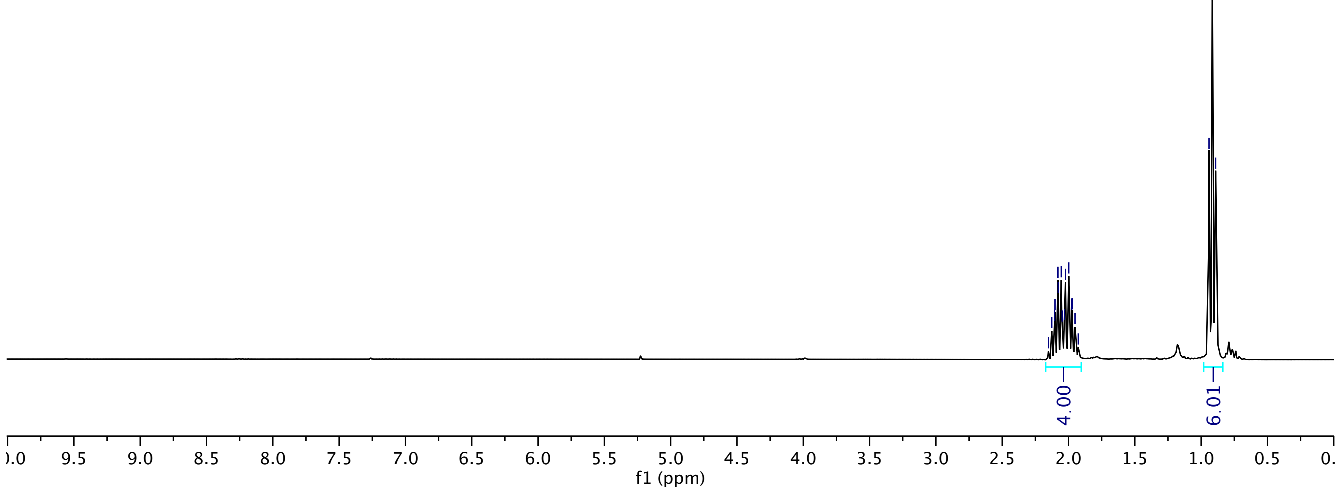

^{13}C NMR (75 MHz, $CDCl_3$)

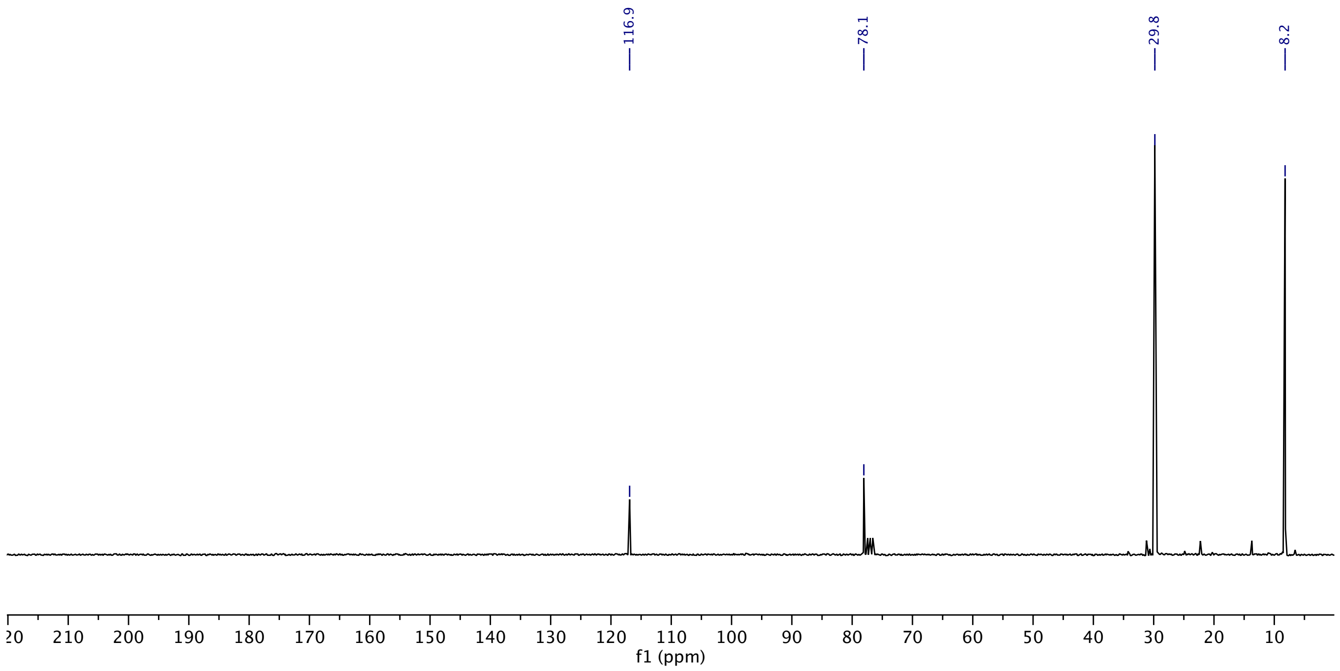

^{1}H NMR (300 MHz, $CDCl_3$)

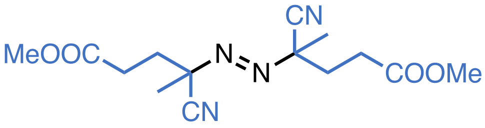

1e

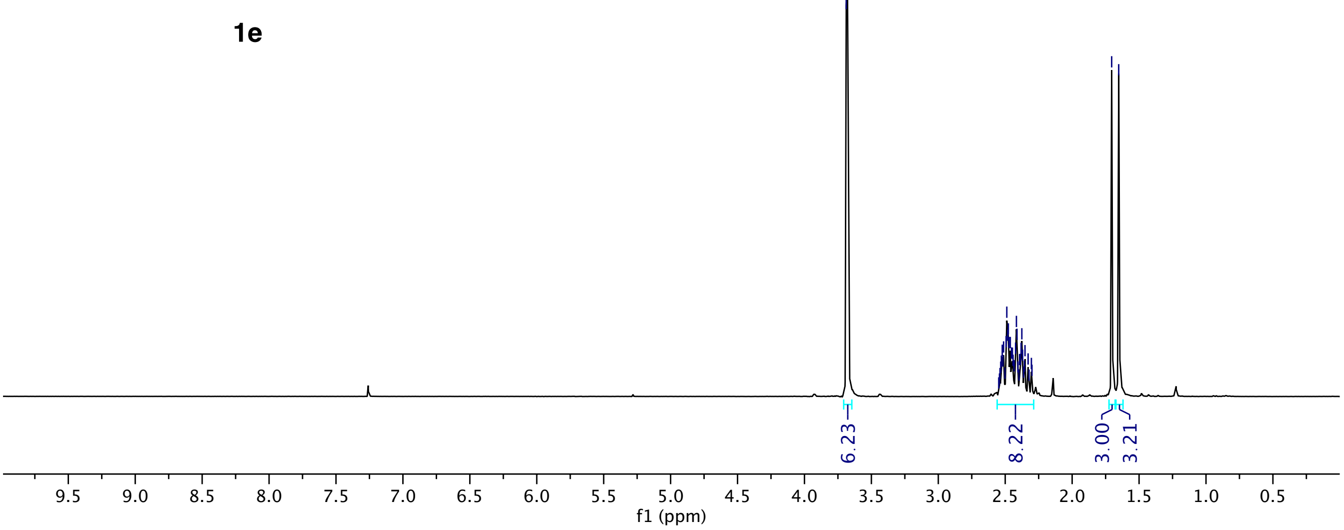

^{13}C NMR (75 MHz, $CDCl_3$)

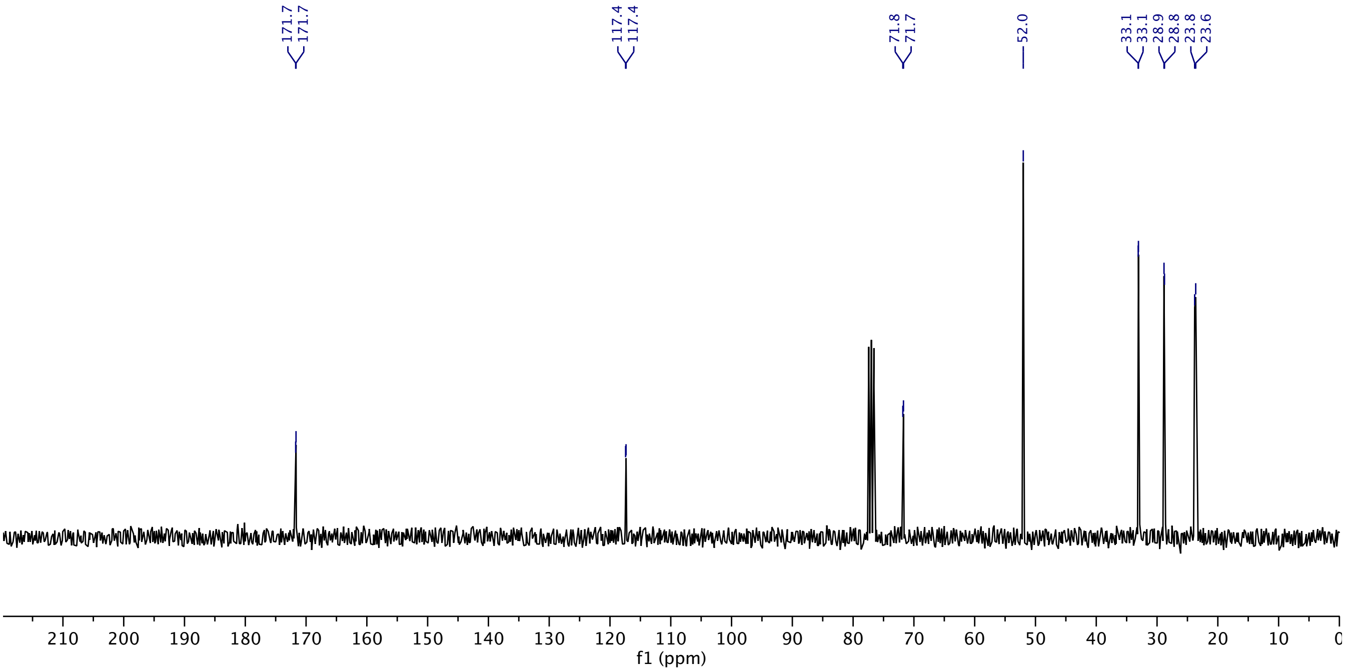

^{1}H NMR (400 MHz, $CDCl_3$)

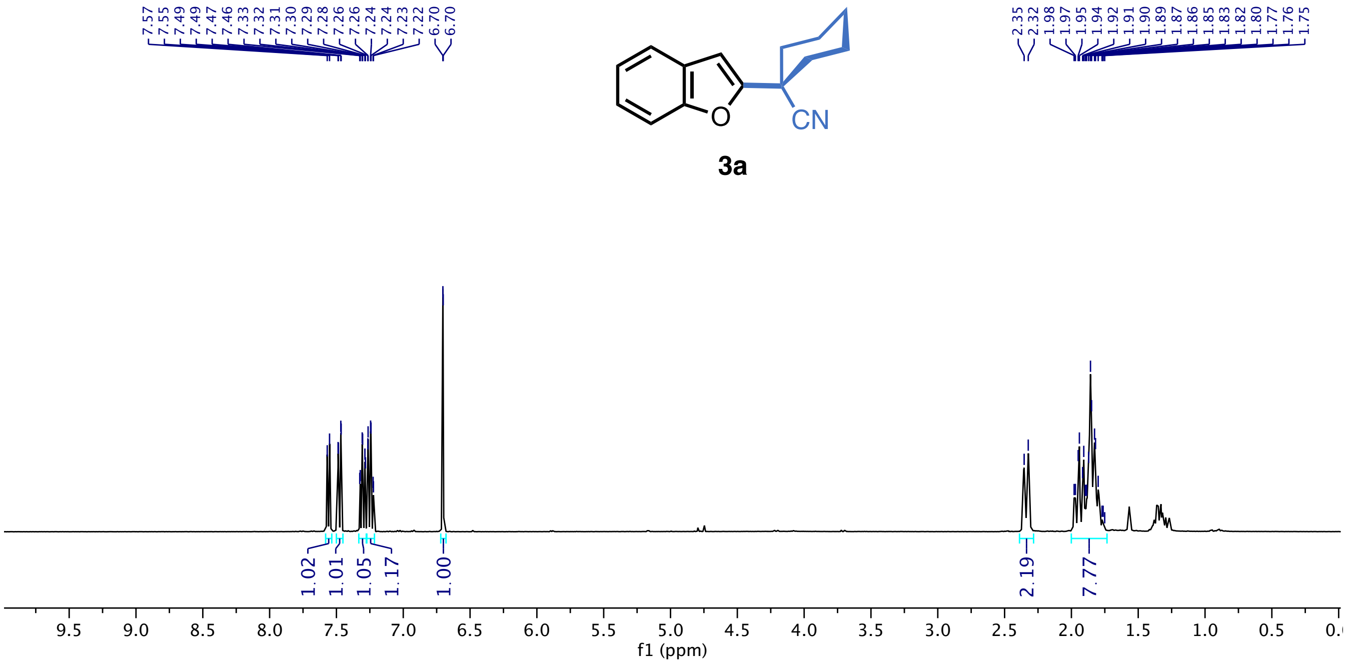

^{13}C NMR (100 MHz, $CDCl_3$)

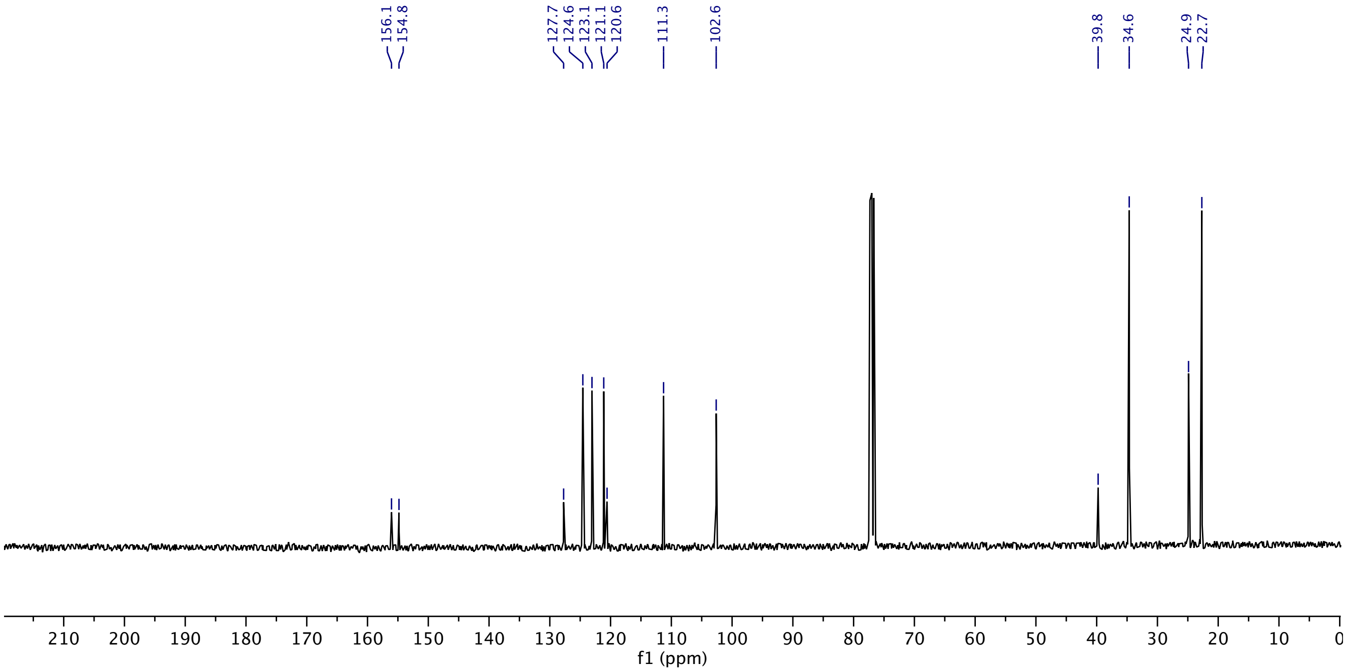

^{1}H NMR (400 MHz, $CDCl_3$)

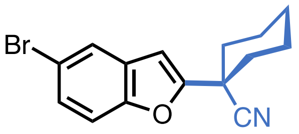

3b

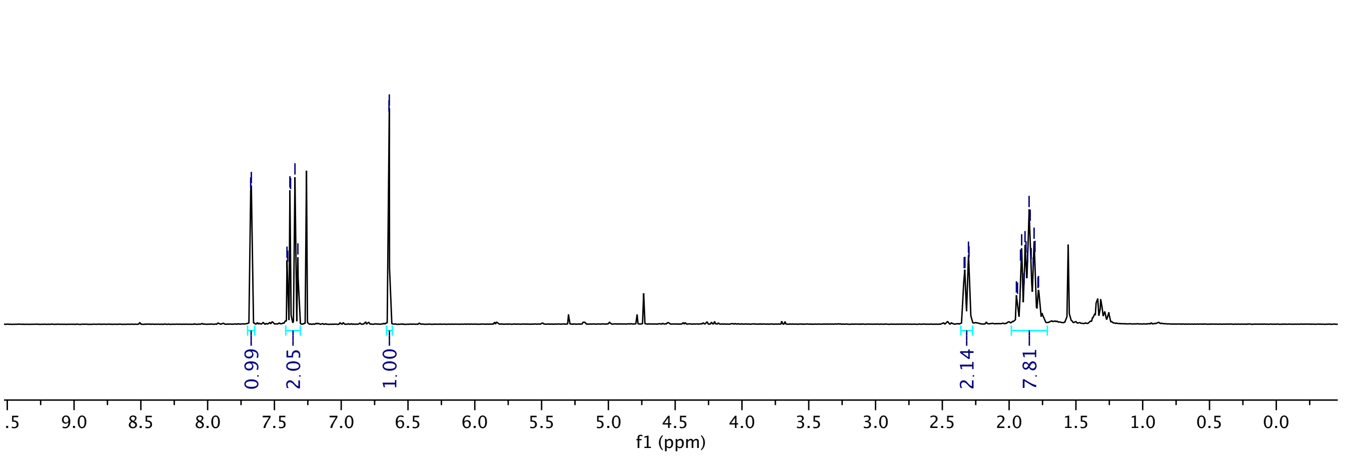

^{13}C NMR (100 MHz, $CDCl_3$)

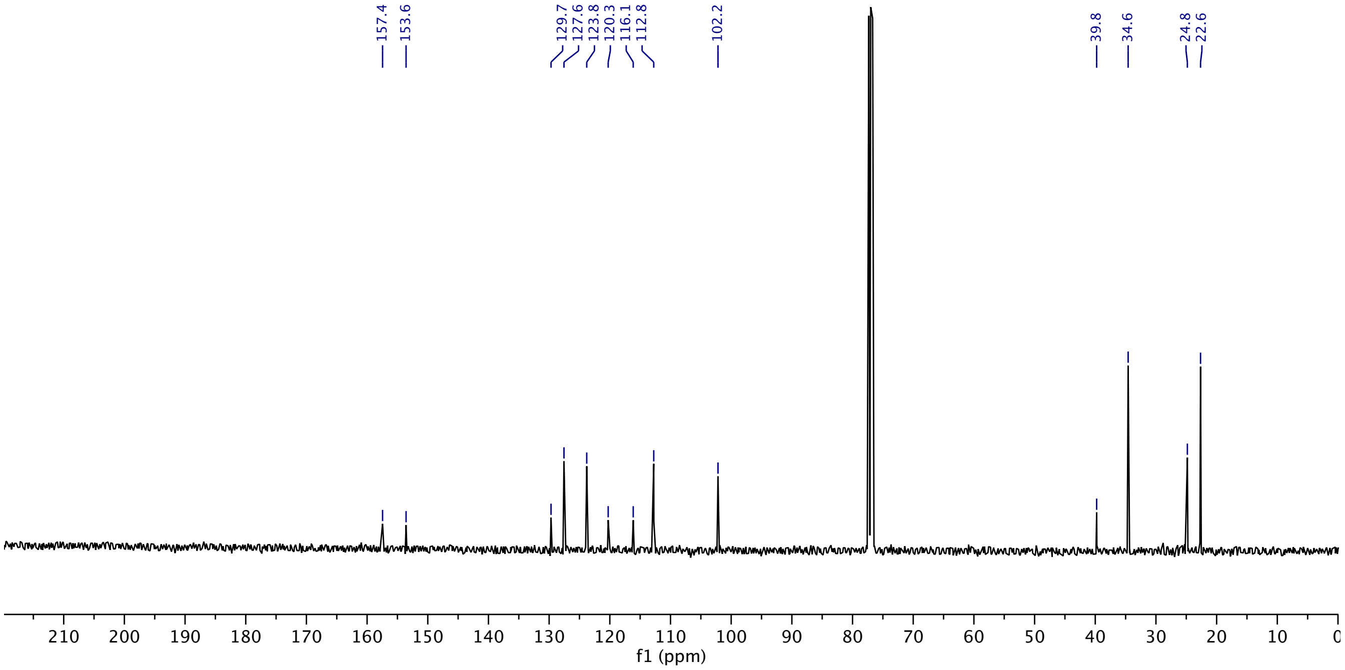

^{1}H NMR (400 MHz, $CDCl_3$)

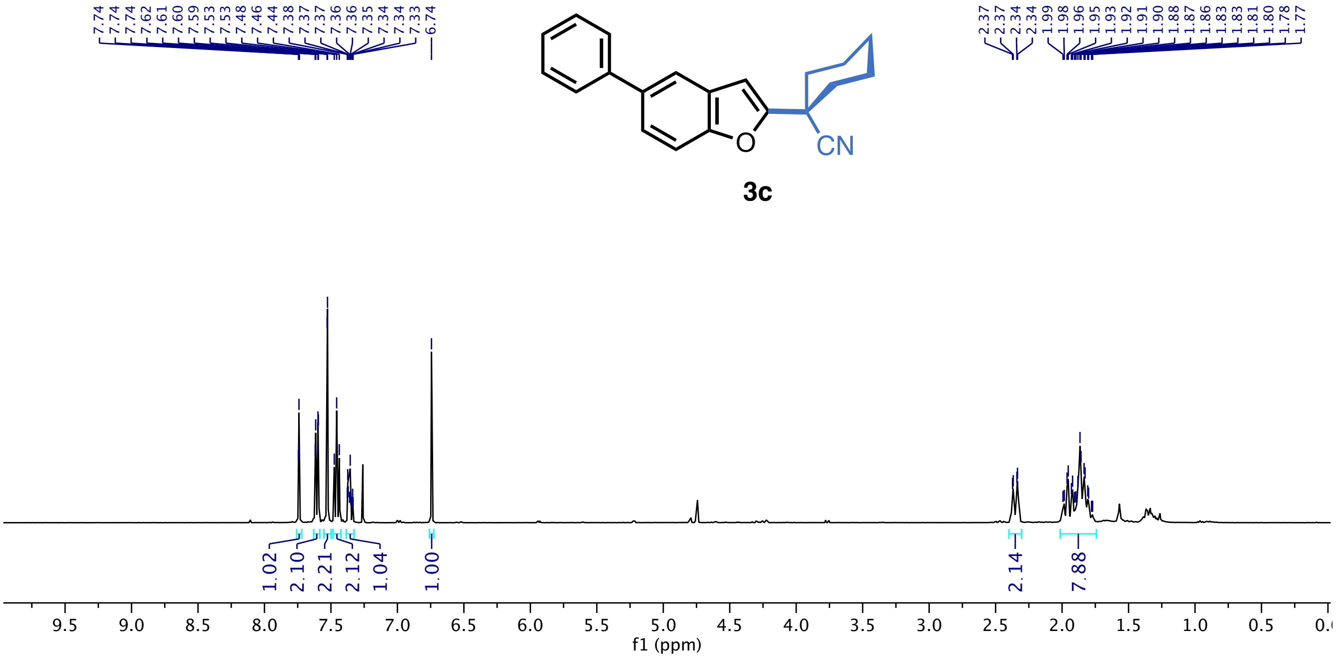

^{13}C NMR (100 MHz, $CDCl_3$)

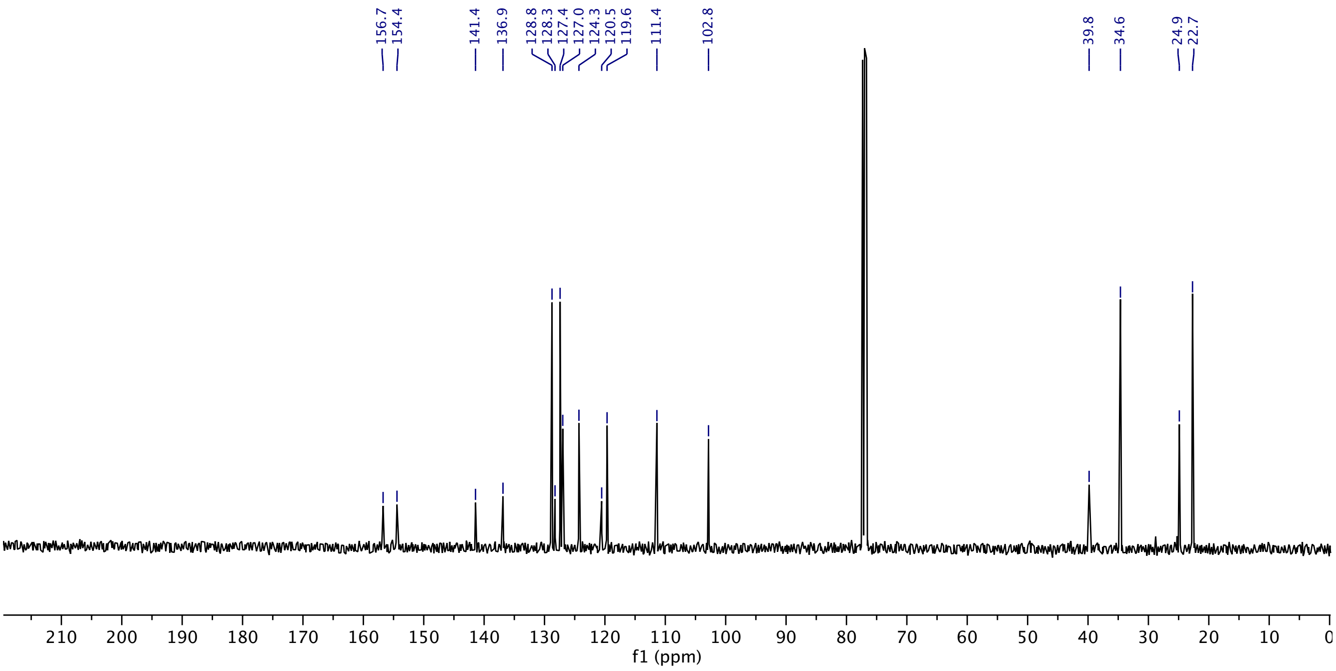

^{1}H NMR (400 MHz, CDCl$_3$)

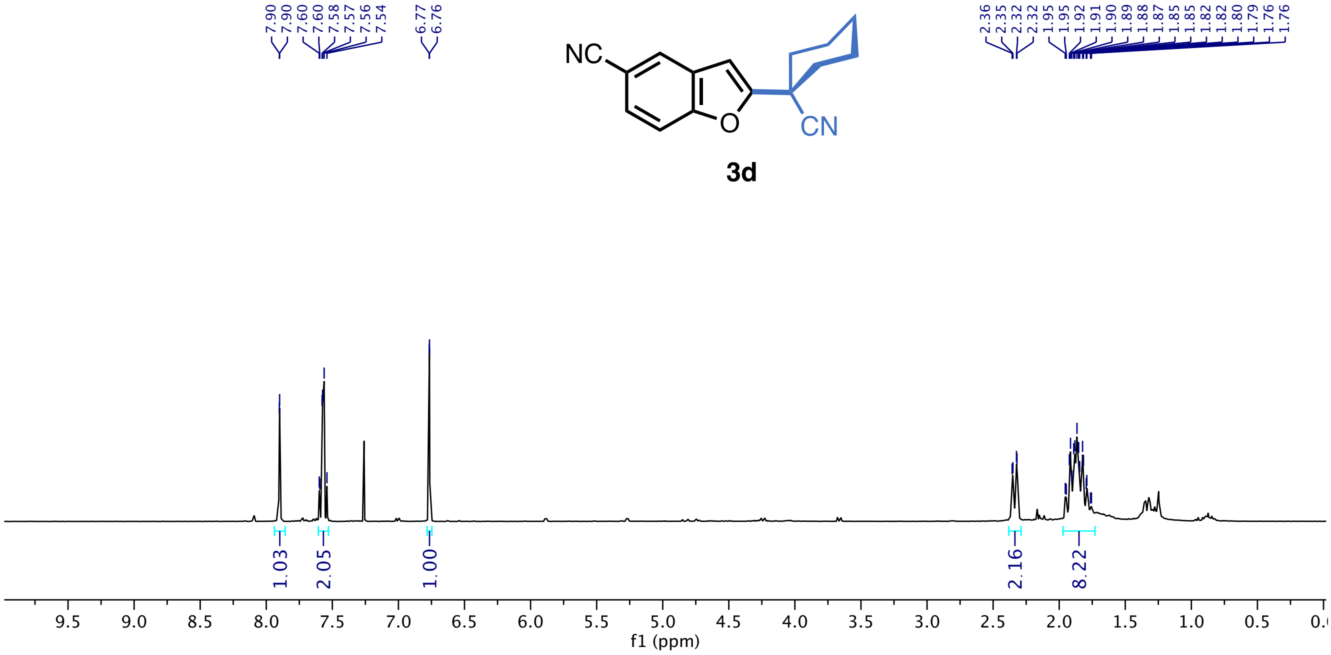

^{13}C NMR (100 MHz, CDCl$_3$)

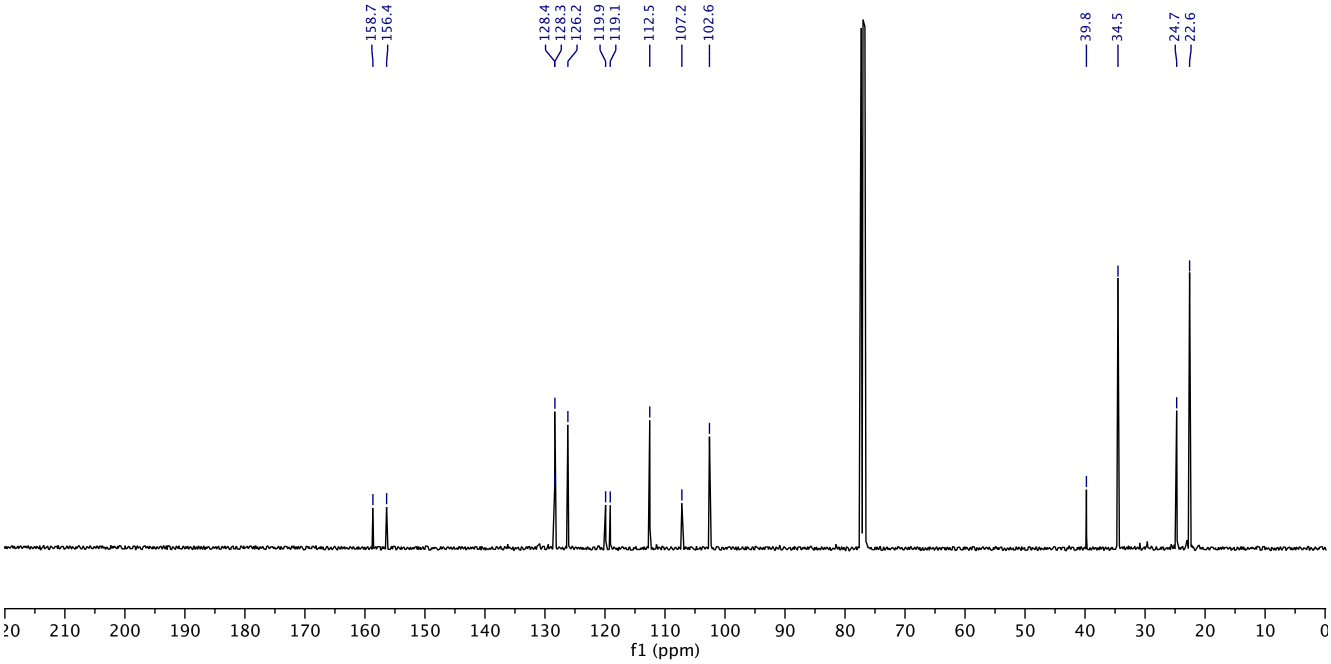

^{1}H NMR (400 MHz, $CDCl_3$)

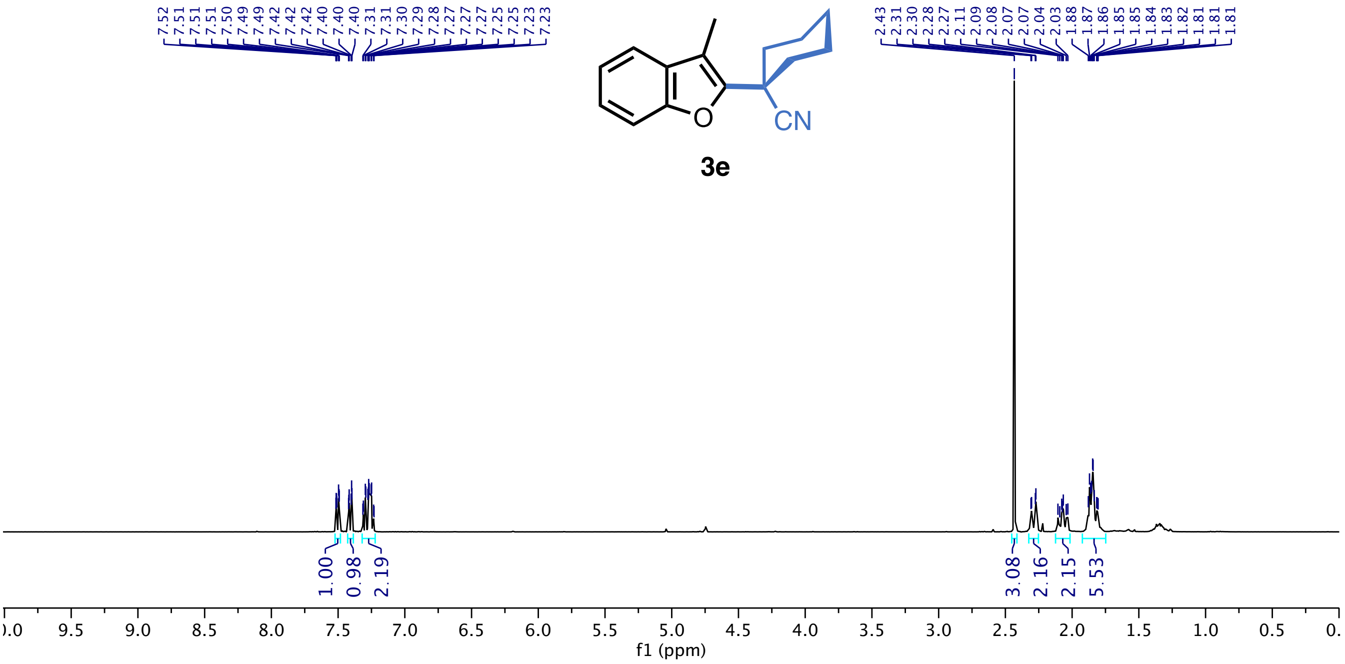

3e

^{13}C NMR (100 MHz, $CDCl_3$)

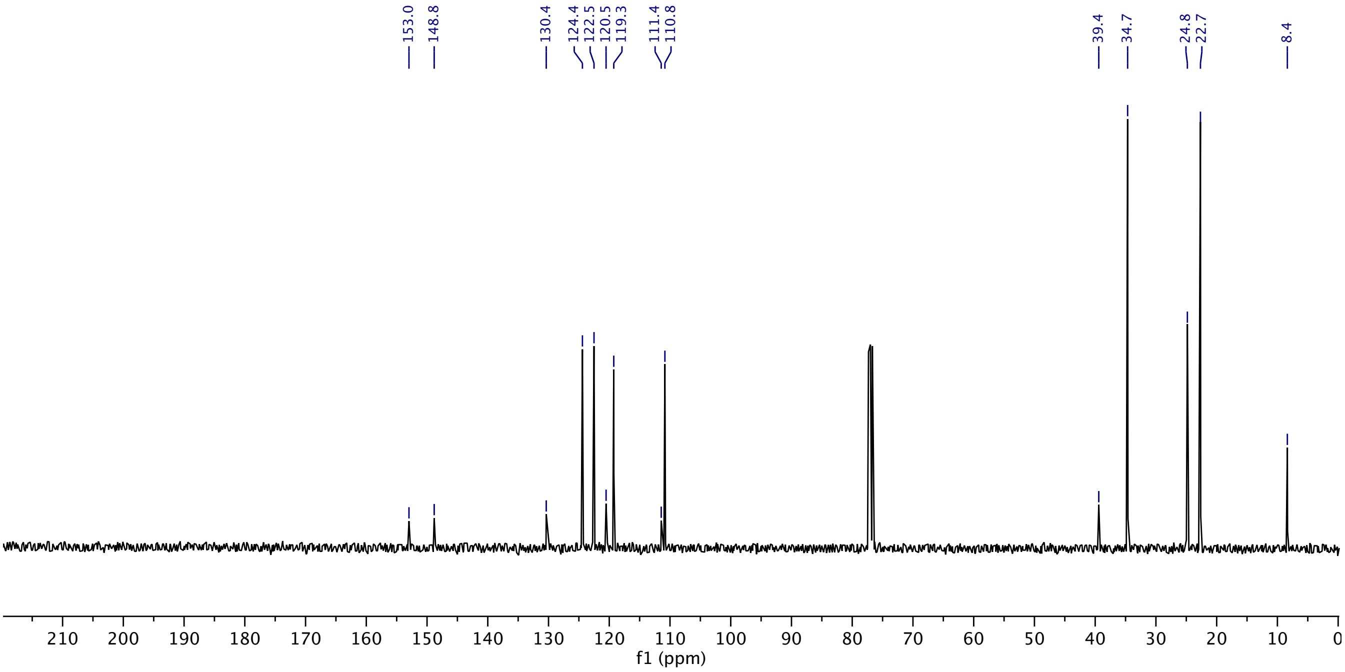

^{1}H NMR (400 MHz, $CDCl_3$)

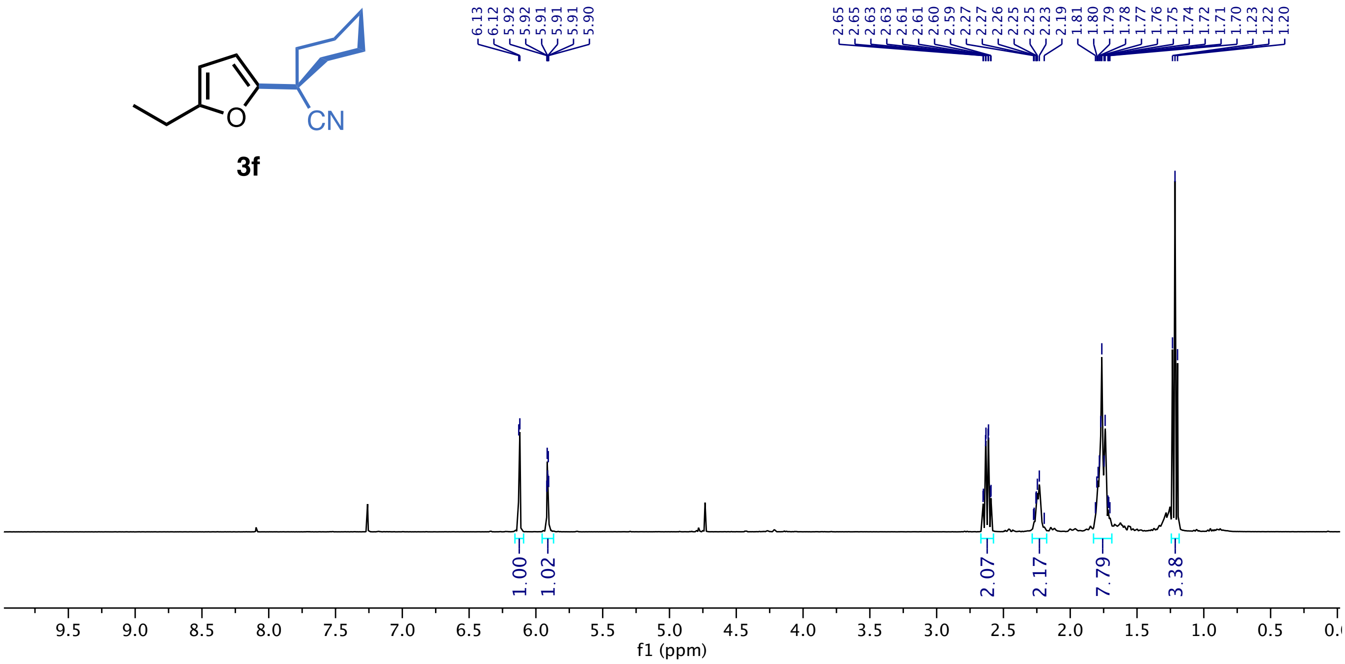

3f

^{13}C NMR (100 MHz, $CDCl_3$)

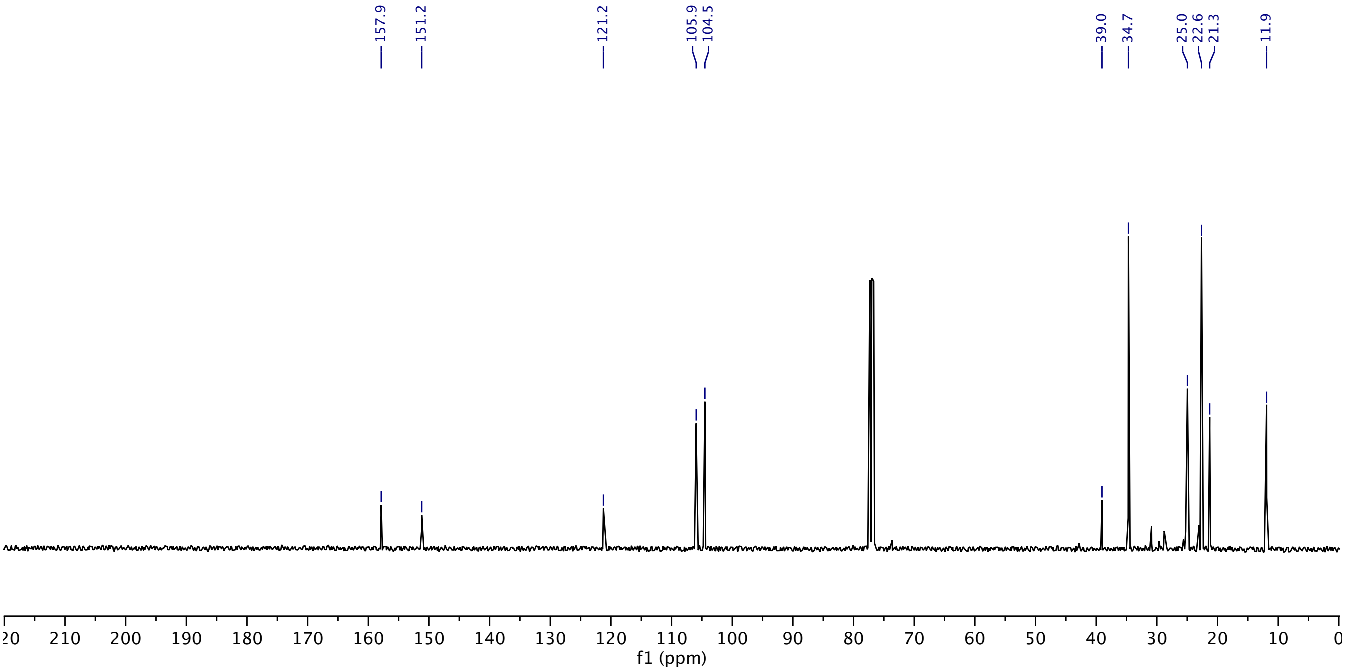

^{1}H NMR (400 MHz, $CDCl_3$)

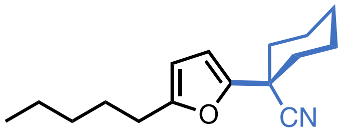

3g

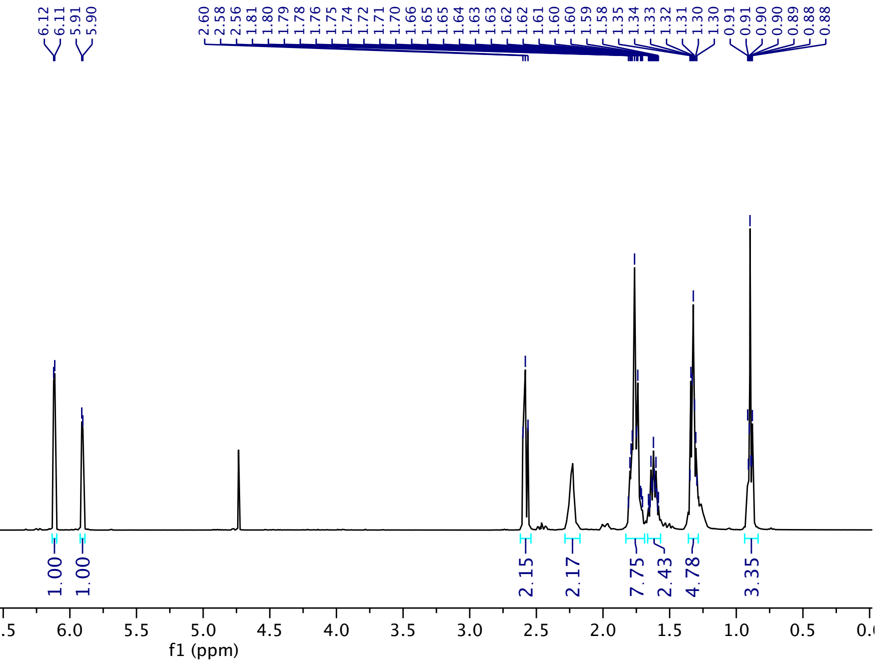

^{13}C NMR (100 MHz, $CDCl_3$)

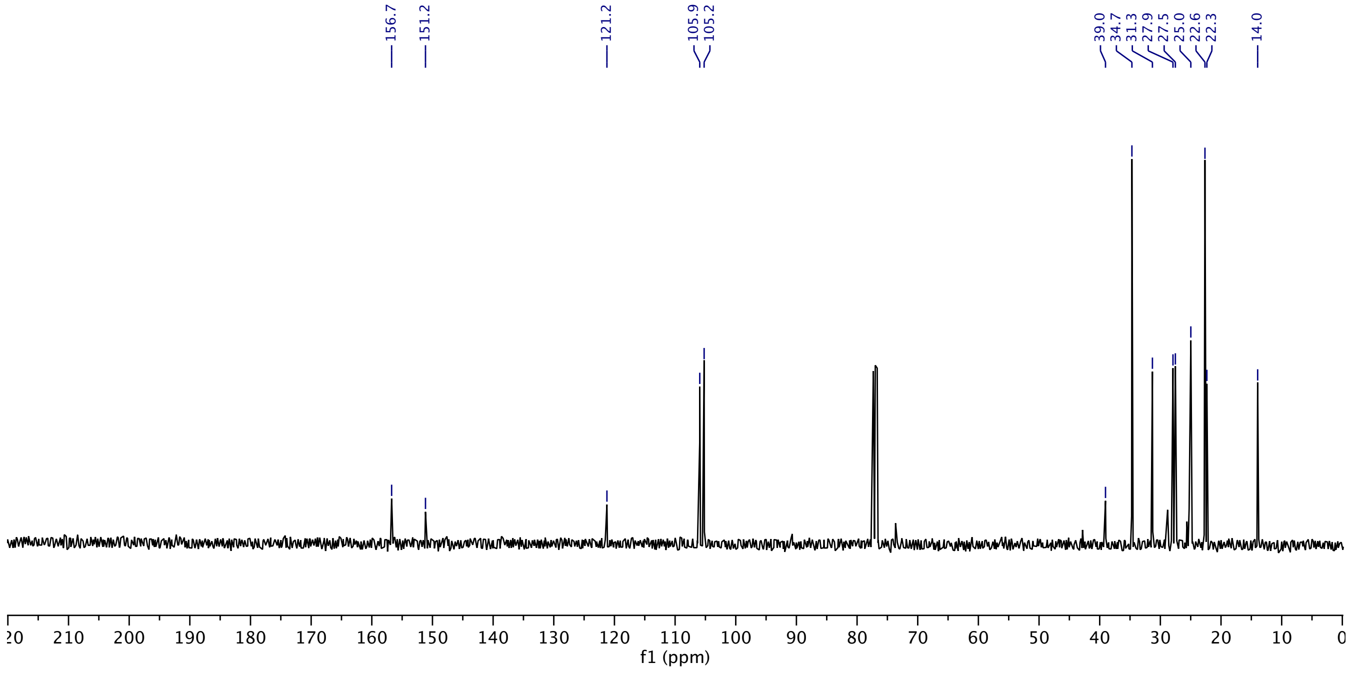

^{1}H NMR (400 MHz, $CDCl_3$)

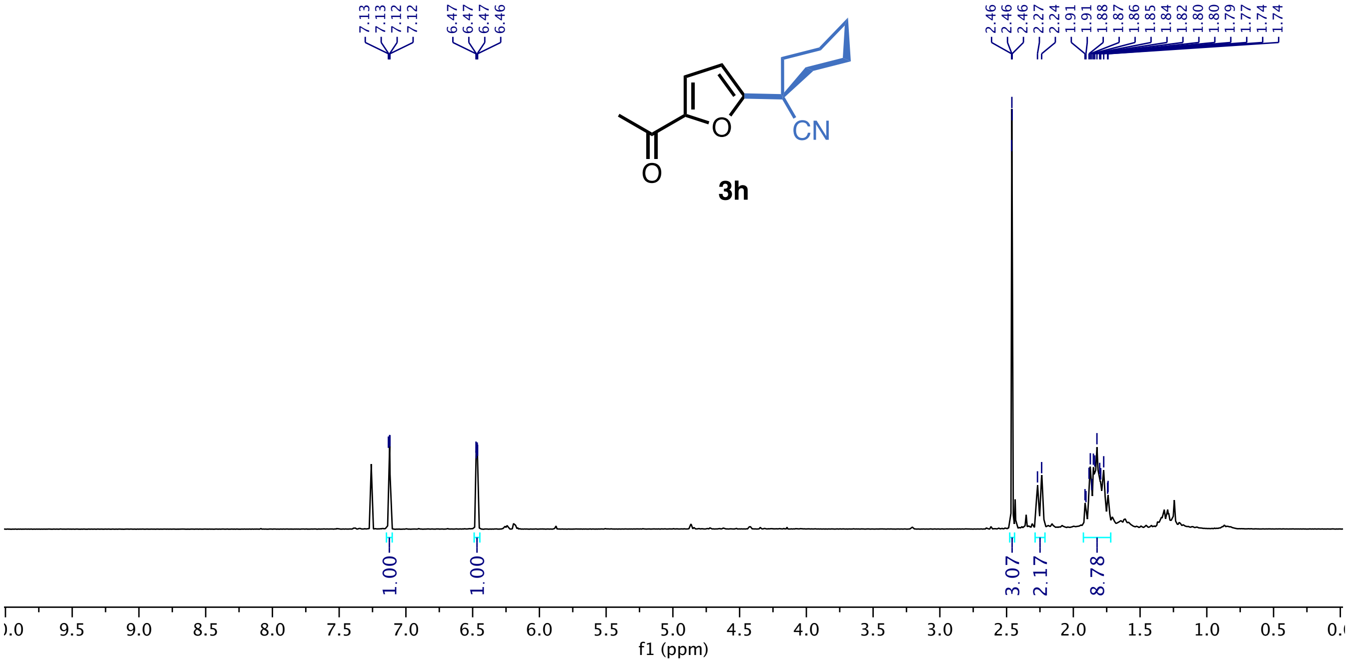

^{13}C NMR (100 MHz, $CDCl_3$)

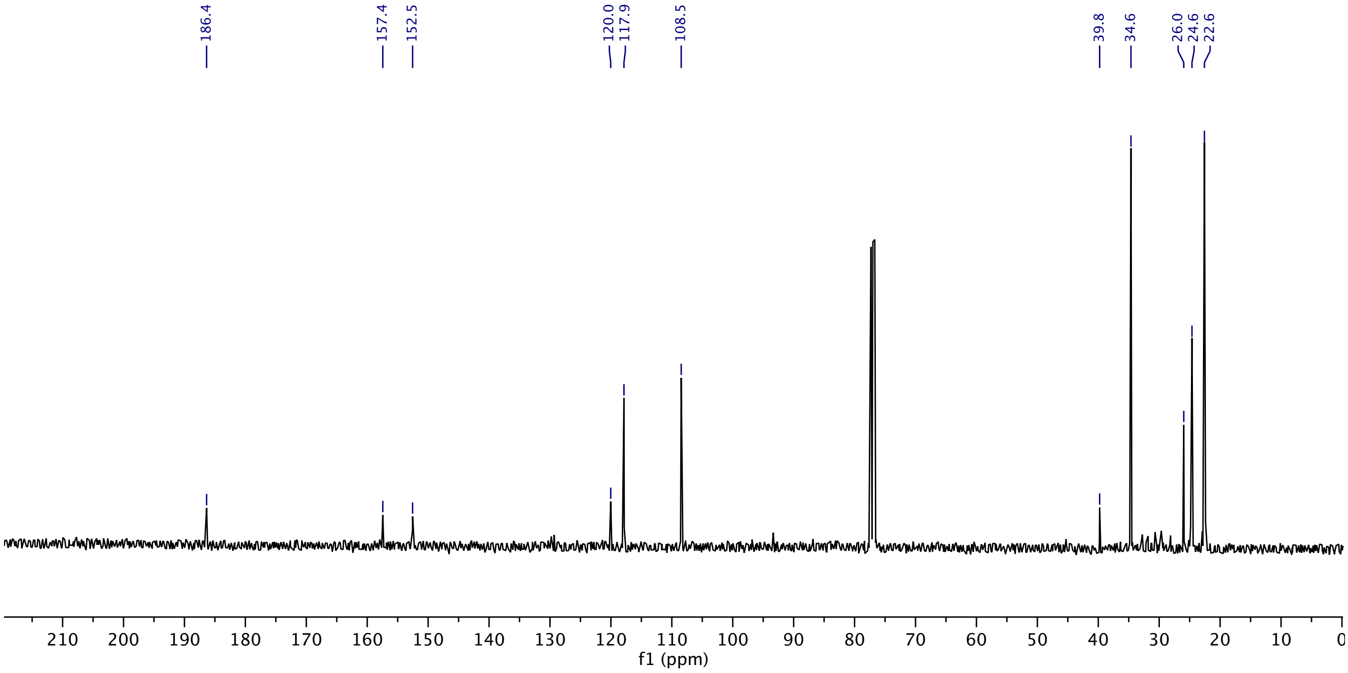

^{1}H NMR (400 MHz, $CDCl_3$)

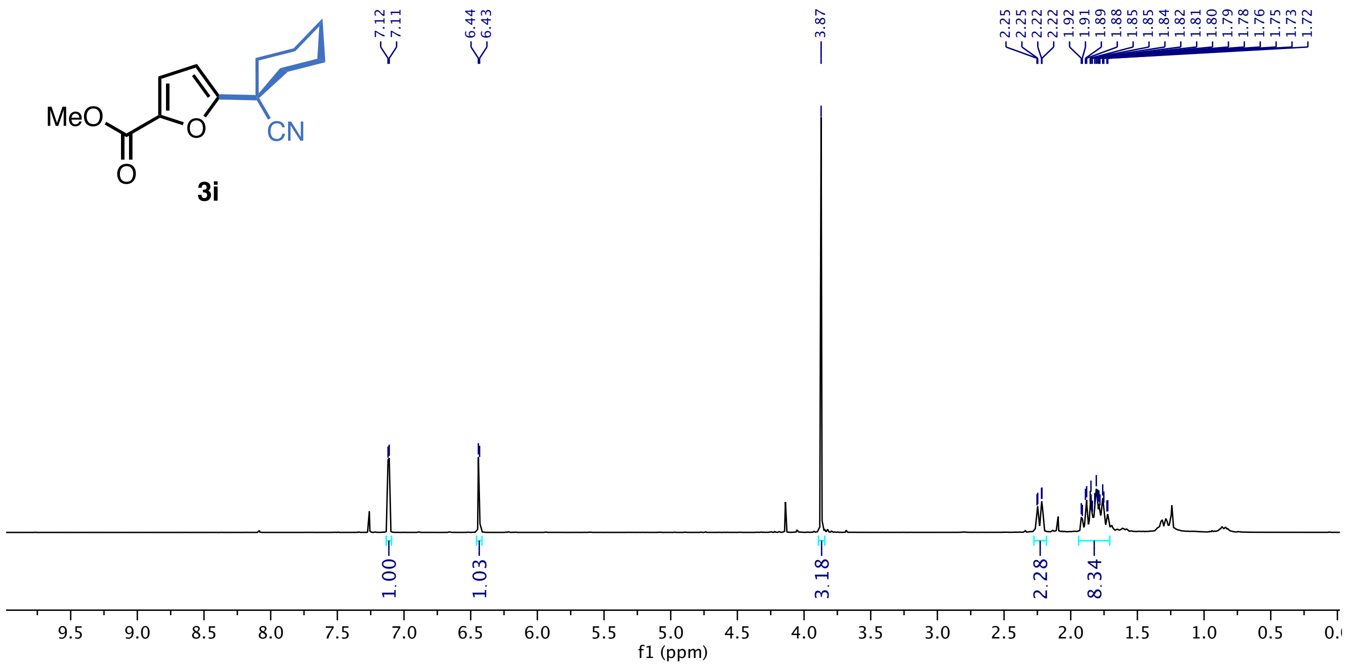

^{13}C NMR (100 MHz, $CDCl_3$)

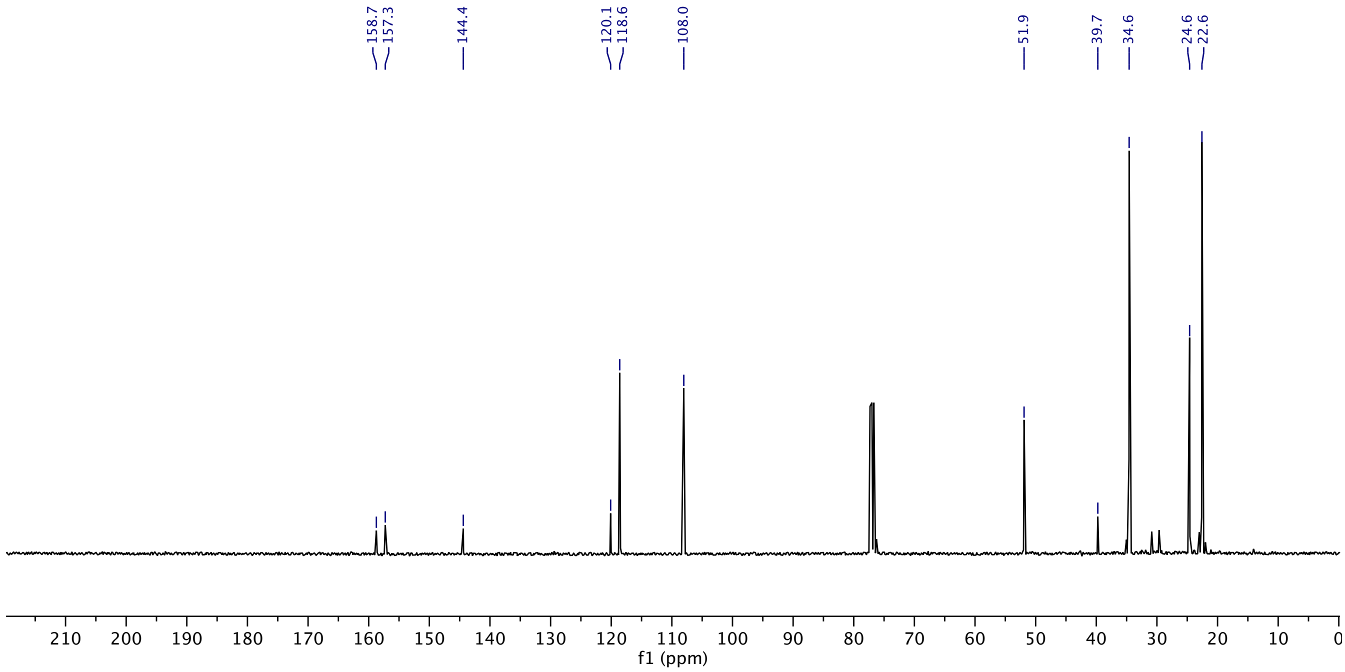

^{1}H NMR (400 MHz, $CDCl_3$)

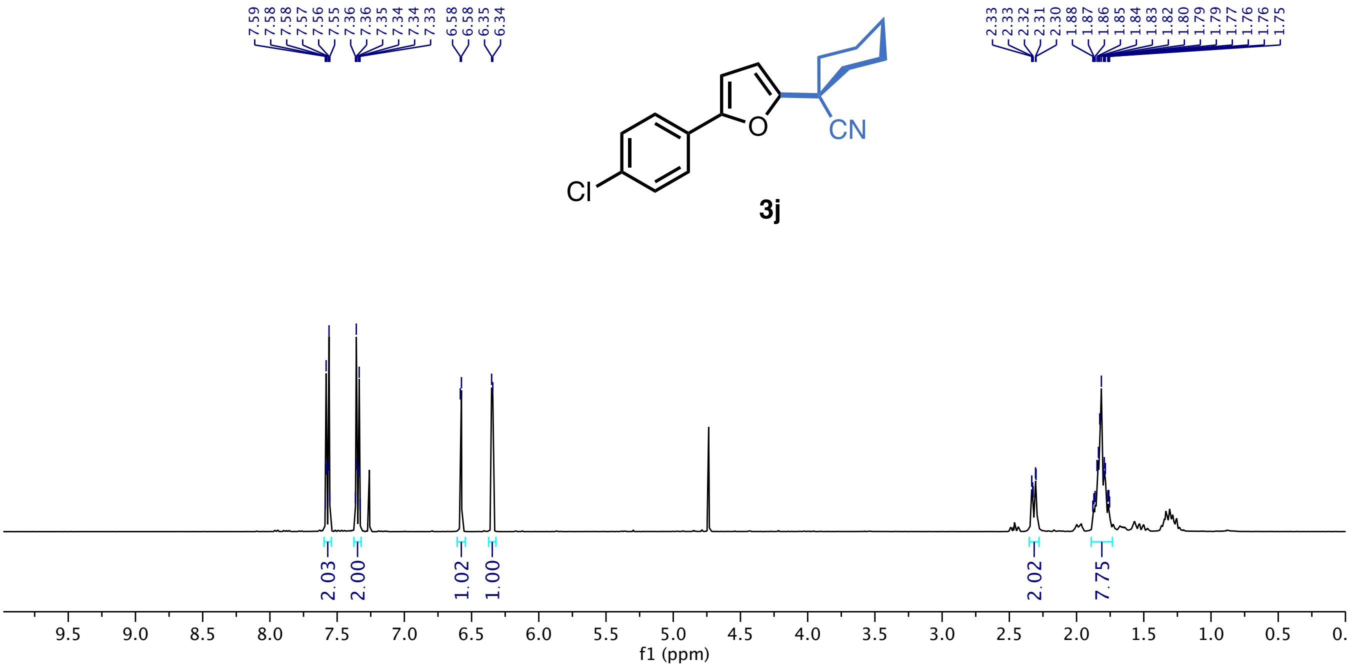

^{13}C NMR (100 MHz, $CDCl_3$)

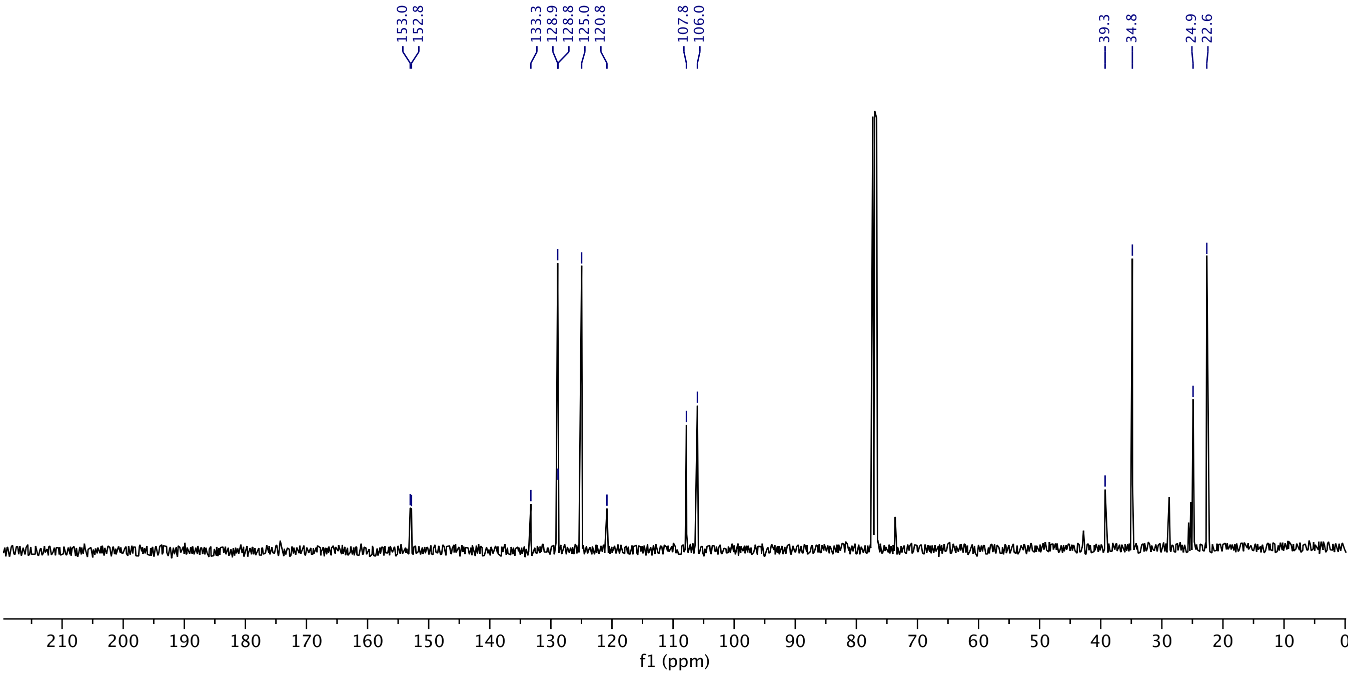

^{1}H NMR (400 MHz, $CDCl_3$)

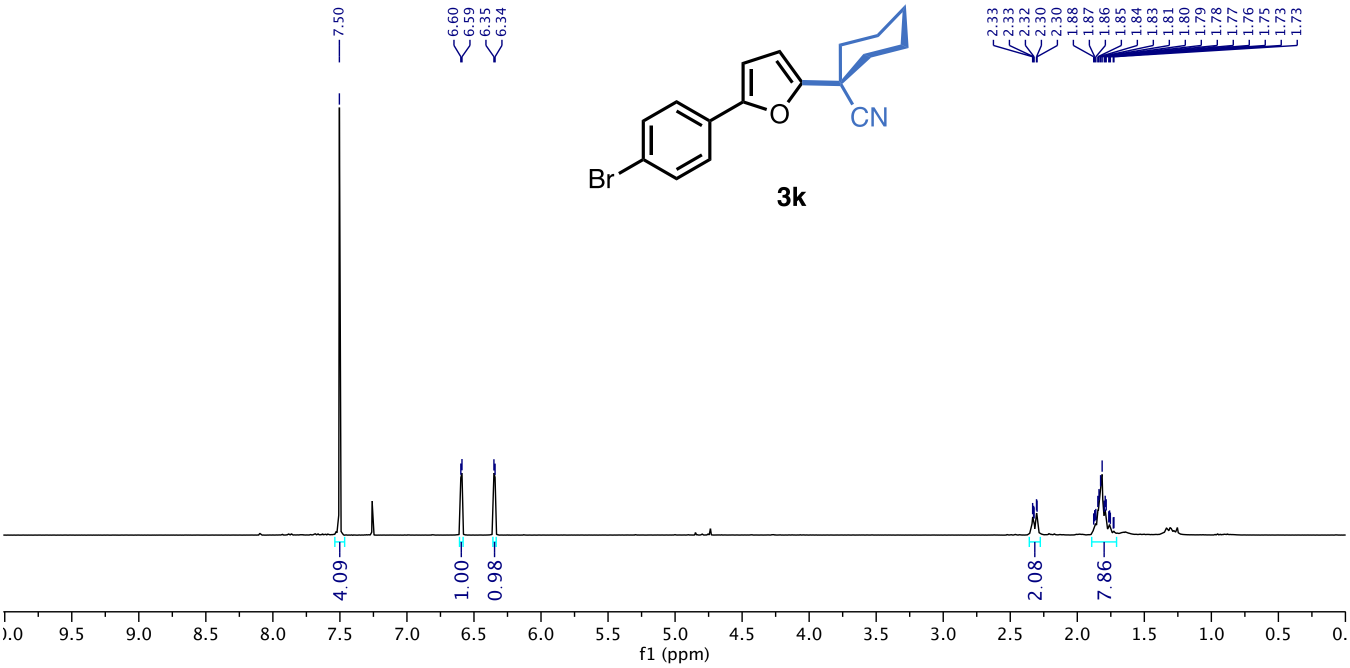

^{13}C NMR (100 MHz, $CDCl_3$)

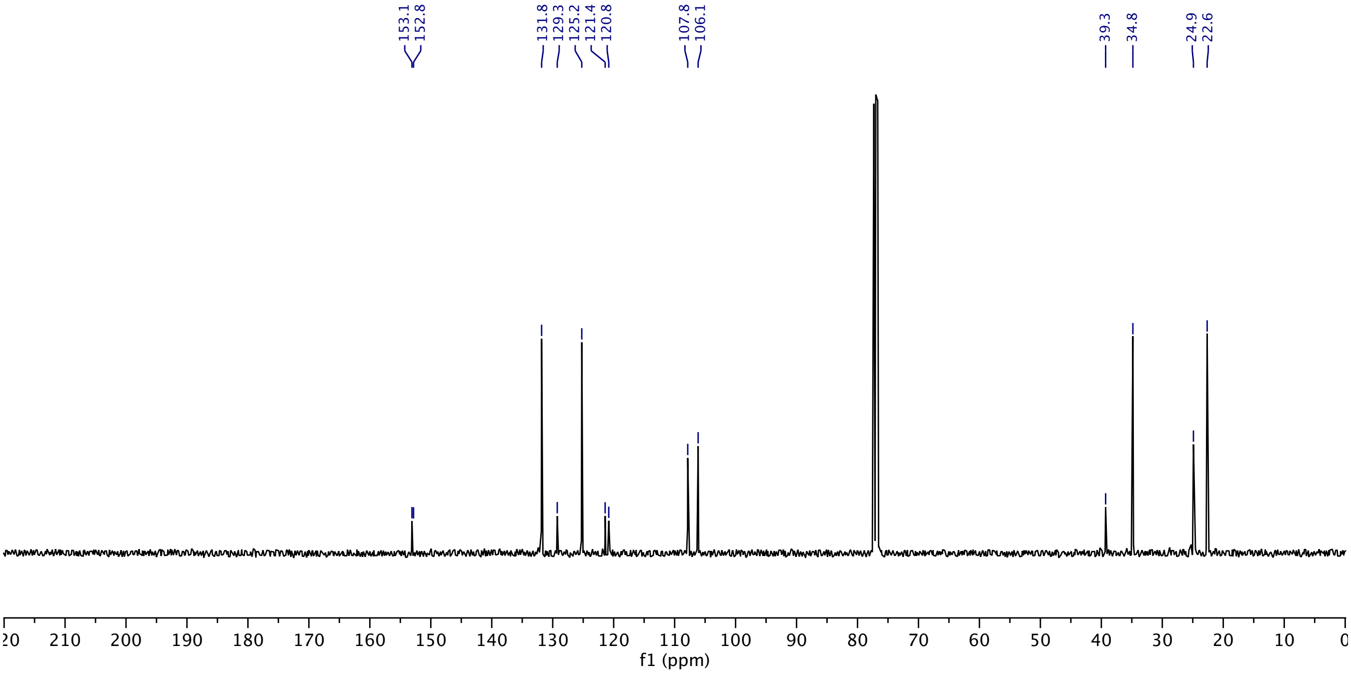

^{1}H NMR (400 MHz, $CDCl_3$)

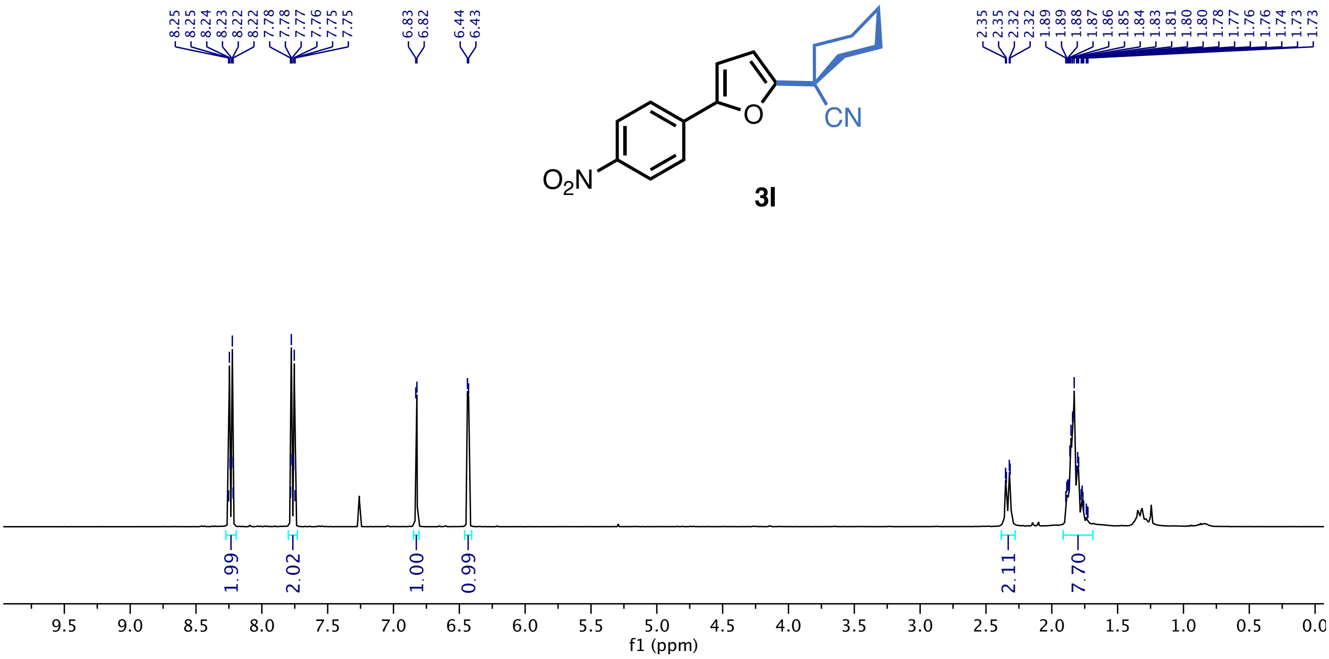

^{13}C NMR (100 MHz, $CDCl_3$)

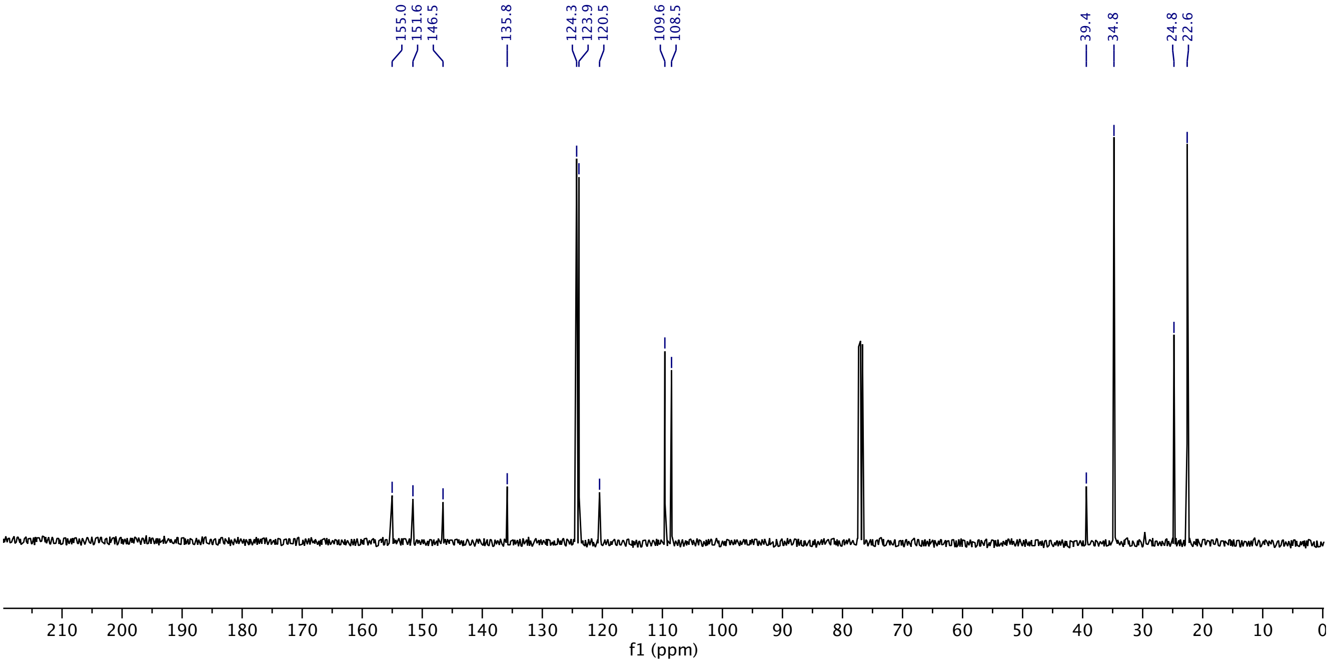

^1H NMR (400 MHz, $CDCl_3$)

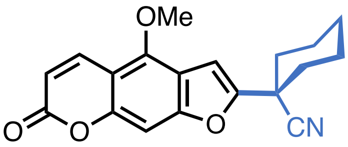

3m

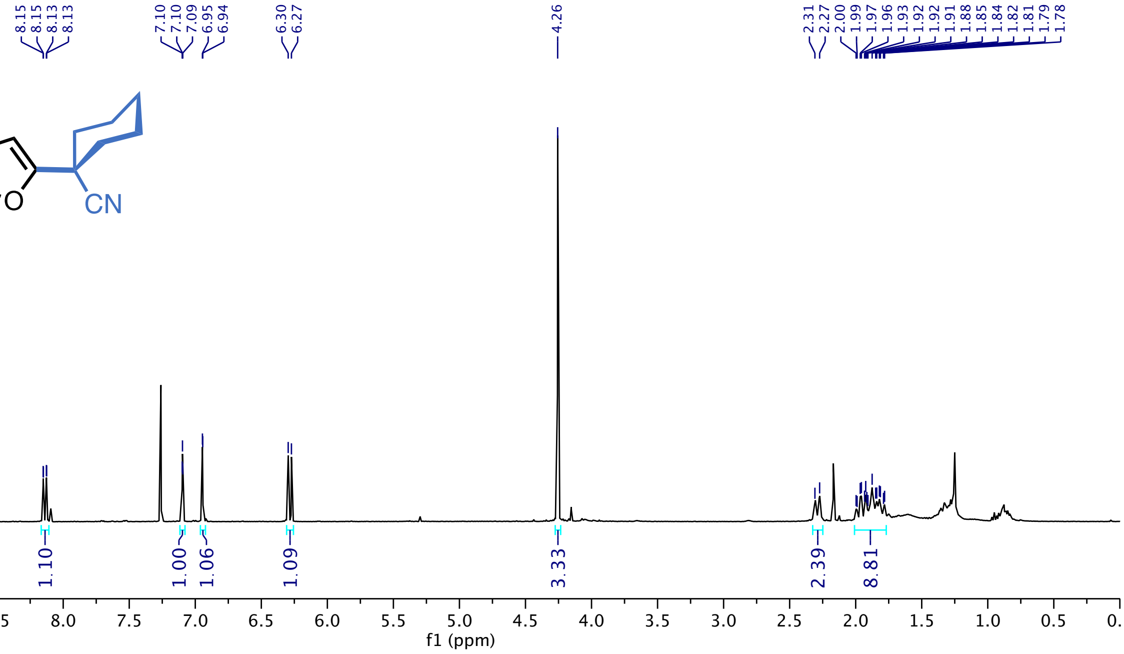

^{13}C NMR (100 MHz, $CDCl_3$)

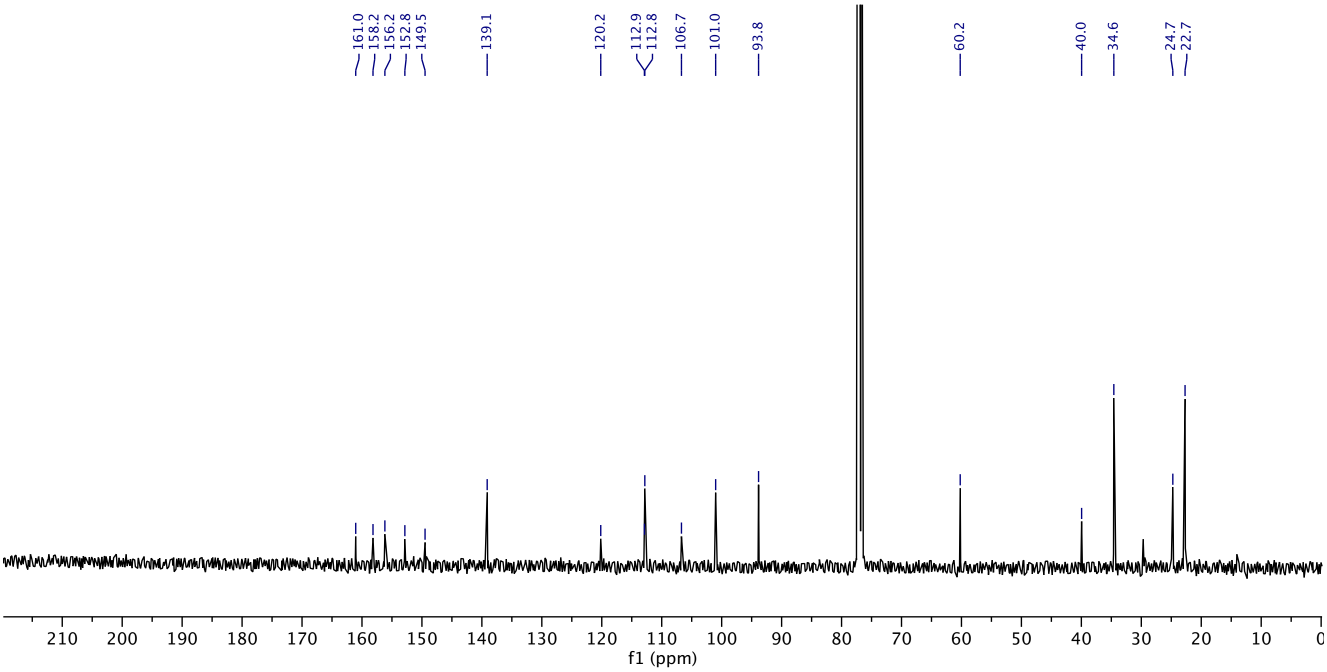

^{1}H NMR (500 MHz, $CDCl_3$)

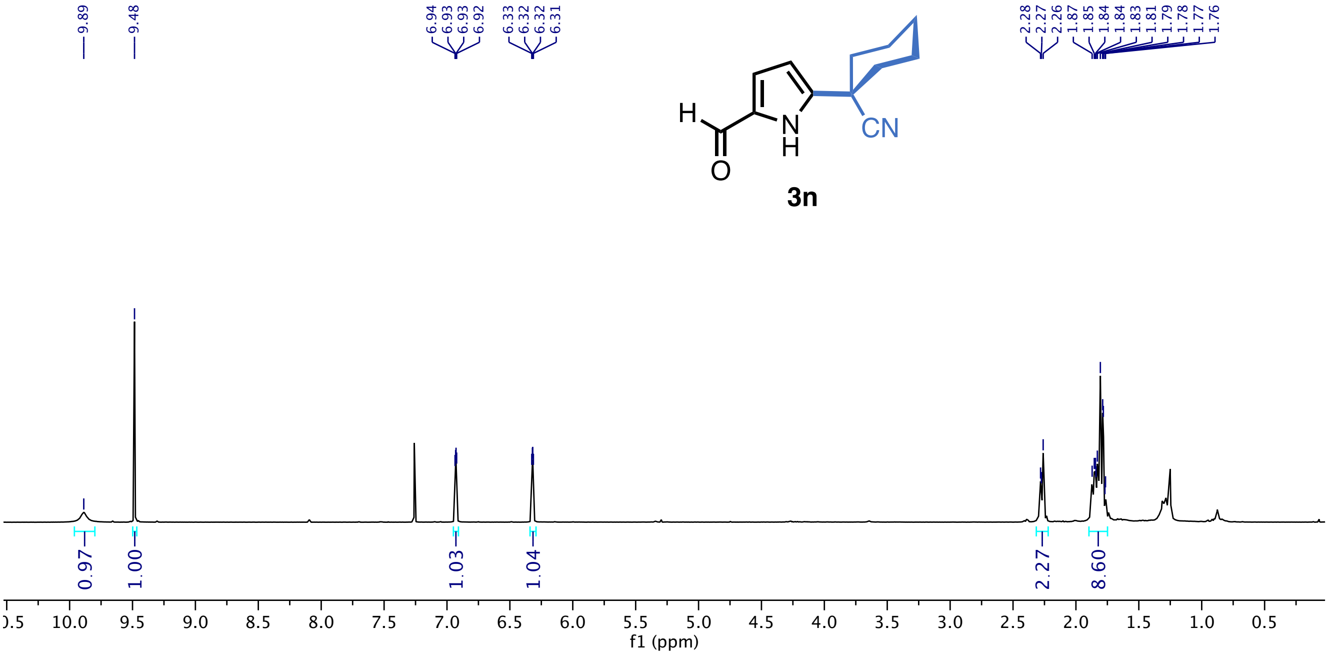

^{13}C NMR (125 MHz, $CDCl_3$)

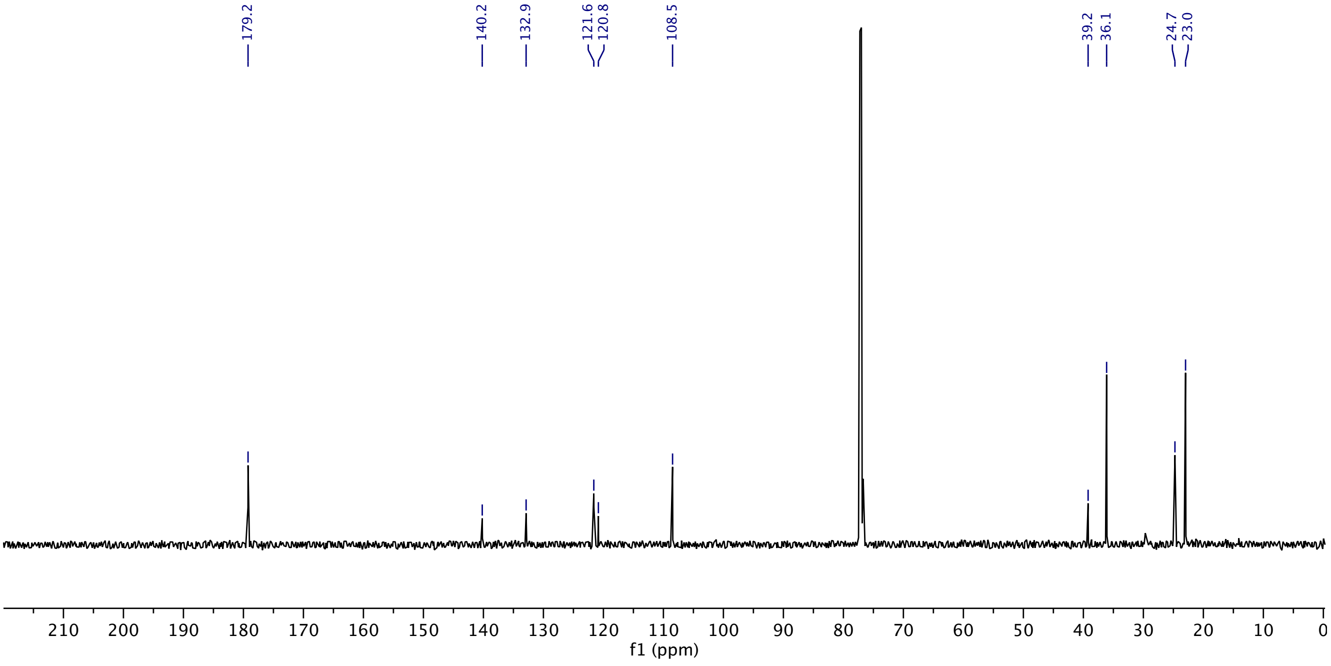

^{1}H NMR (500 MHz, $CDCl_3$)

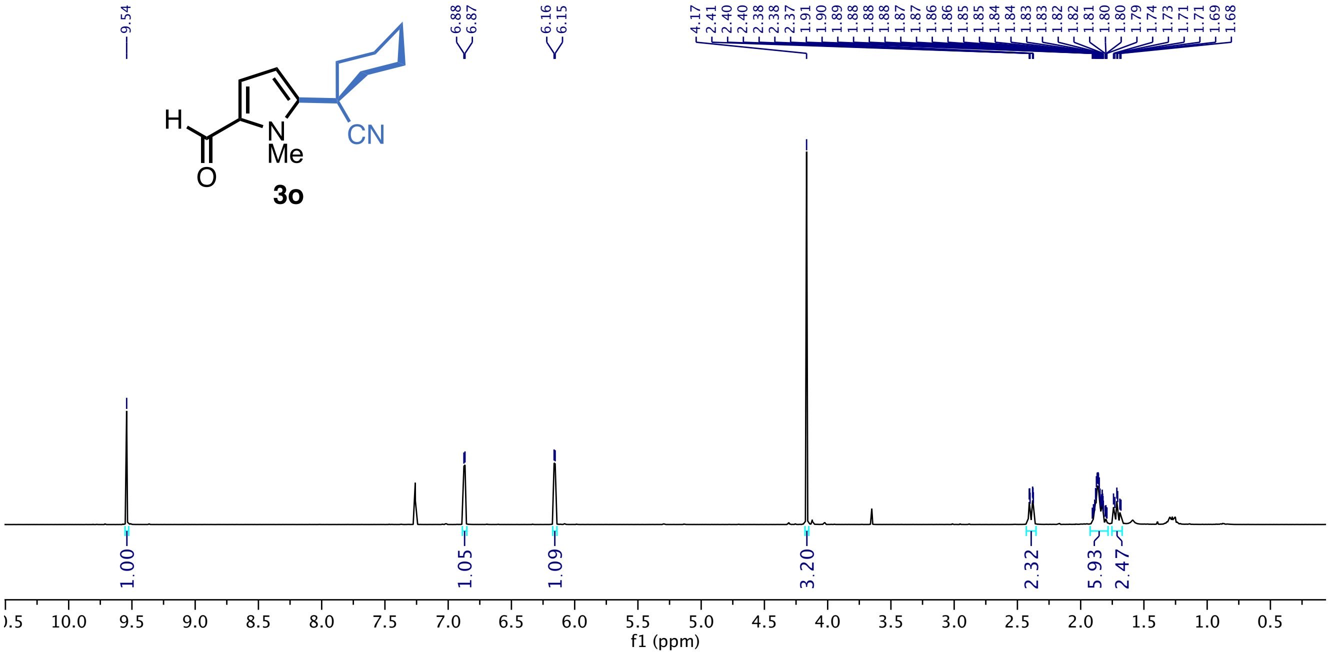

^{13}C NMR (125 MHz, $CDCl_3$)

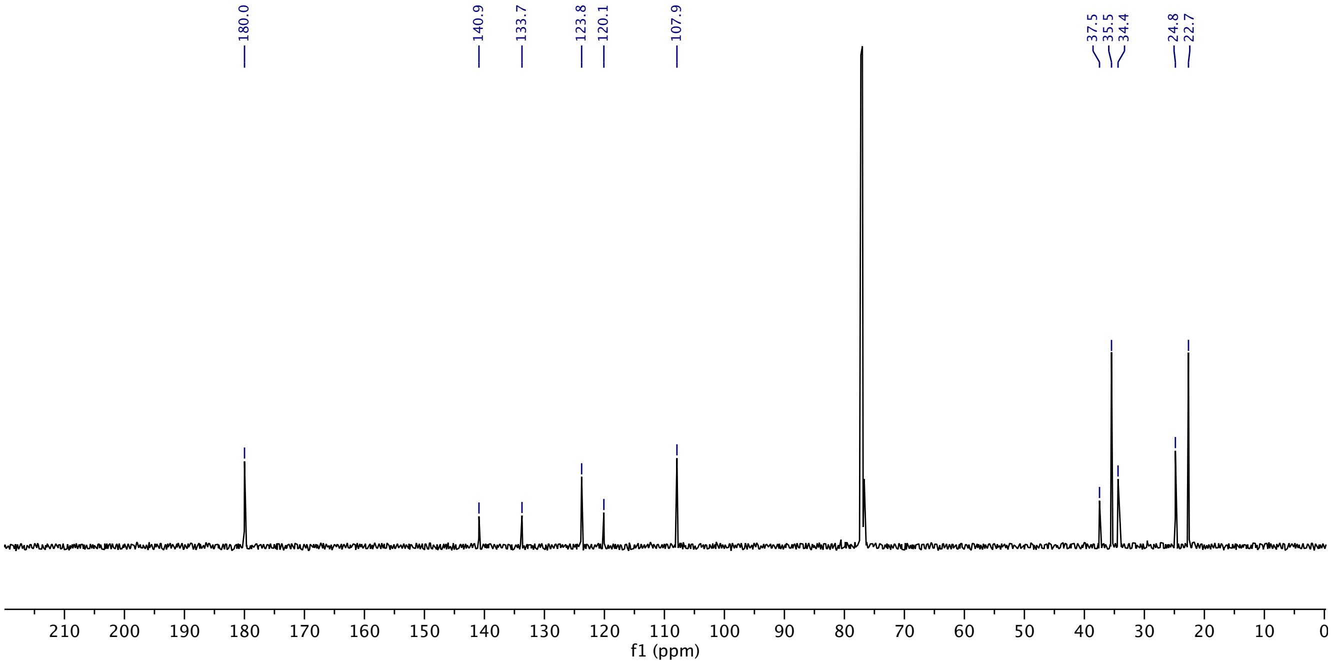

^{1}H NMR (500 MHz, $CDCl_3$)

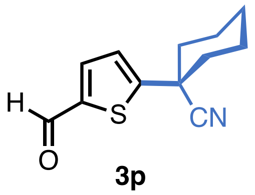

3p

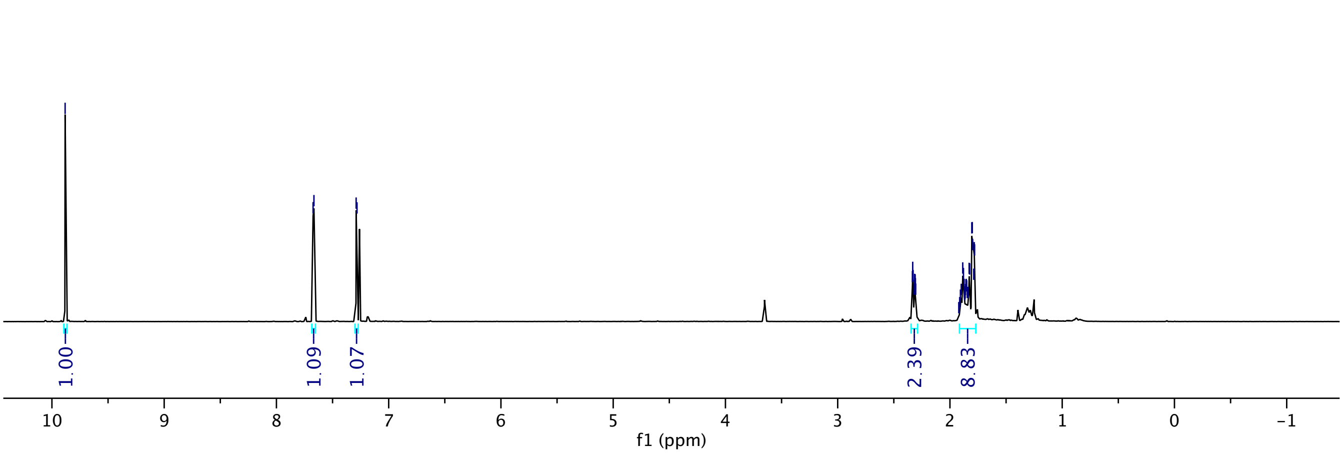

^{13}C NMR (125 MHz, $CDCl_3$)

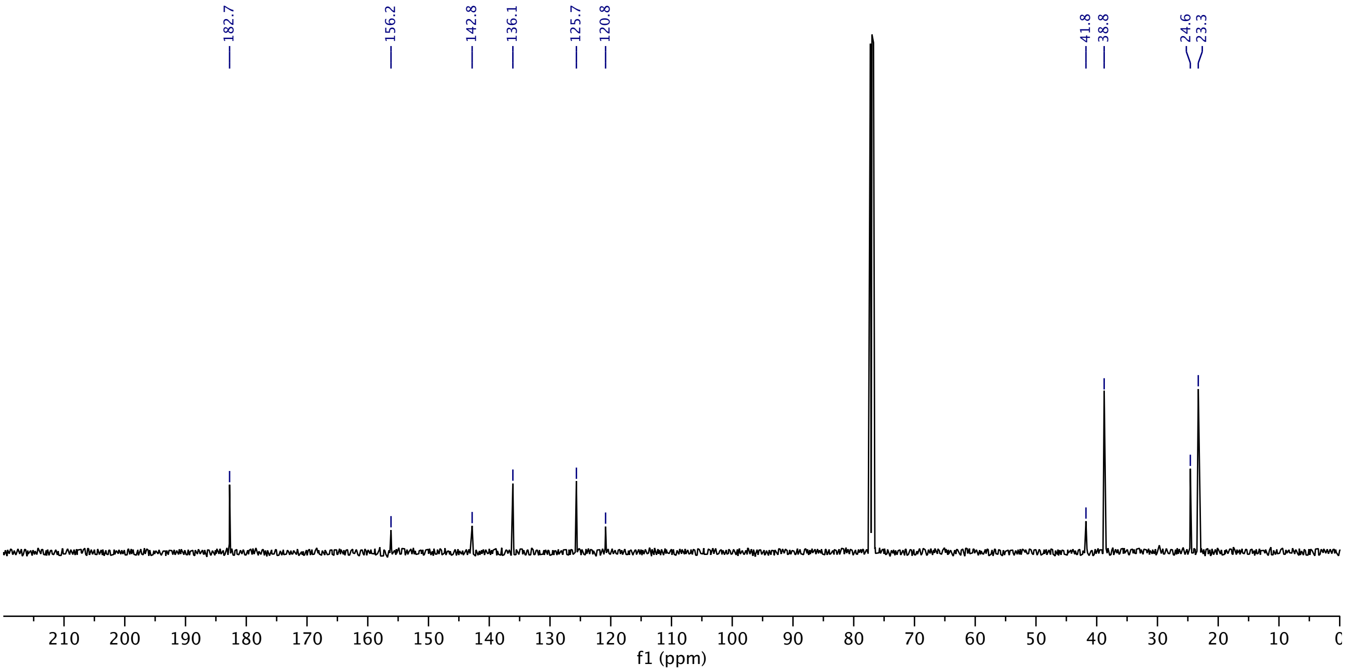

^{1}H NMR (400 MHz, $CDCl_3$)

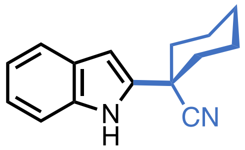

3q

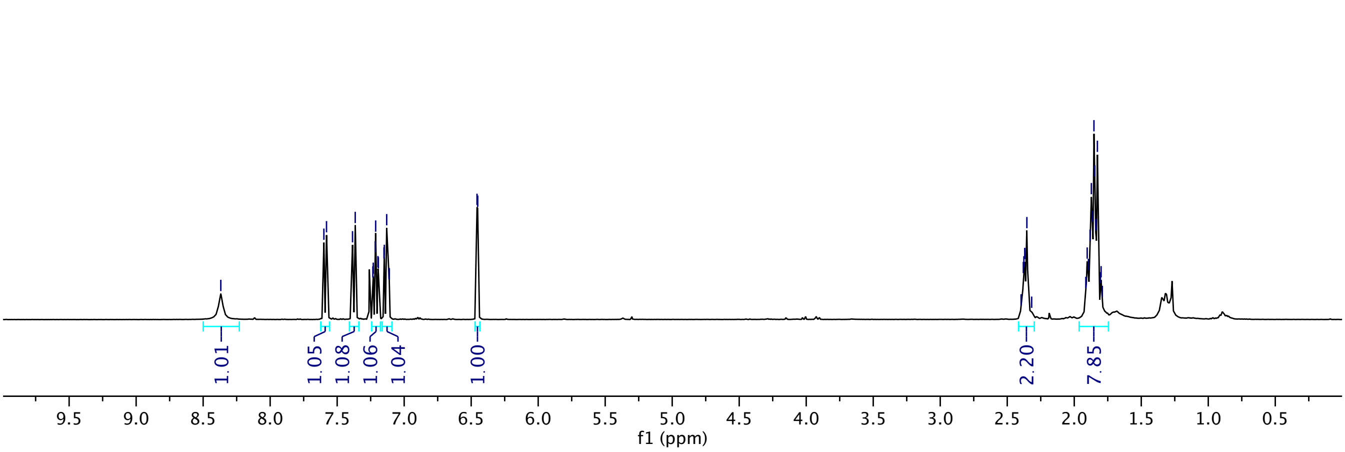

^{13}C NMR (100 MHz, $CDCl_3$)

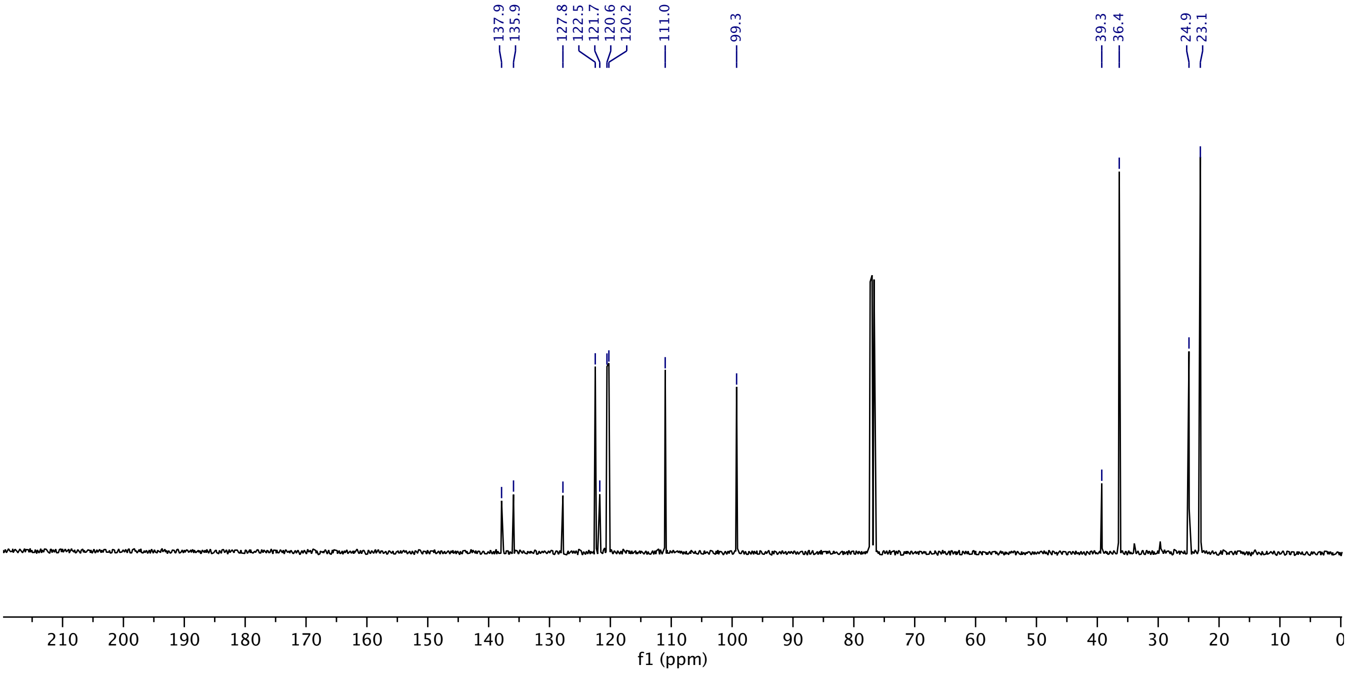

^{1}H NMR (400 MHz, $CDCl_3$)

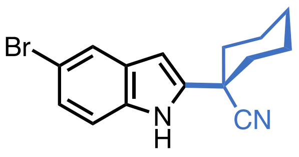

3r

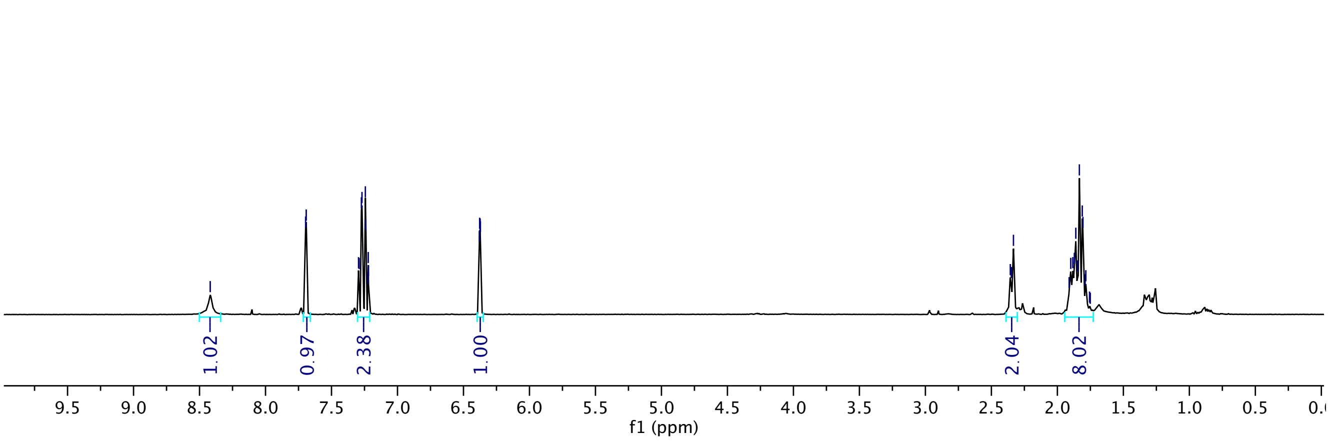

^{13}C NMR (100 MHz, $CDCl_3$)

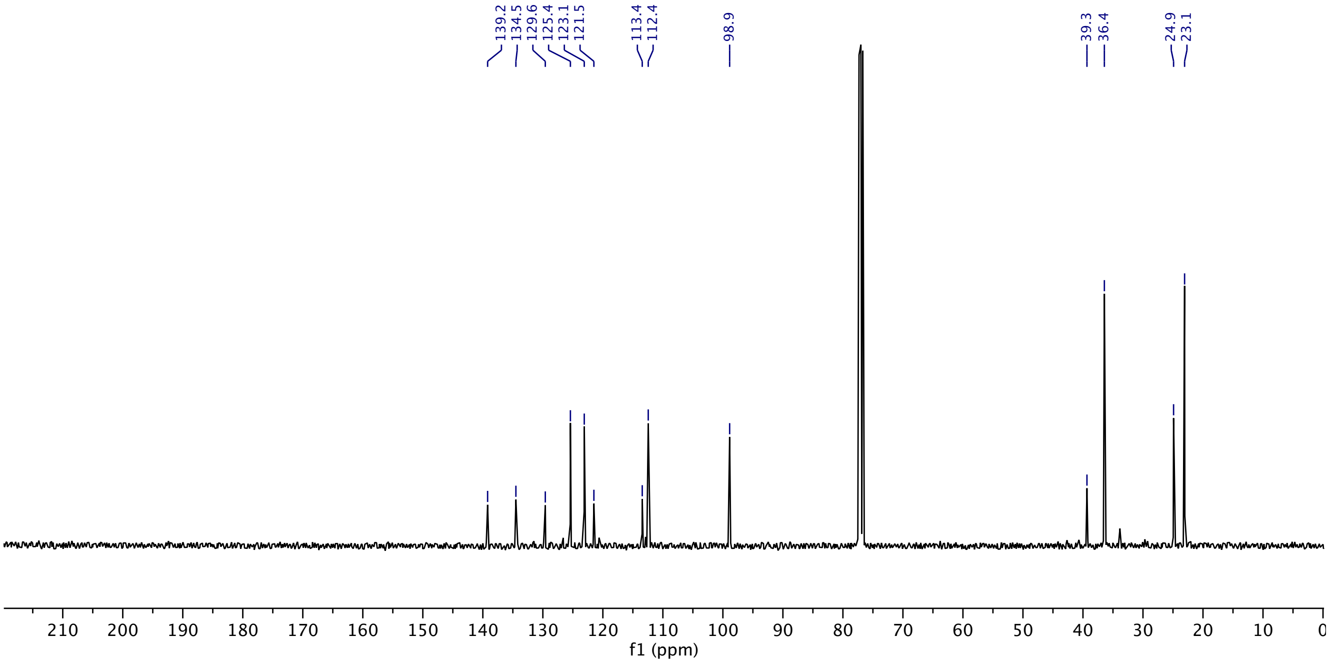

^{1}H NMR (400 MHz, $CDCl_3$)

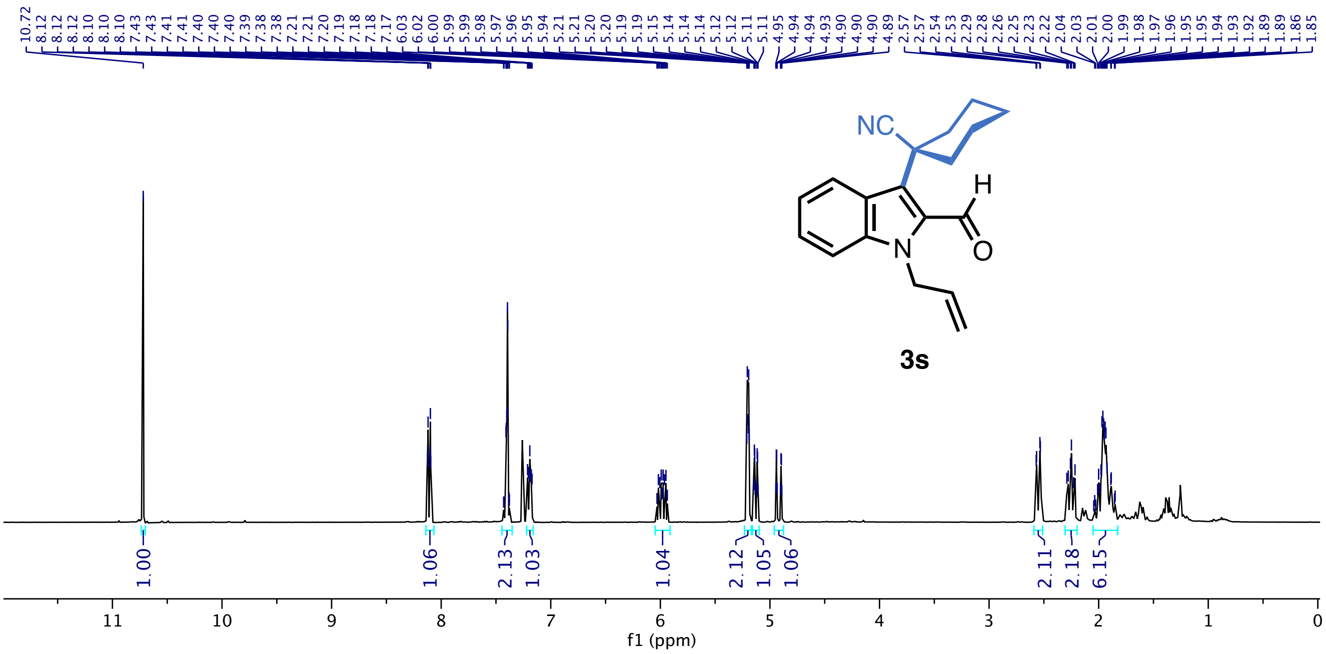

^{13}C NMR (100 MHz, $CDCl_3$)

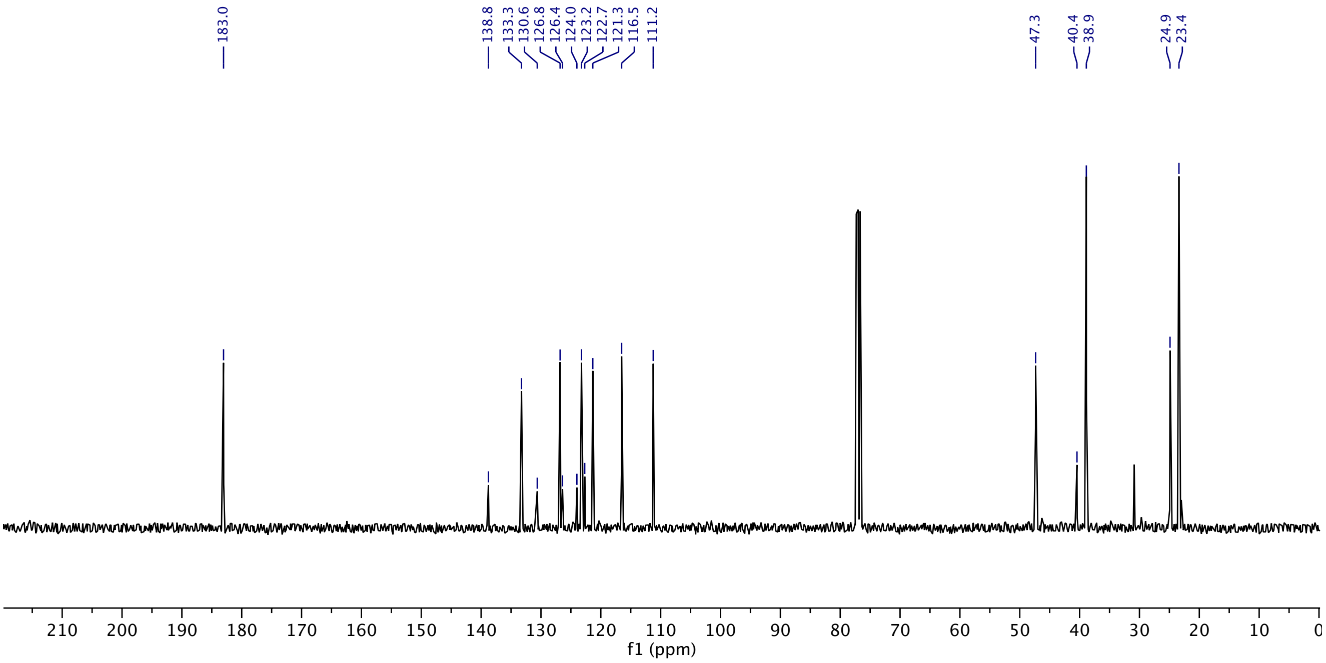

^{1}H NMR (400 MHz, $CDCl_3$)

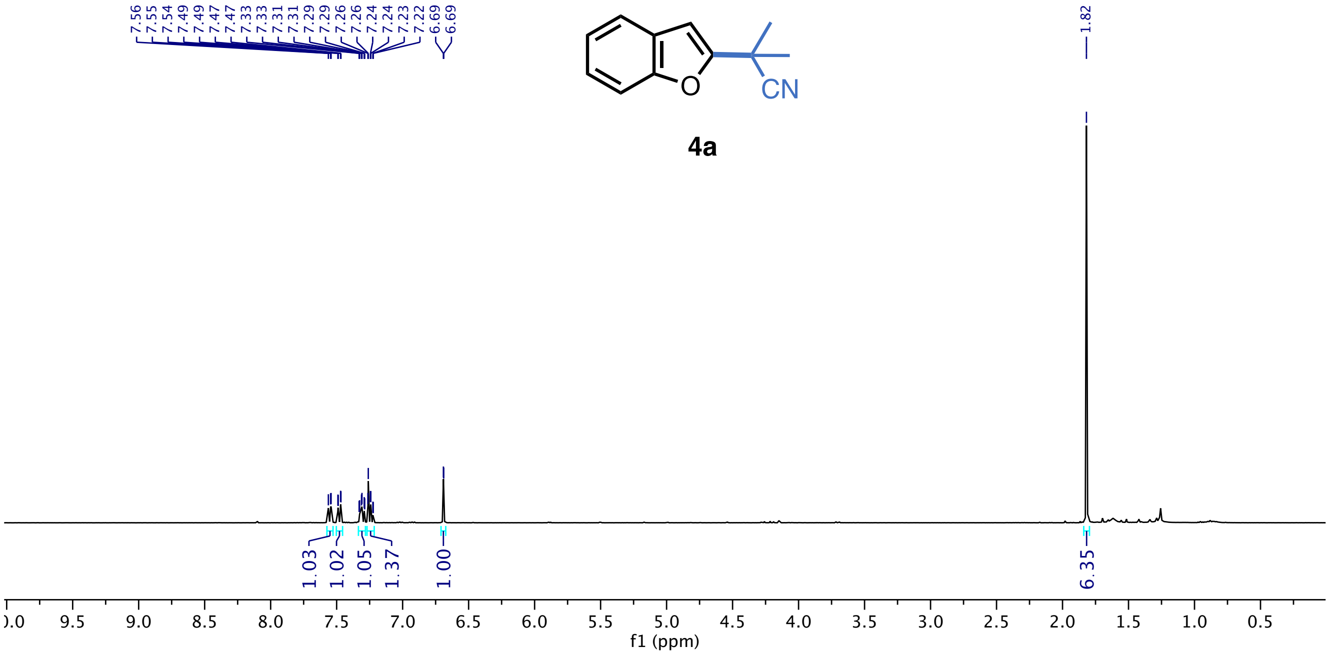

^{13}C NMR (100 MHz, $CDCl_3$)

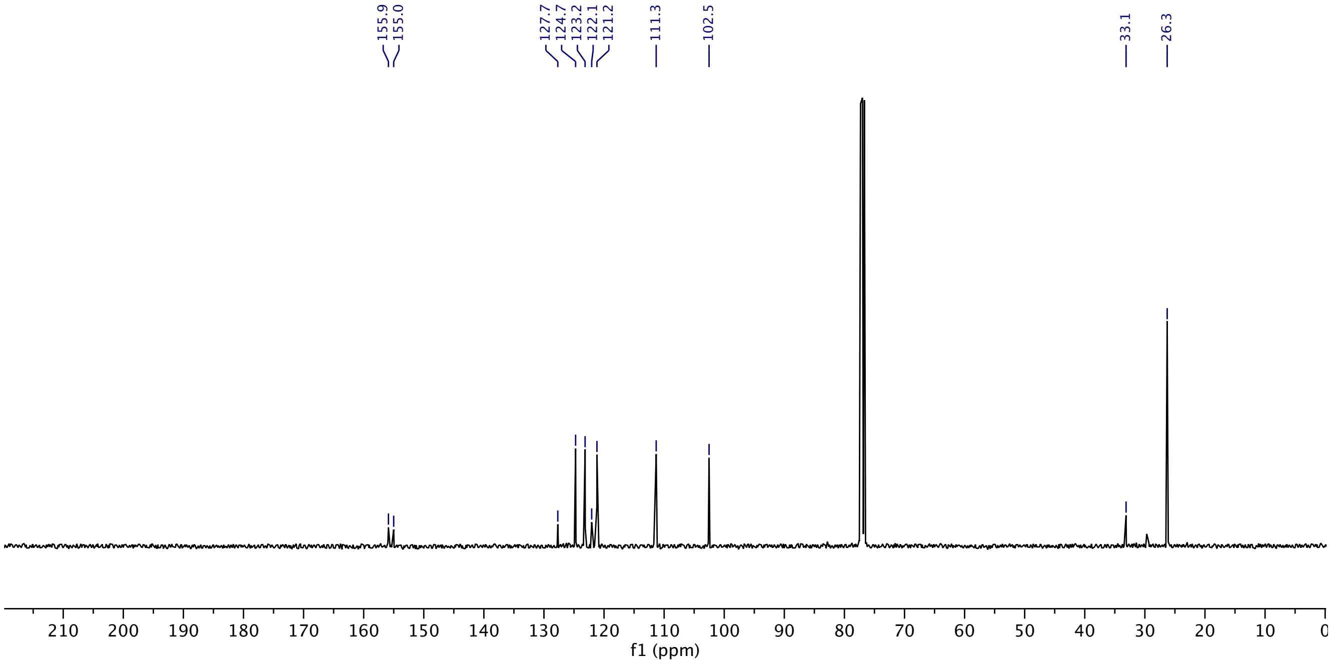

^{1}H NMR (400 MHz, $CDCl_3$)

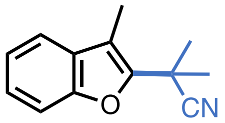
4b

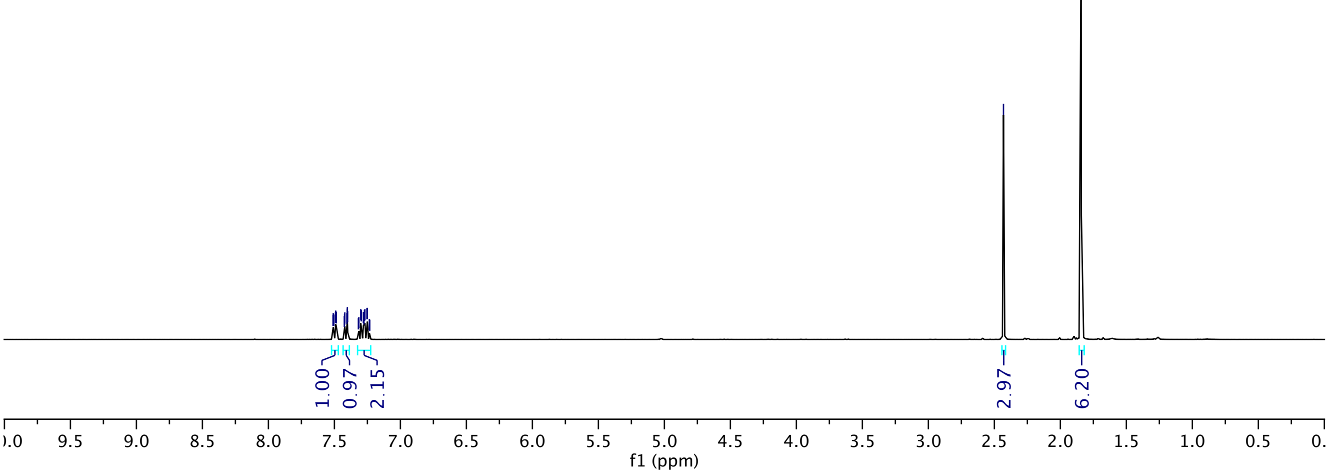

^{13}C NMR (100 MHz, $CDCl_3$)

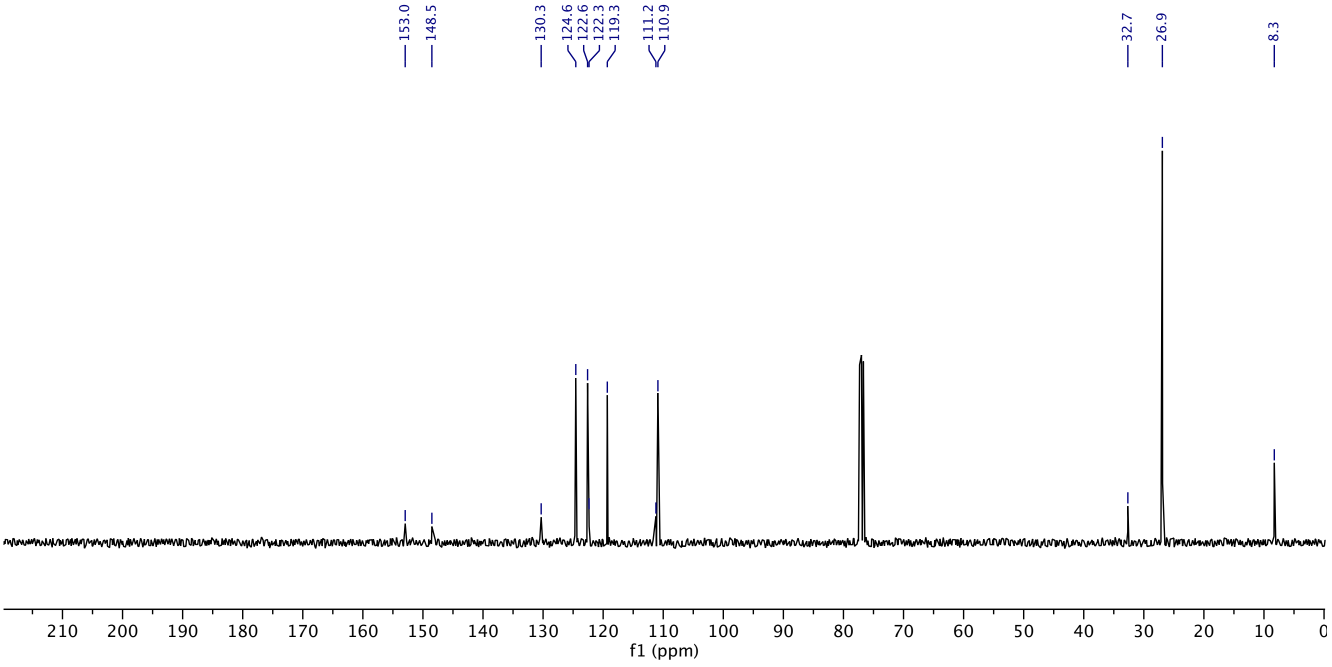

^{1}H NMR (400 MHz, $CDCl_3$)

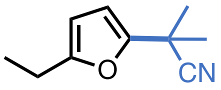

4c

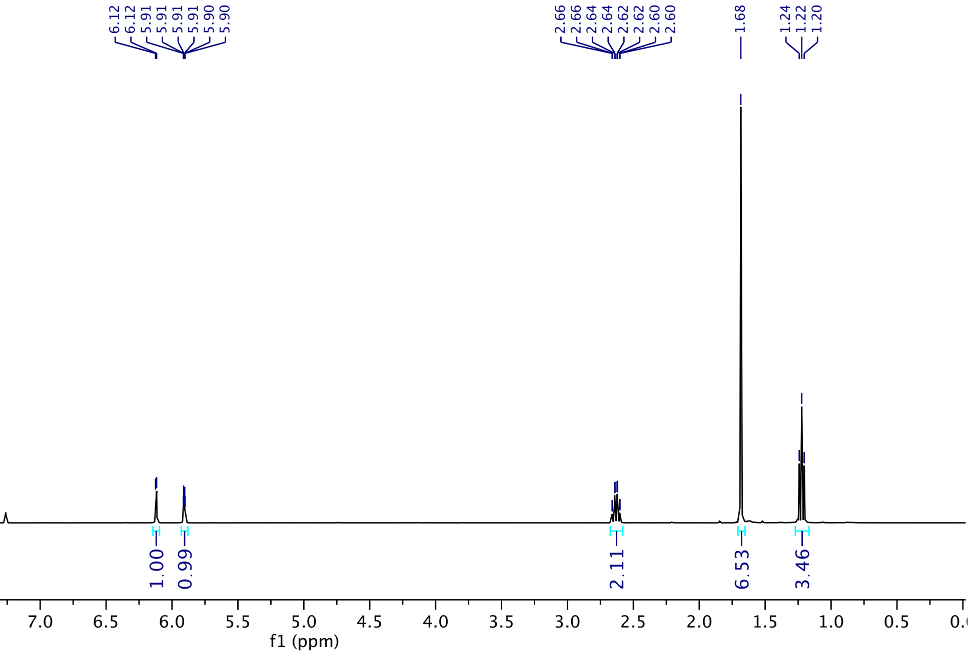

^{13}C NMR (100 MHz, $CDCl_3$)

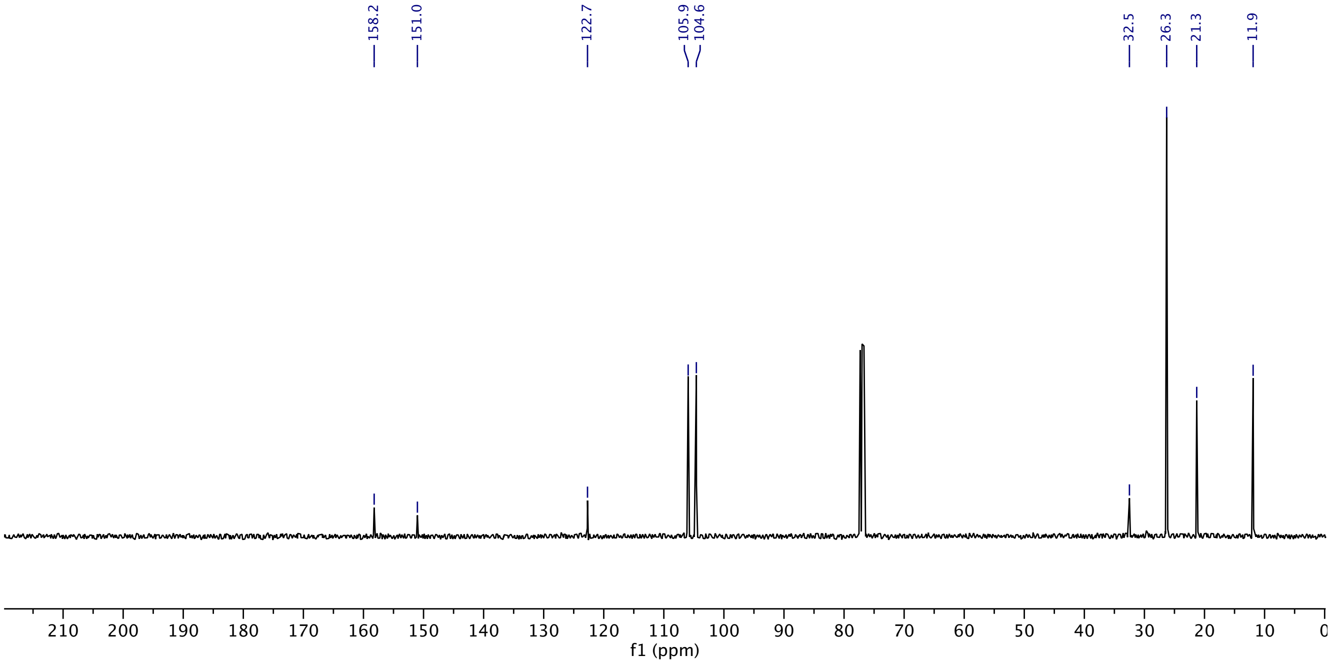

^1H NMR (400 MHz, $CDCl_3$)

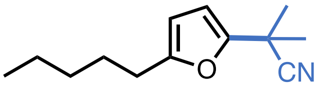

4d

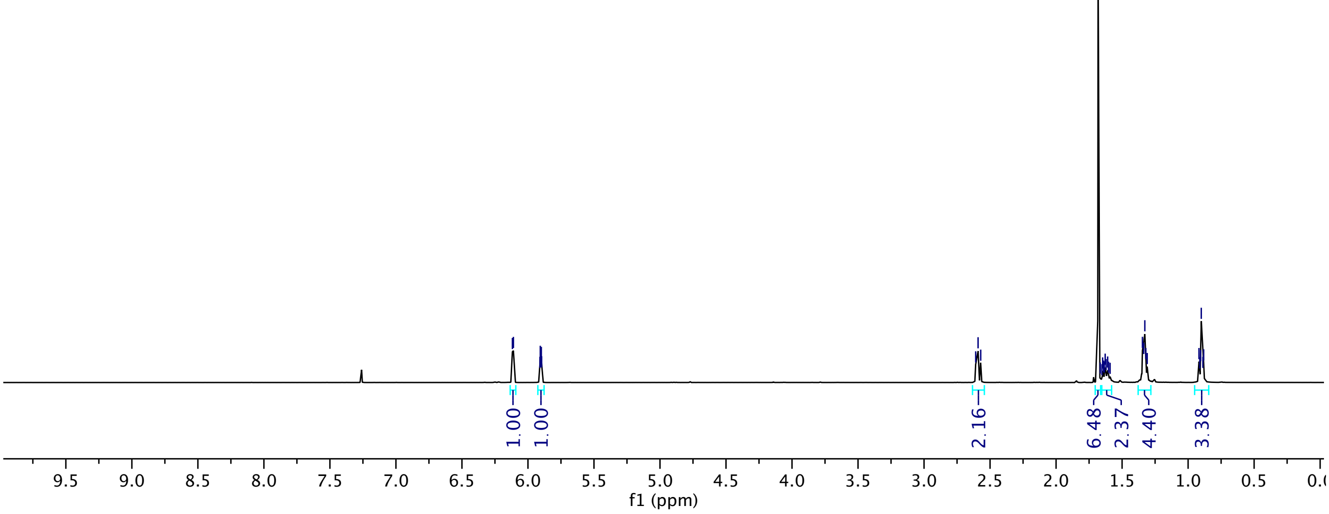

^{13}C NMR (100 MHz, $CDCl_3$)

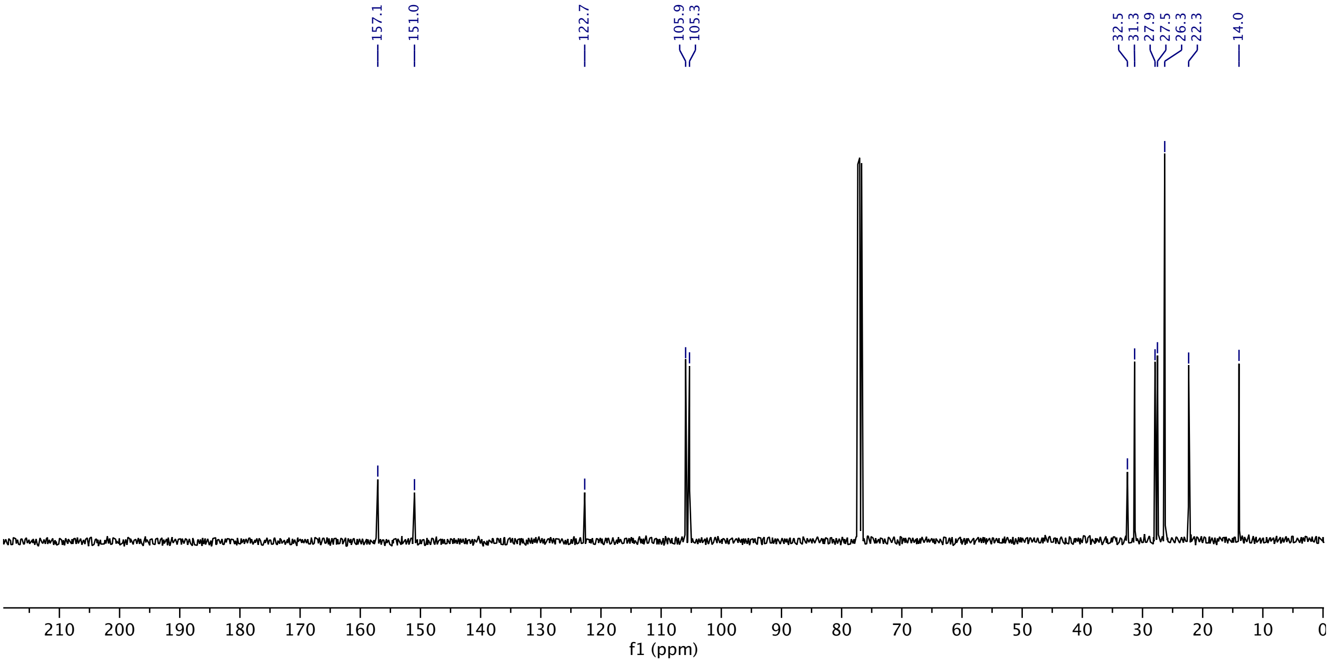

^{1}H NMR (500 MHz, $CDCl_3$)

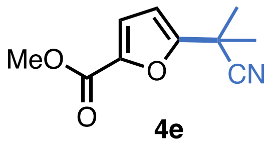

4e

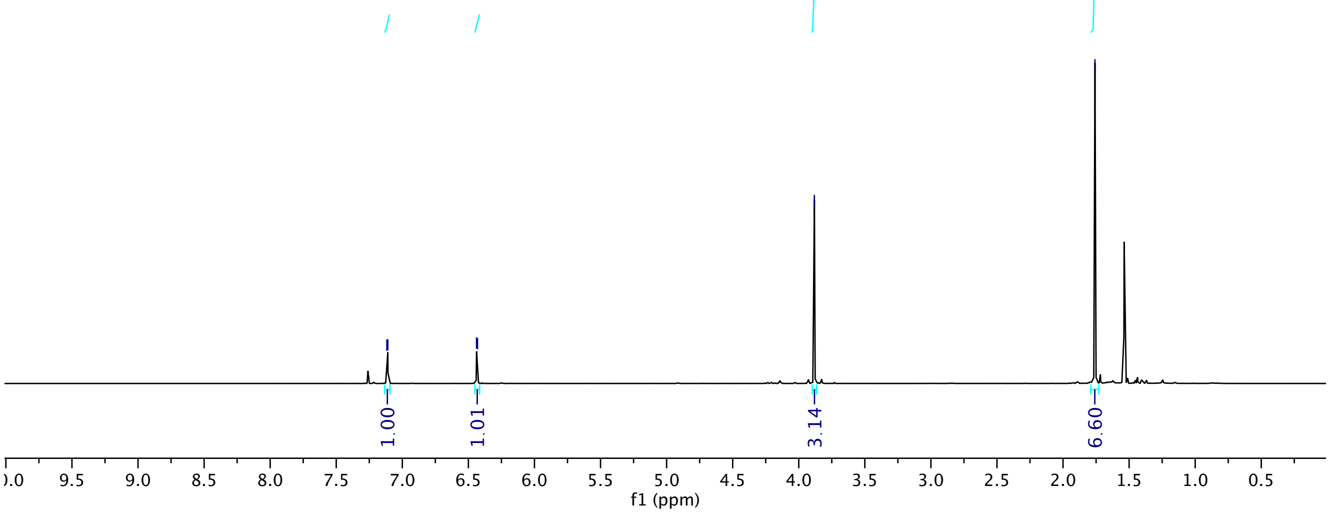

^{13}C NMR (125 MHz, $CDCl_3$)

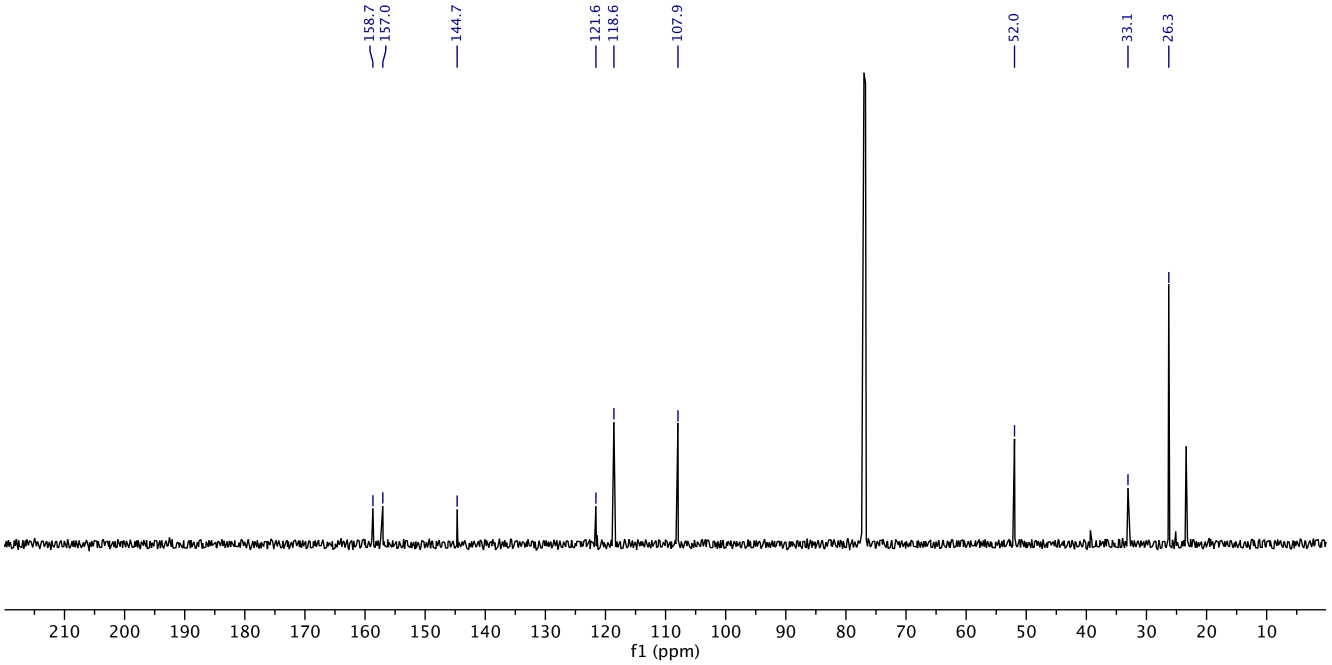

^{1}H NMR (500 MHz, $CDCl_3$)

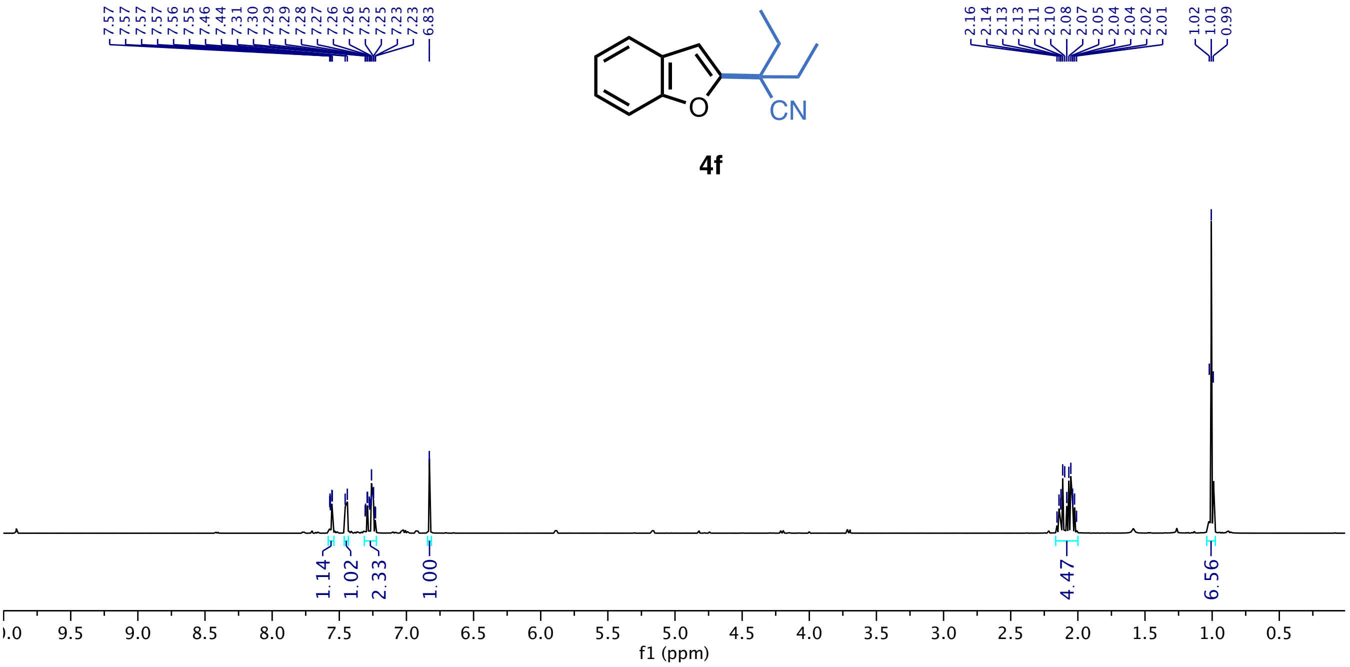

^{13}C NMR (125 MHz, $CDCl_3$)

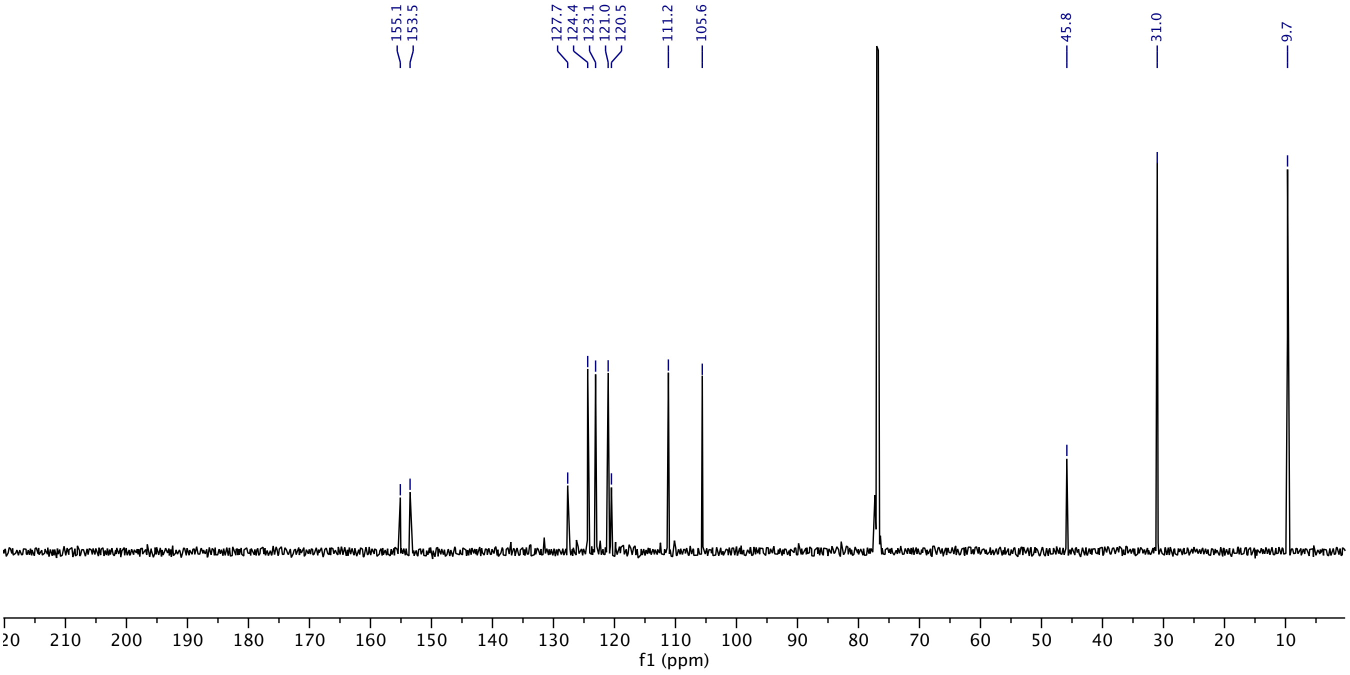

^{1}H NMR (500 MHz, $CDCl_3$)

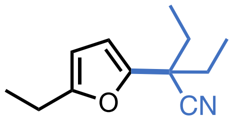

4g

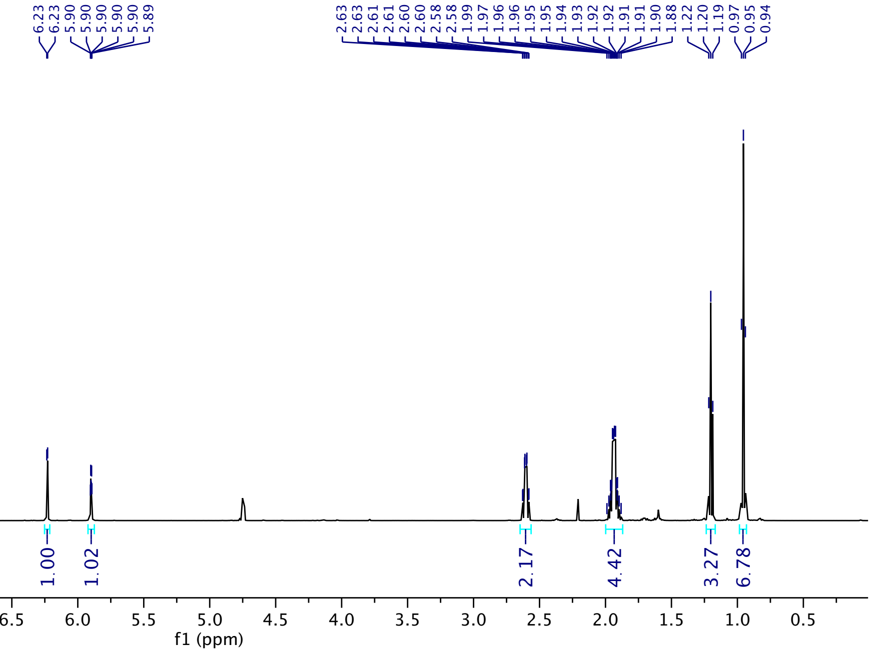

^{13}C NMR (125 MHz, $CDCl_3$)

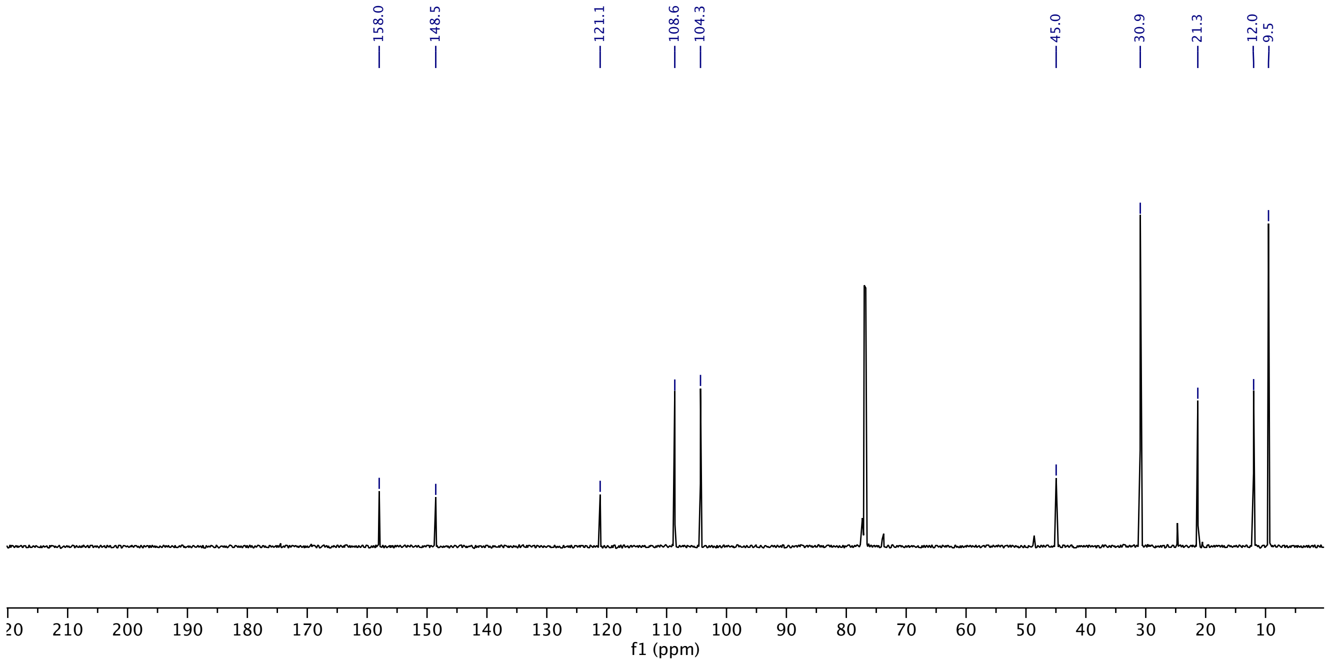

^{1}H NMR (500 MHz, $CDCl_3$)

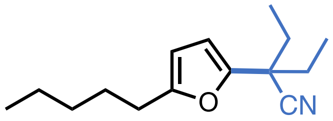

4h

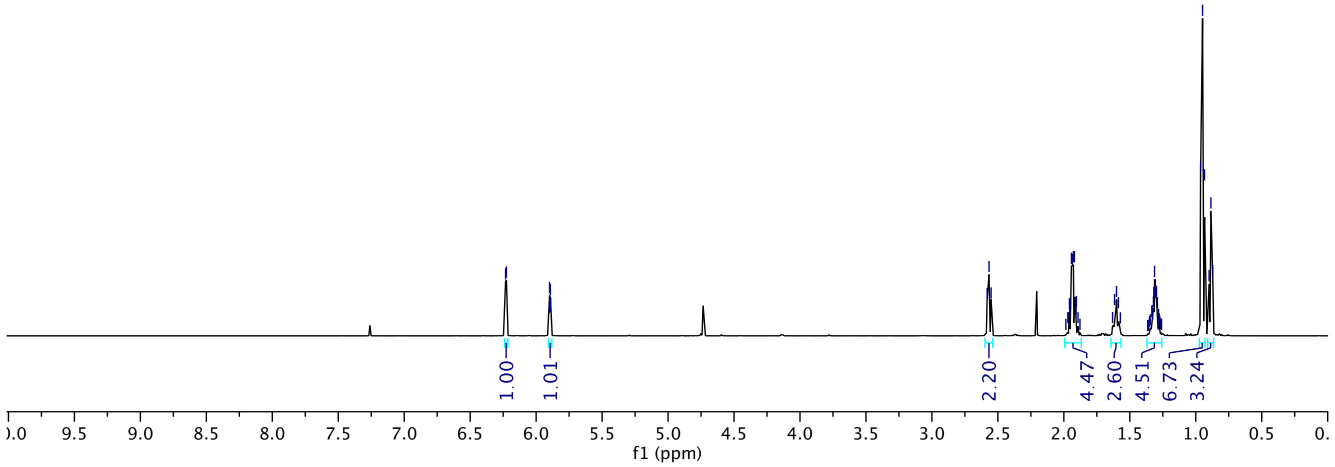

^{13}C NMR (125 MHz, $CDCl_3$)

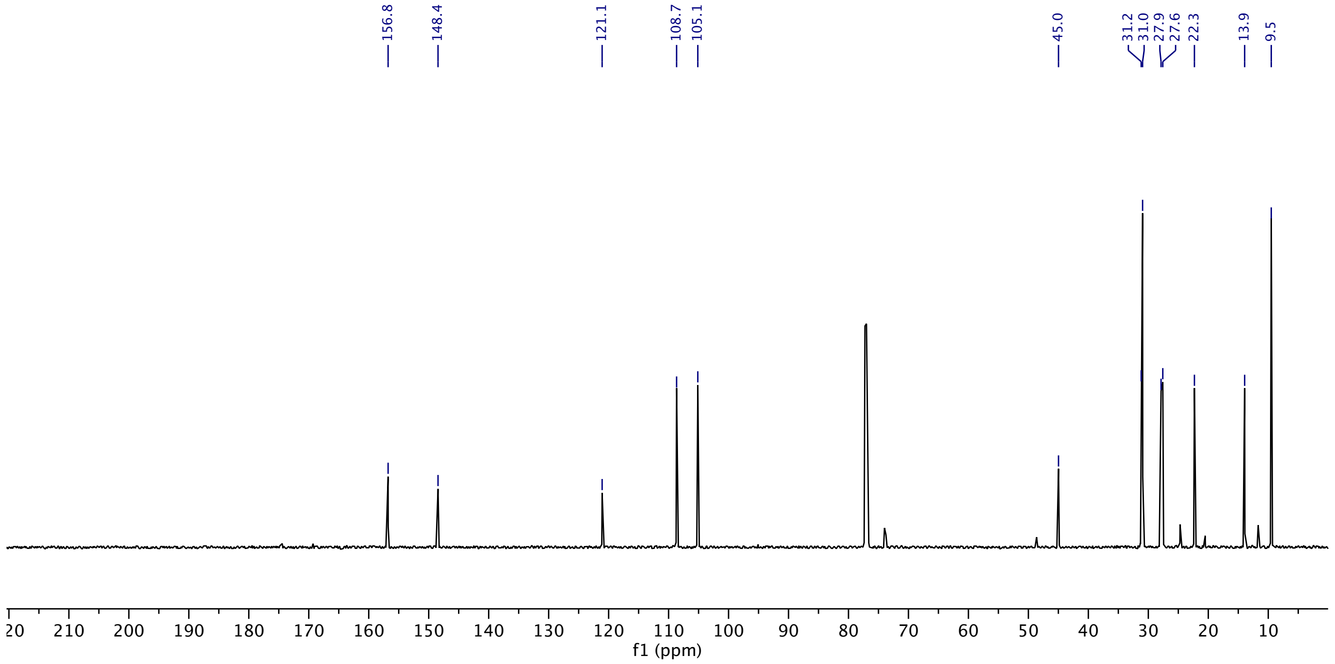

^{1}H NMR (700 MHz, CDCl$_3$)

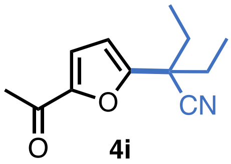

4i

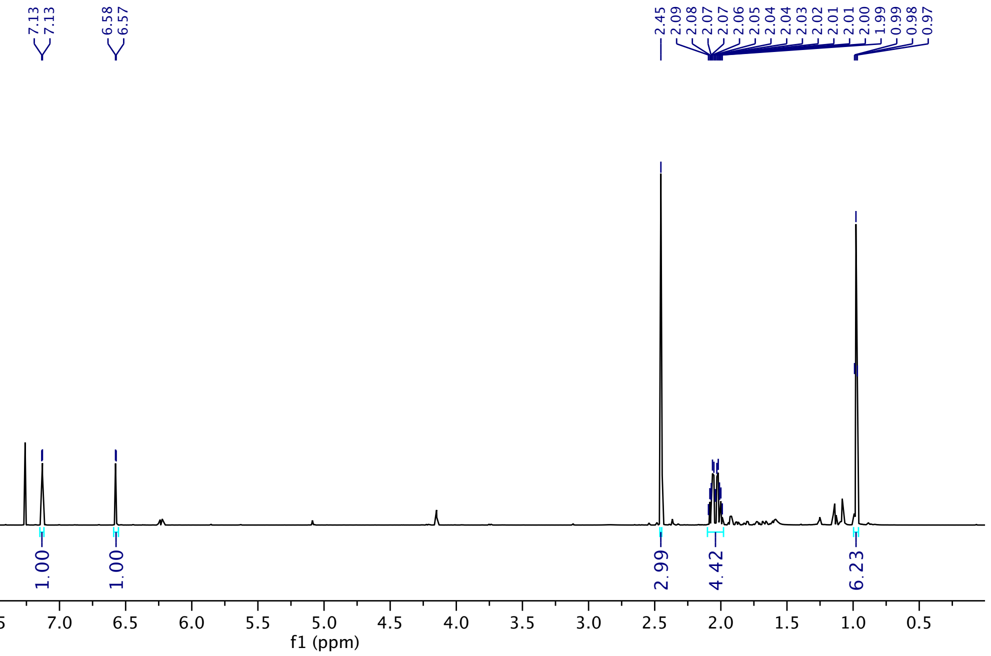

^{13}C NMR (175 MHz, CDCl$_3$)

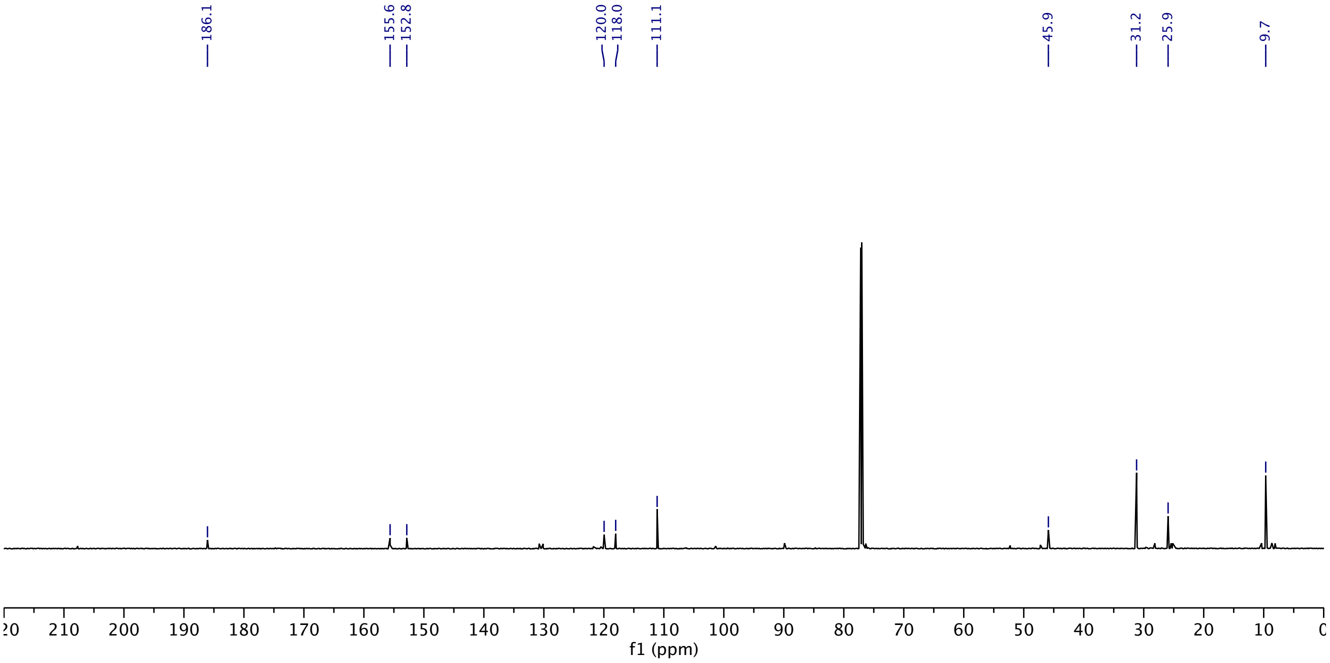

^{1}H NMR (400 MHz, $CDCl_3$)

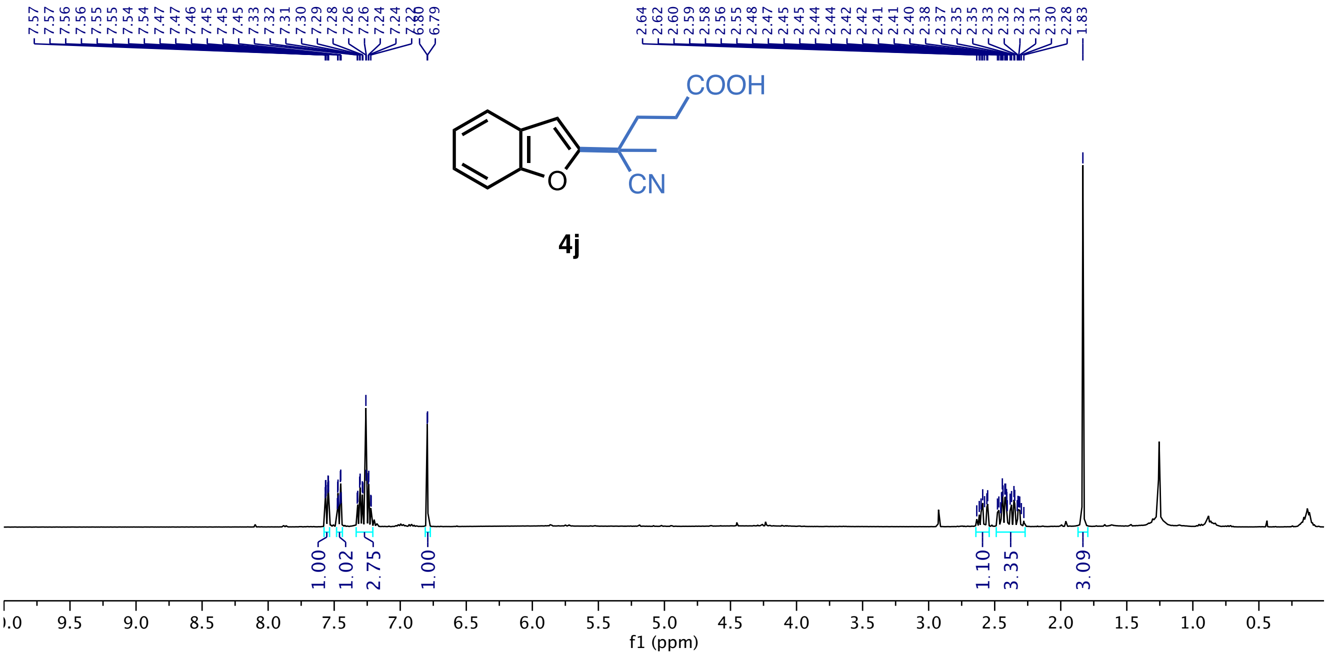

^{13}C NMR (100 MHz, $CDCl_3$)

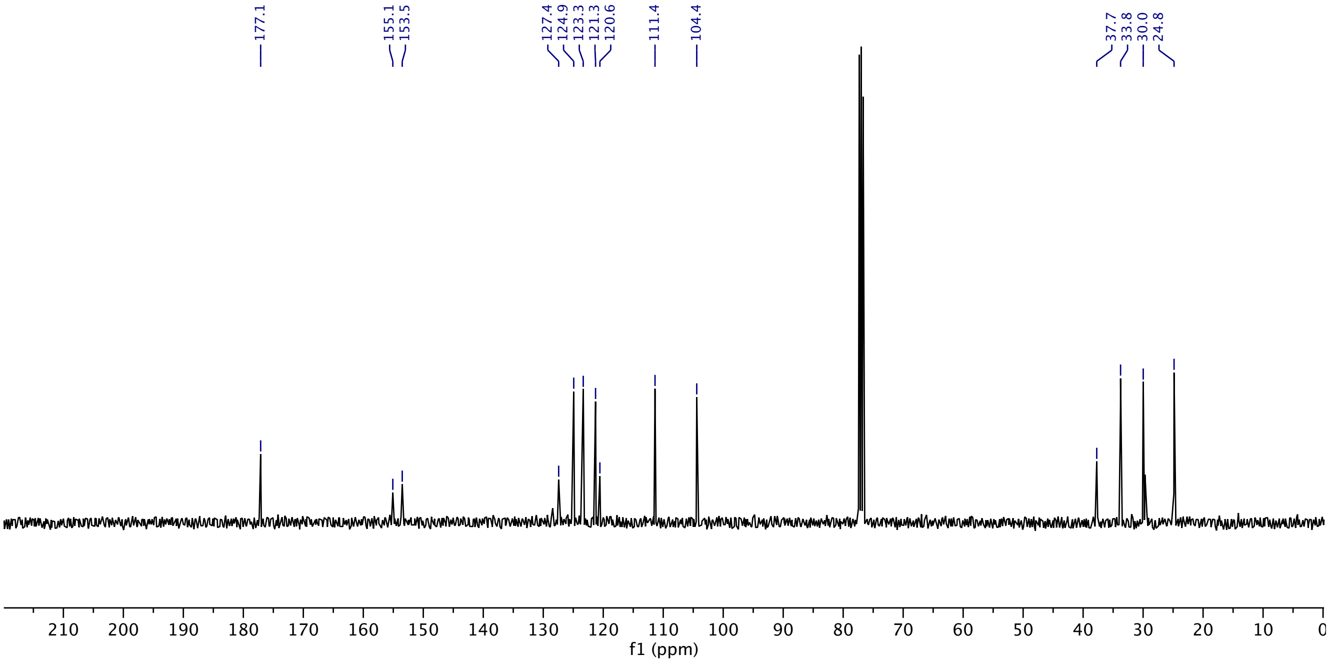

^{1}H NMR (400 MHz, $CDCl_3$)

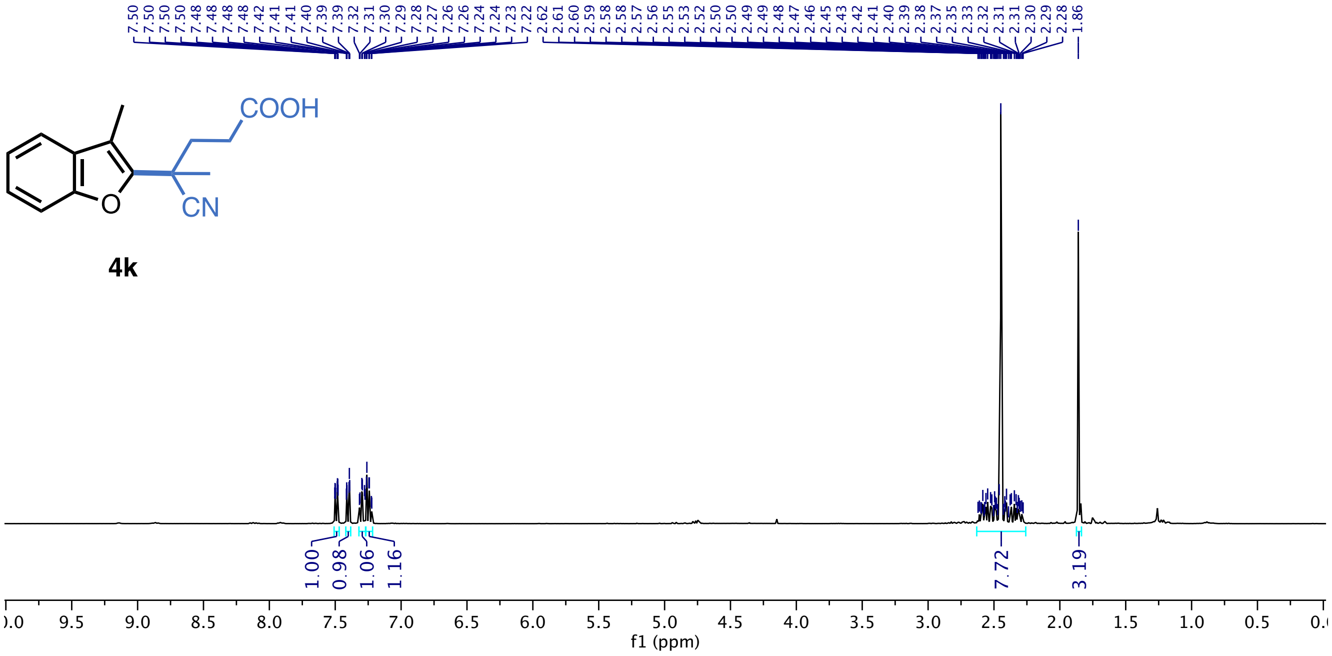

^{13}C NMR (100 MHz, $CDCl_3$)

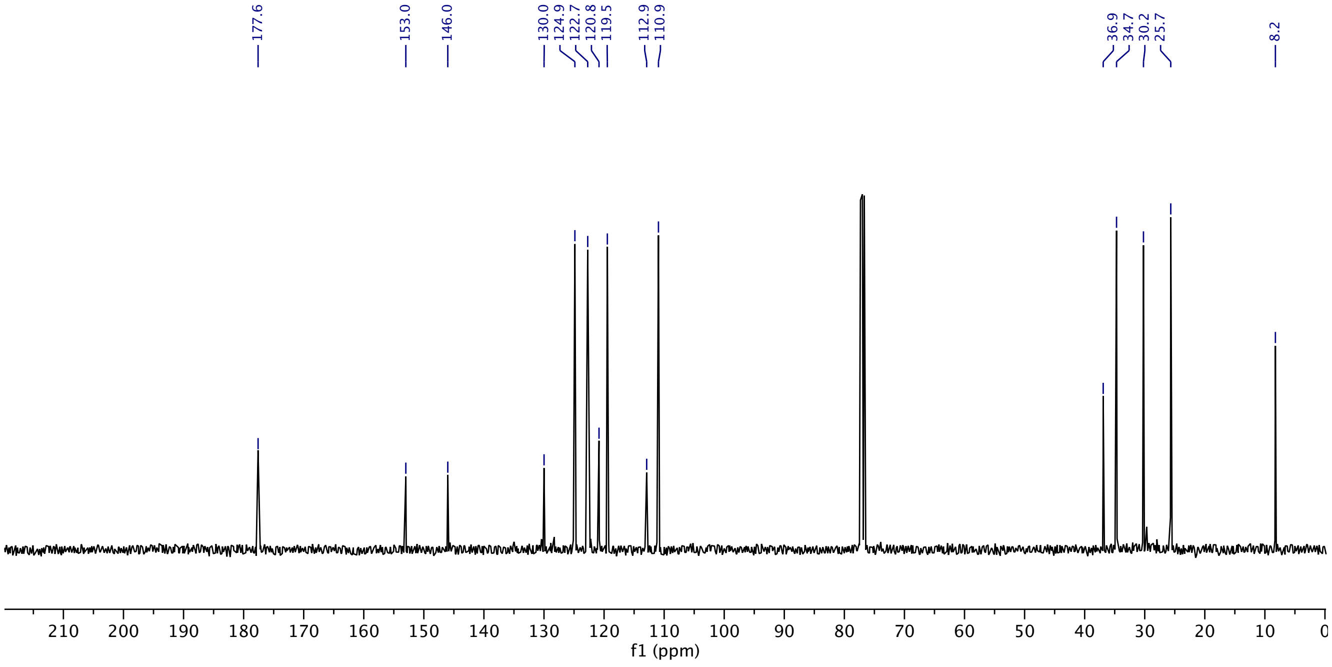

^{1}H NMR (400 MHz, $CDCl_3$)

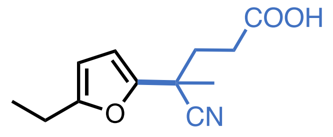

4l

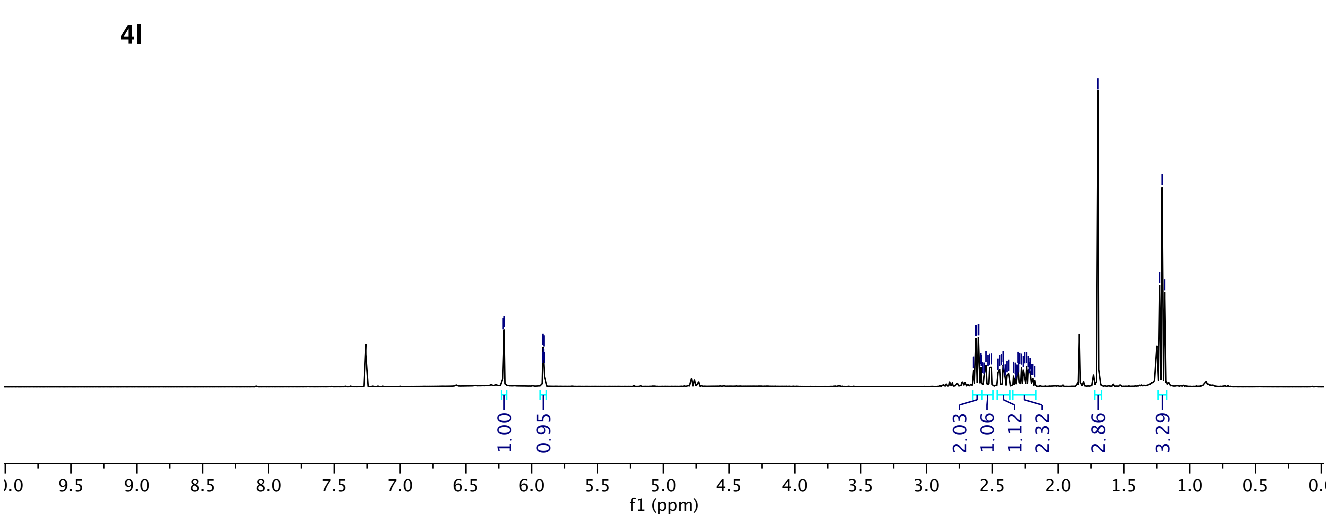

^{13}C NMR (100 MHz, $CDCl_3$)

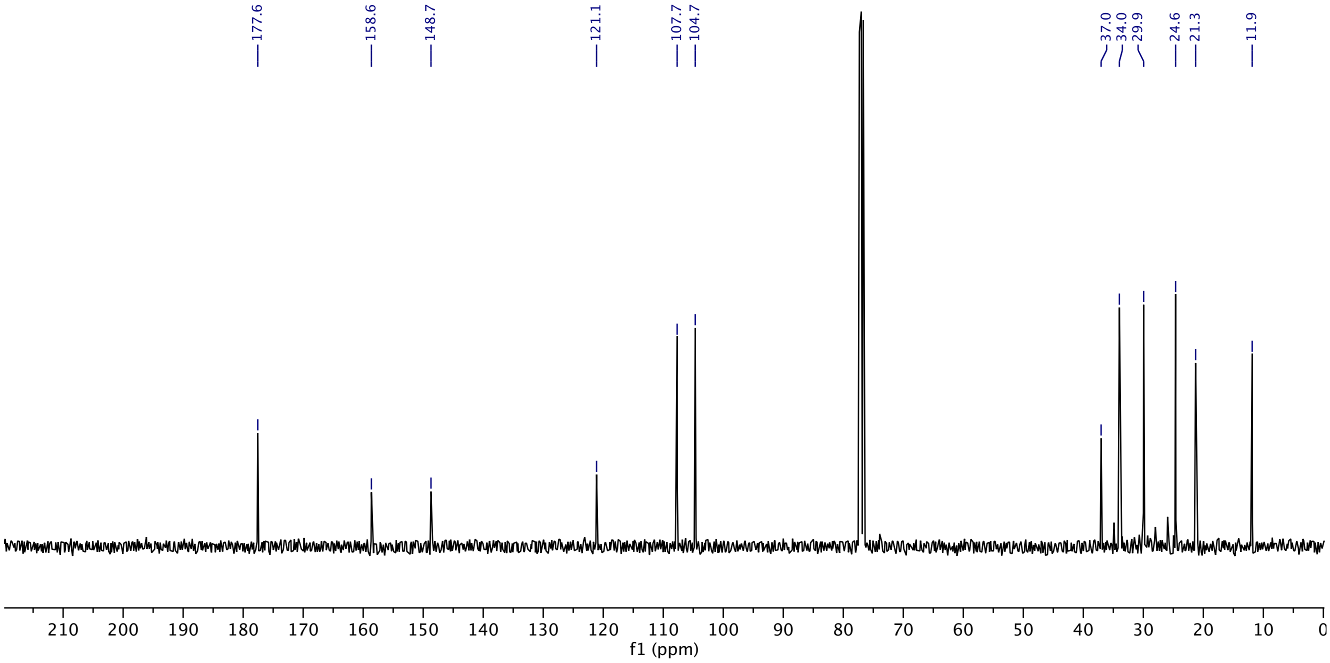

^{1}H NMR (400 MHz, $CDCl_3$)

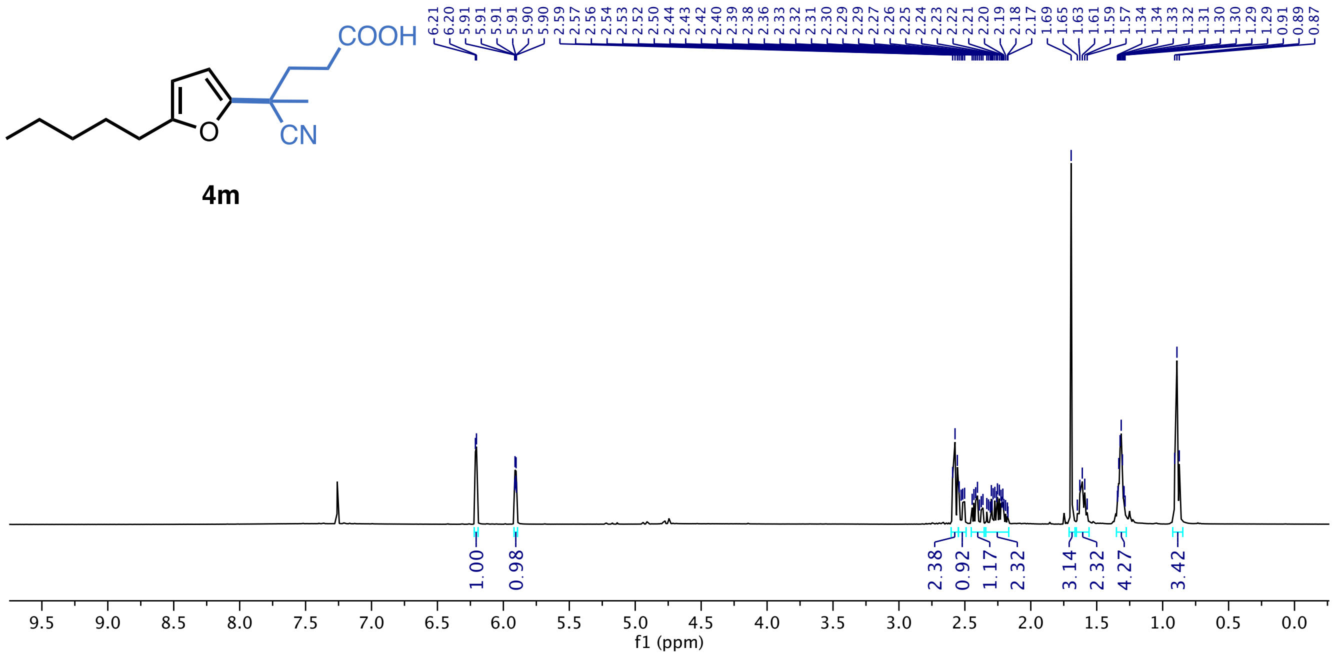

4m

^{13}C NMR (100 MHz, $CDCl_3$)

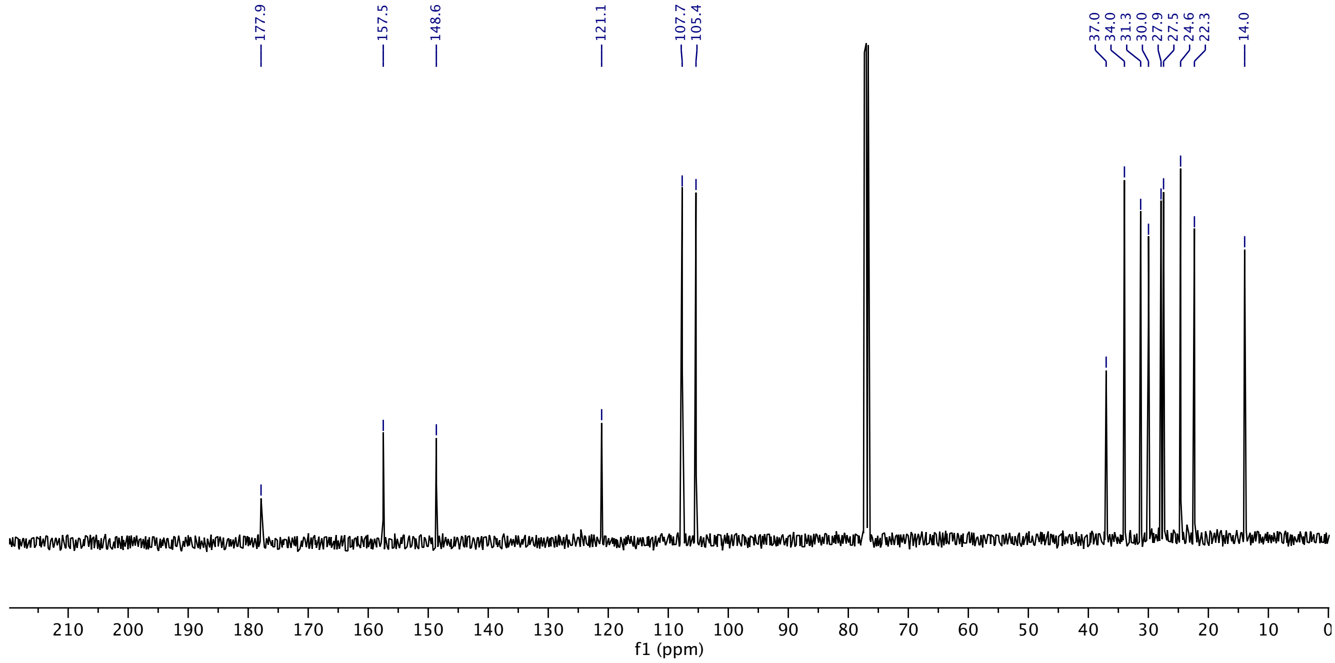

^{1}H NMR (700 MHz, $CDCl_3$)

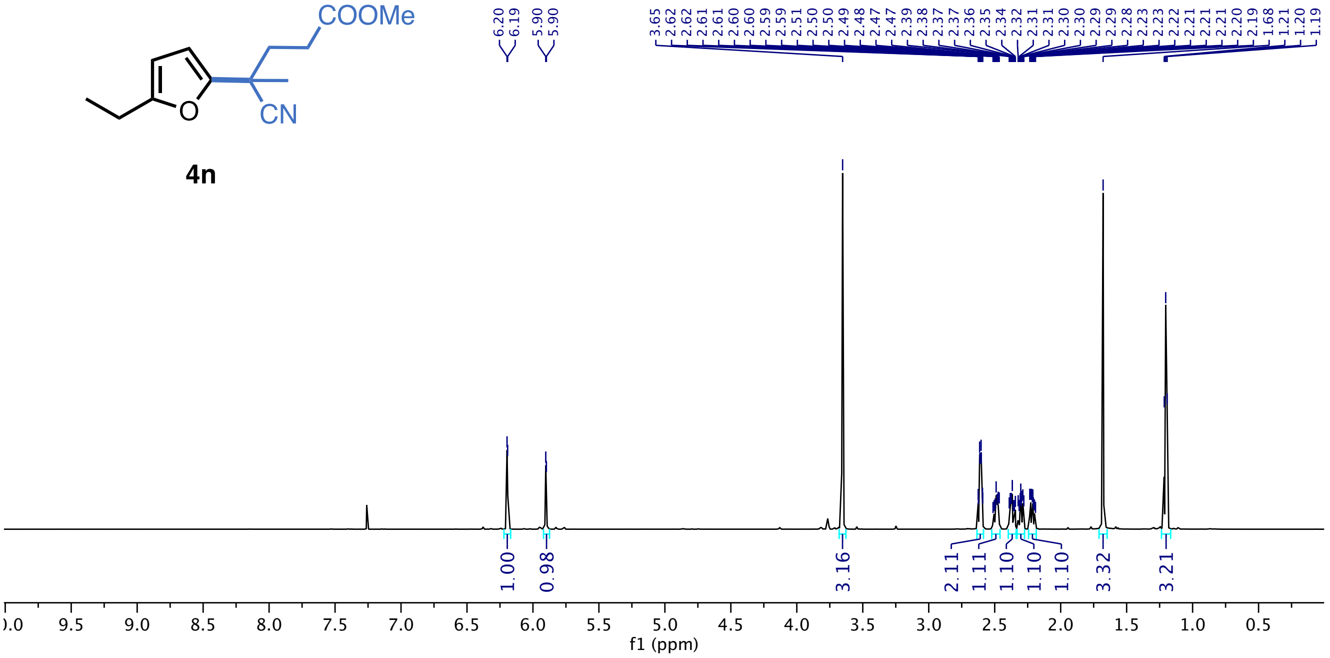

^{13}C NMR (175 MHz, $CDCl_3$)

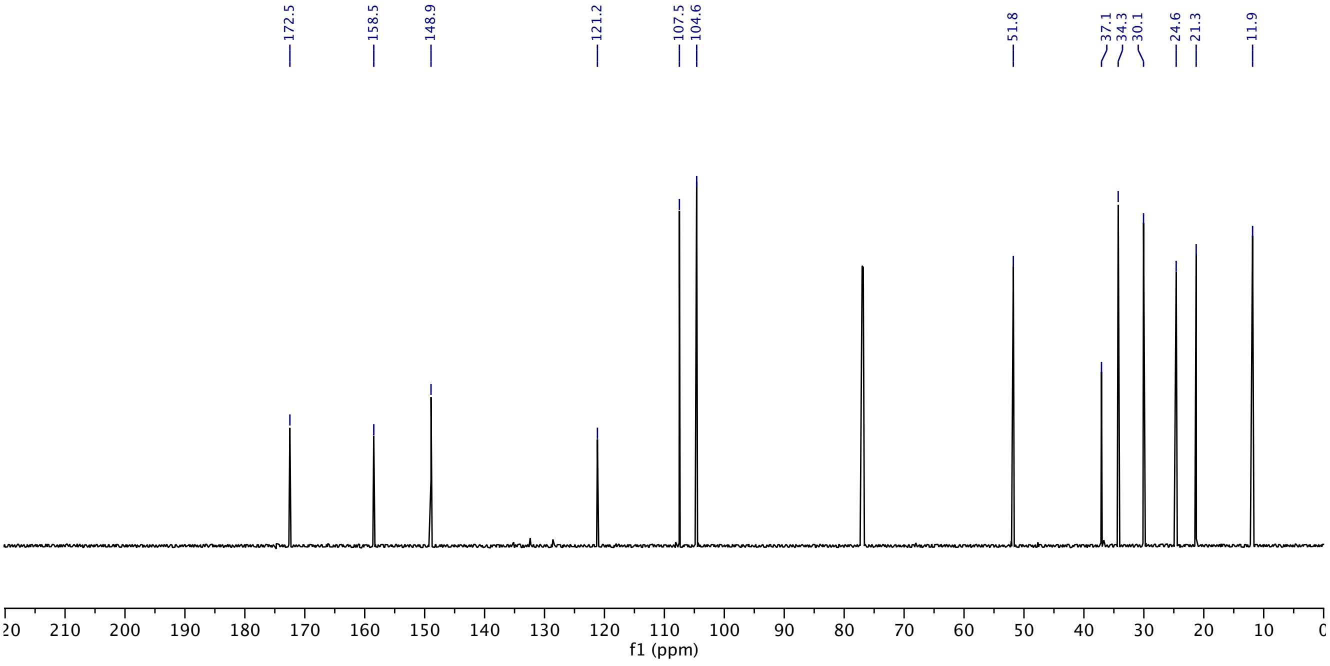

^{1}H NMR (700 MHz, $CDCl_3$)

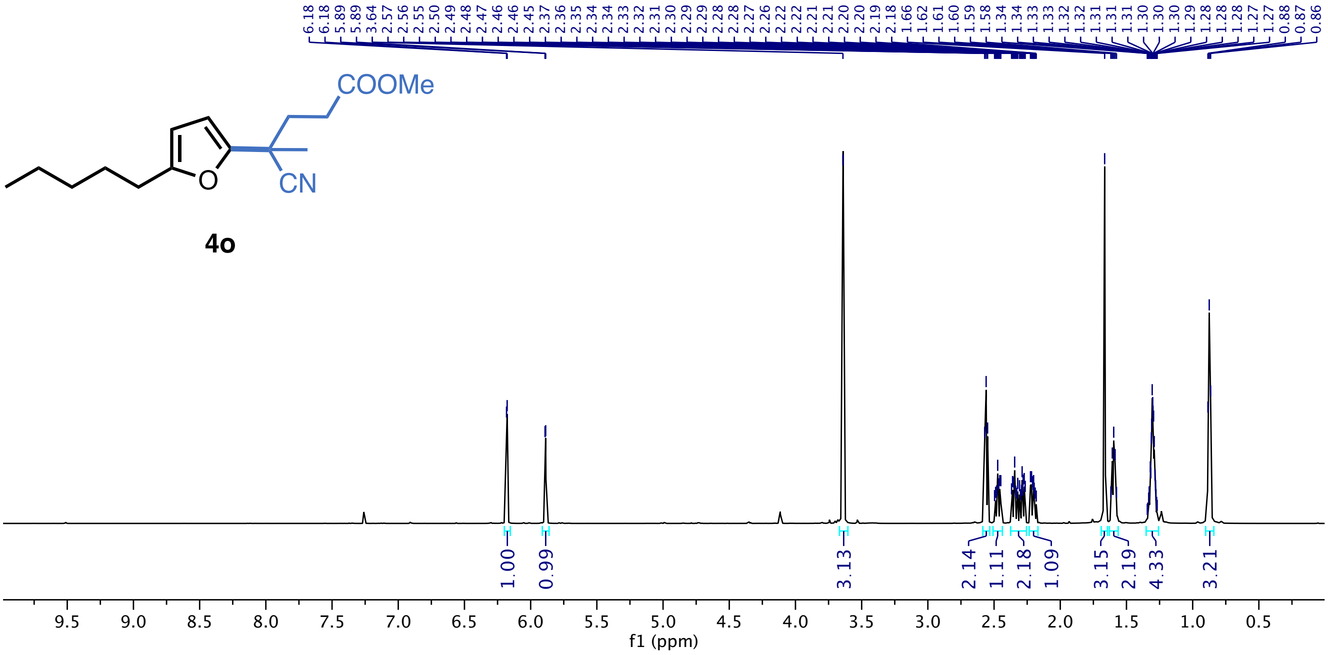

^{13}C NMR (175 MHz, $CDCl_3$)

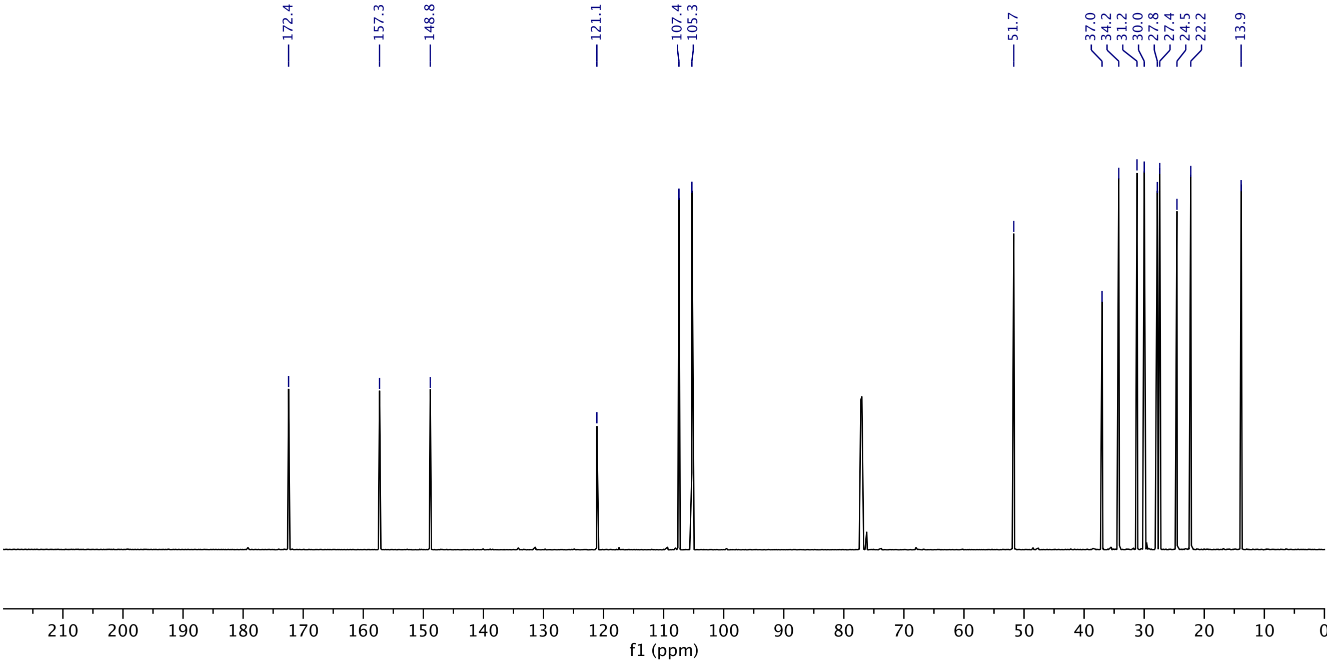

^1H NMR (400 MHz, $CDCl_3$)

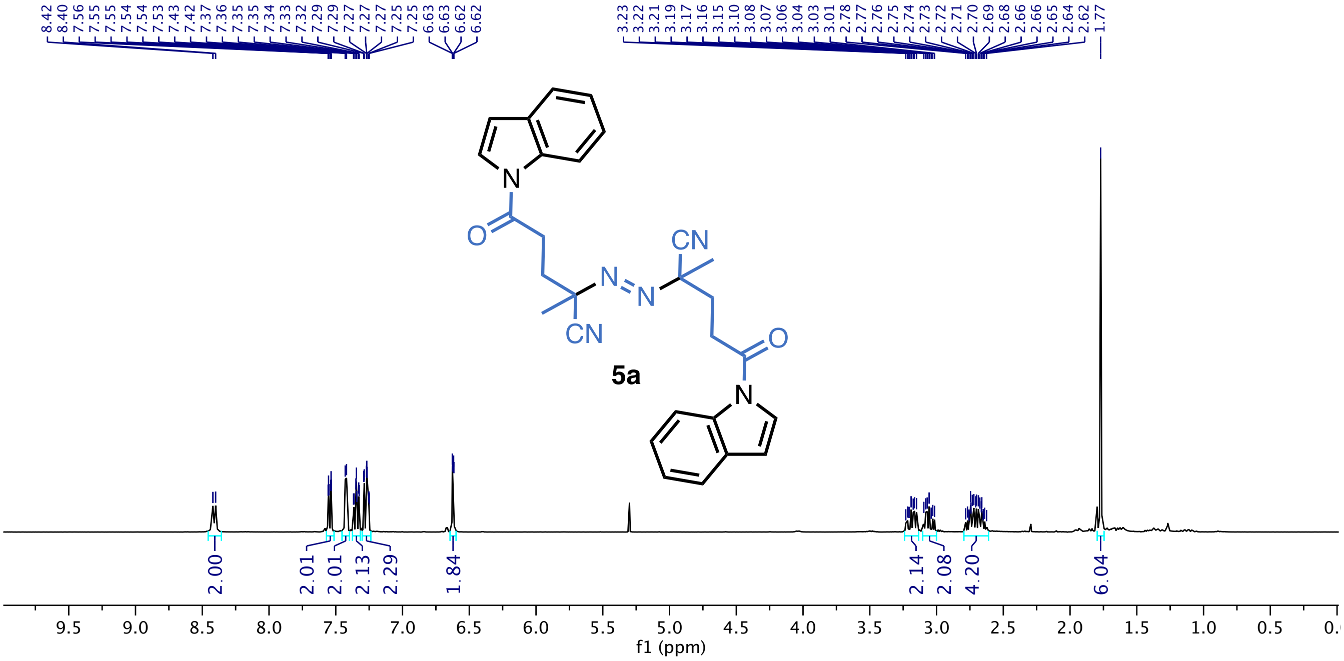

^{13}C NMR (100 MHz, $CDCl_3$)

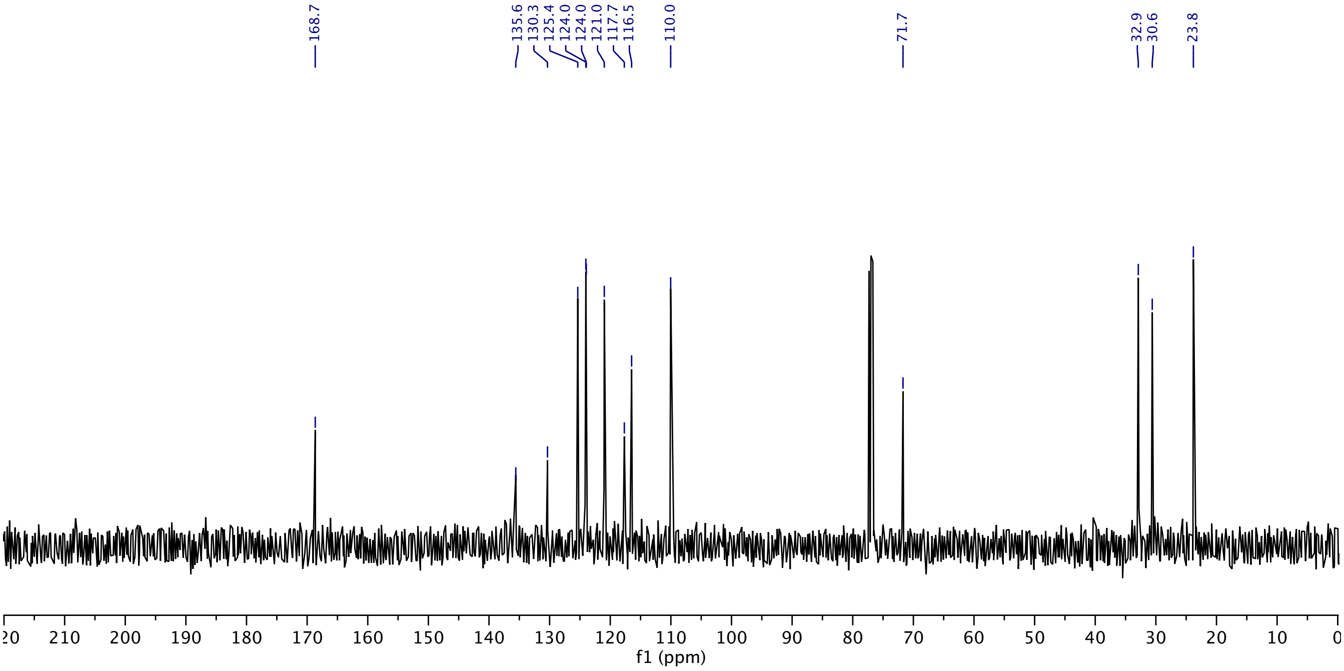

^{1}H NMR (500 MHz, $CDCl_3$)

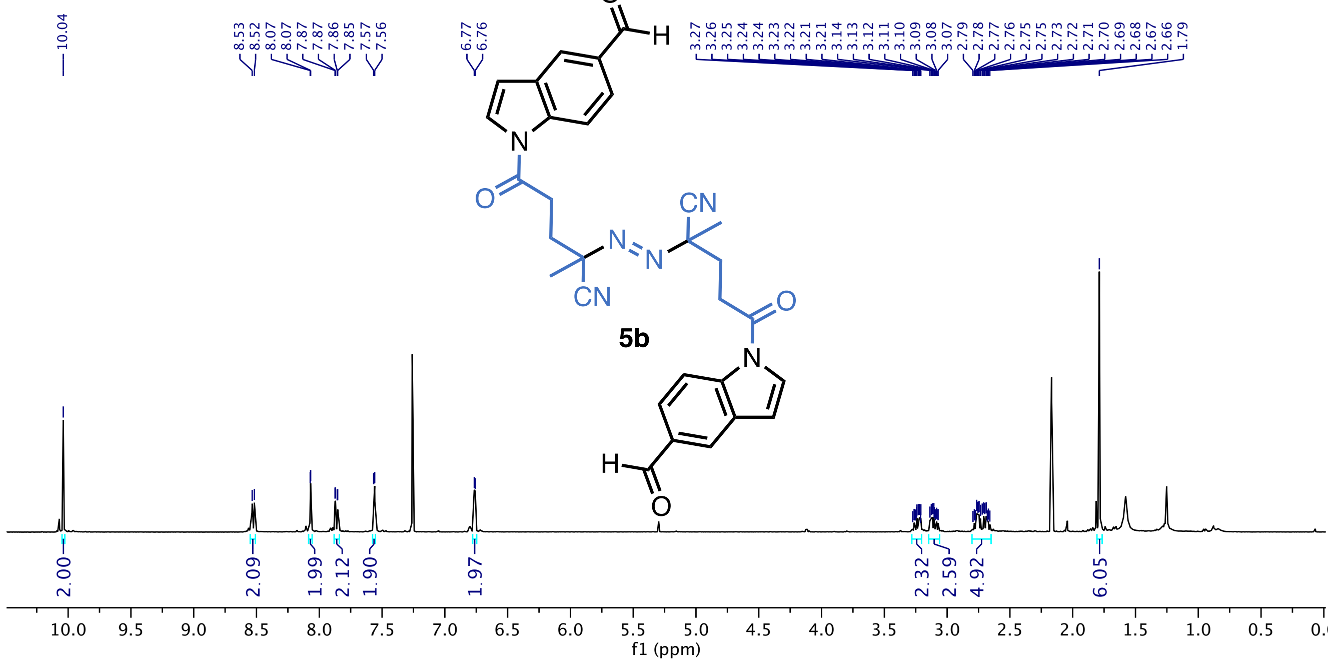

^{13}C NMR (125 MHz, $CDCl_3$)

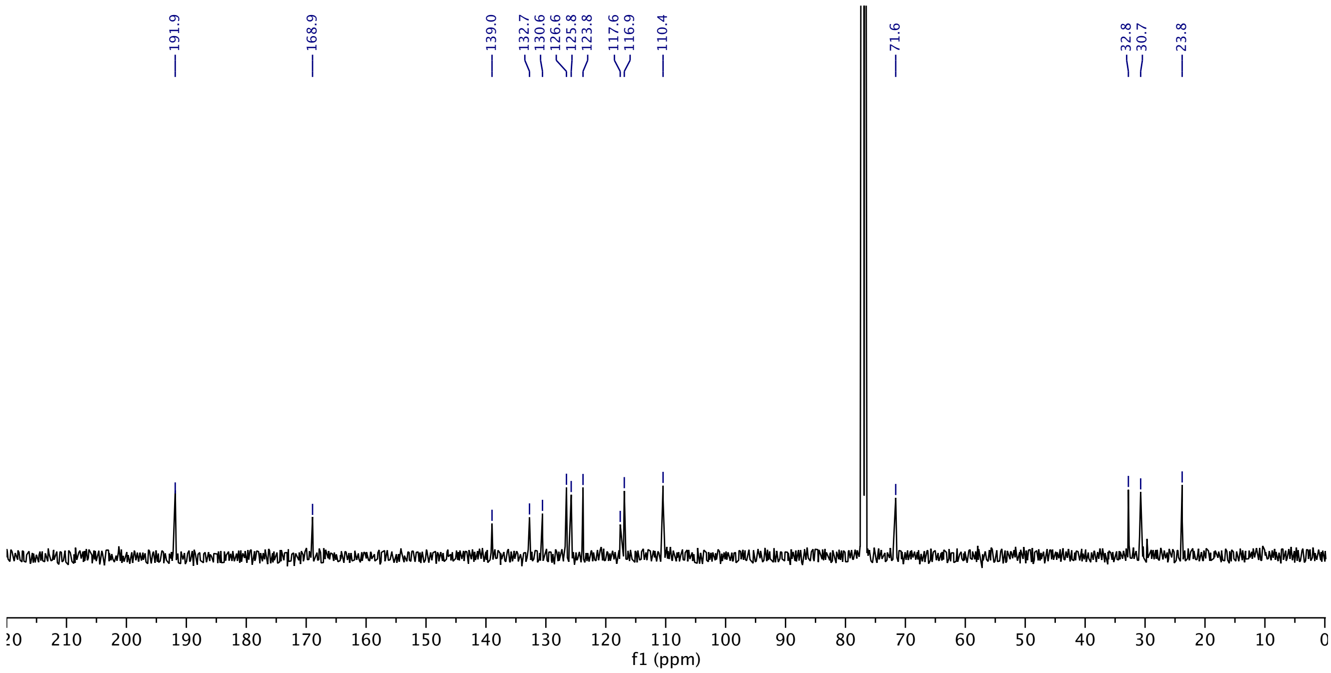

^{1}H NMR (700 MHz, $CDCl_3$)

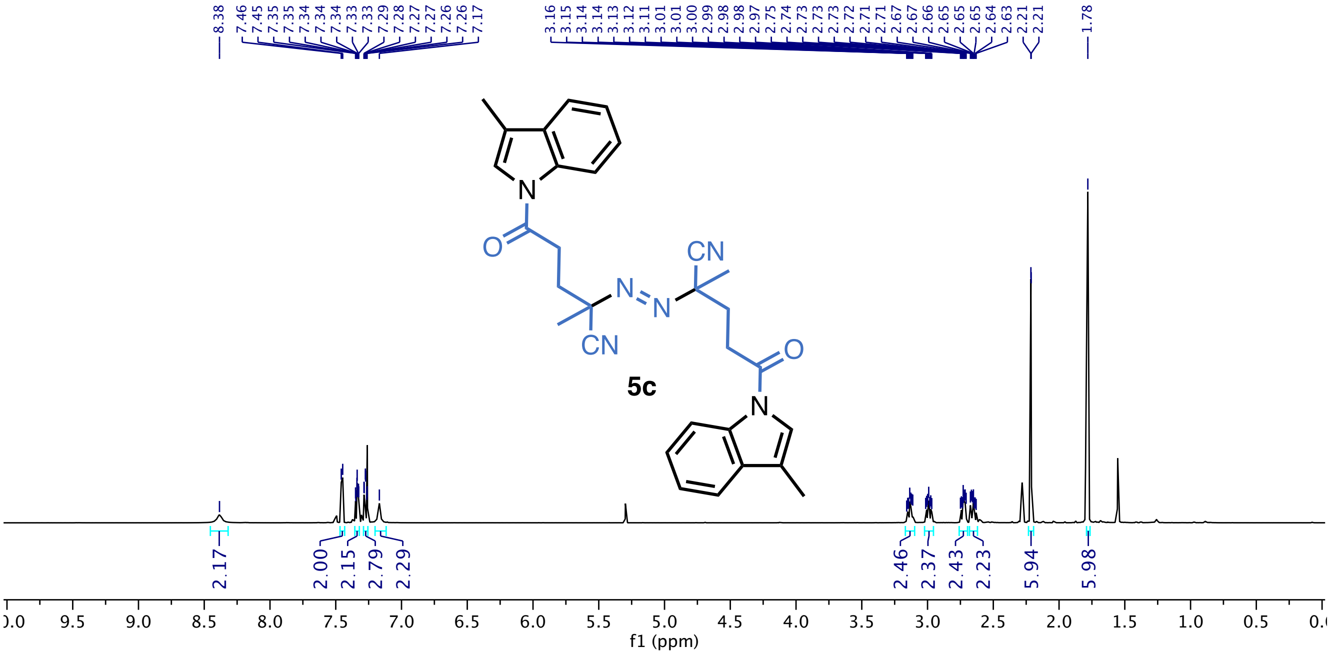

^{13}C NMR (175 MHz, $CDCl_3$)

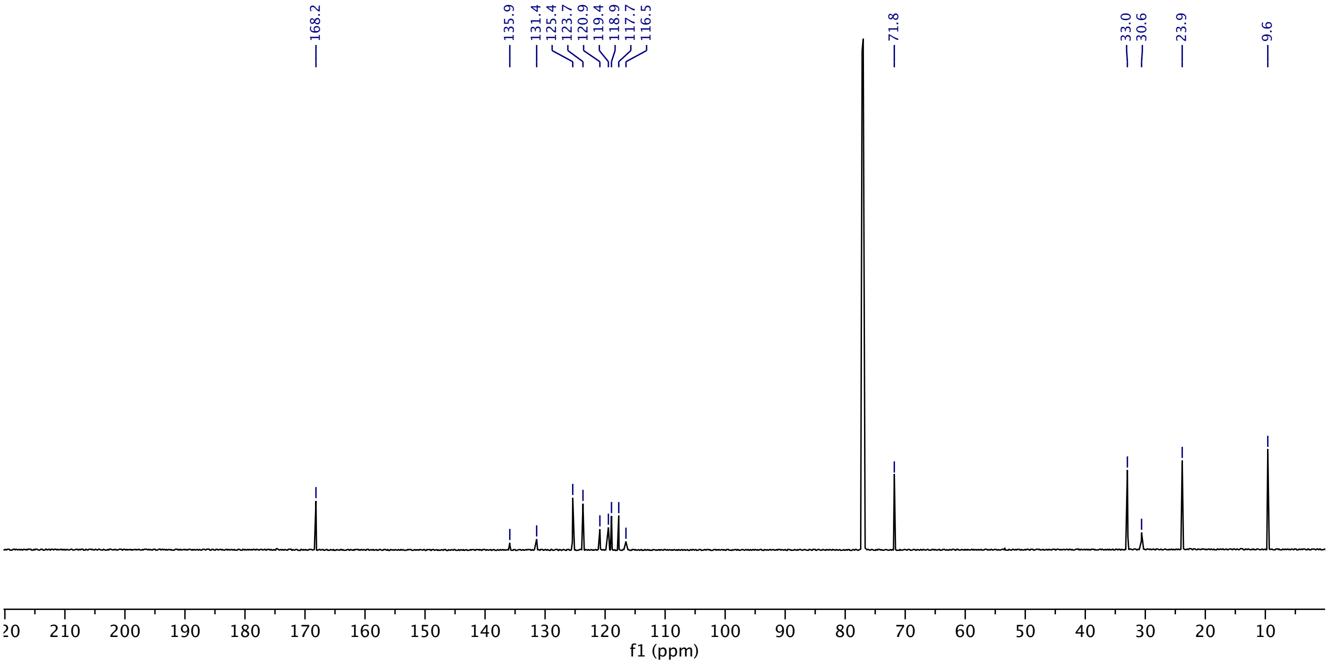

^{1}H NMR (400 MHz, $CDCl_3$)

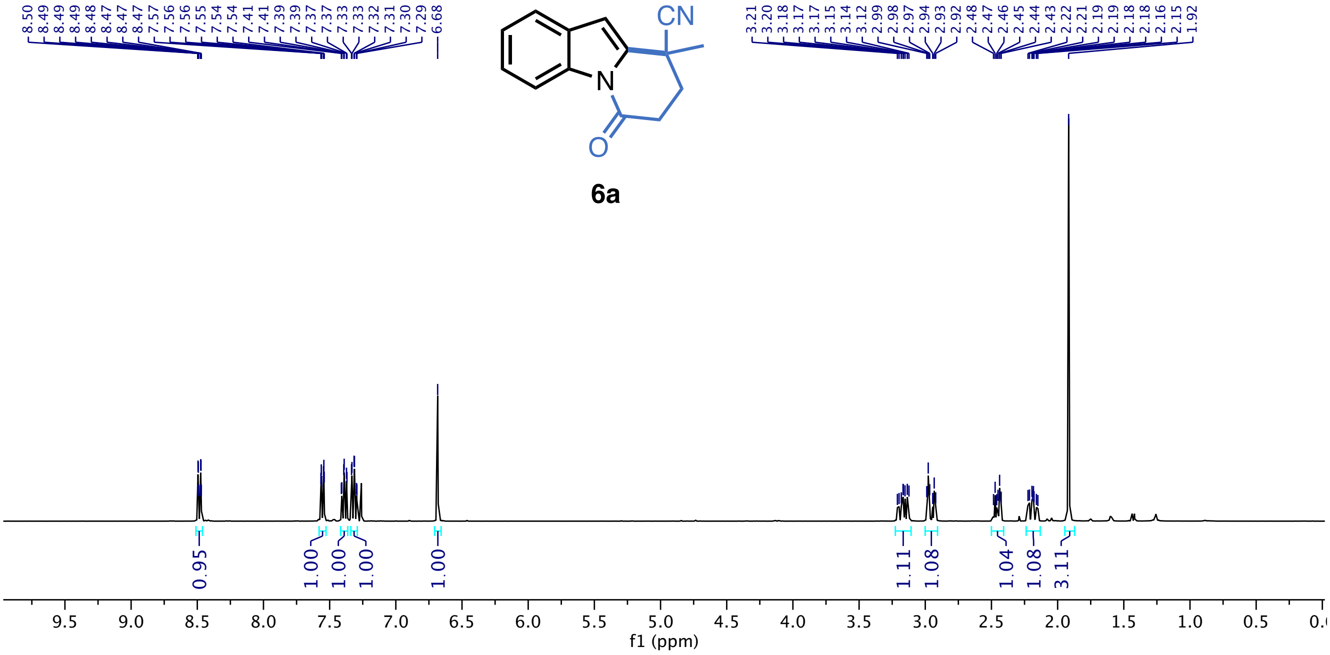

^{13}C NMR (100 MHz, $CDCl_3$)

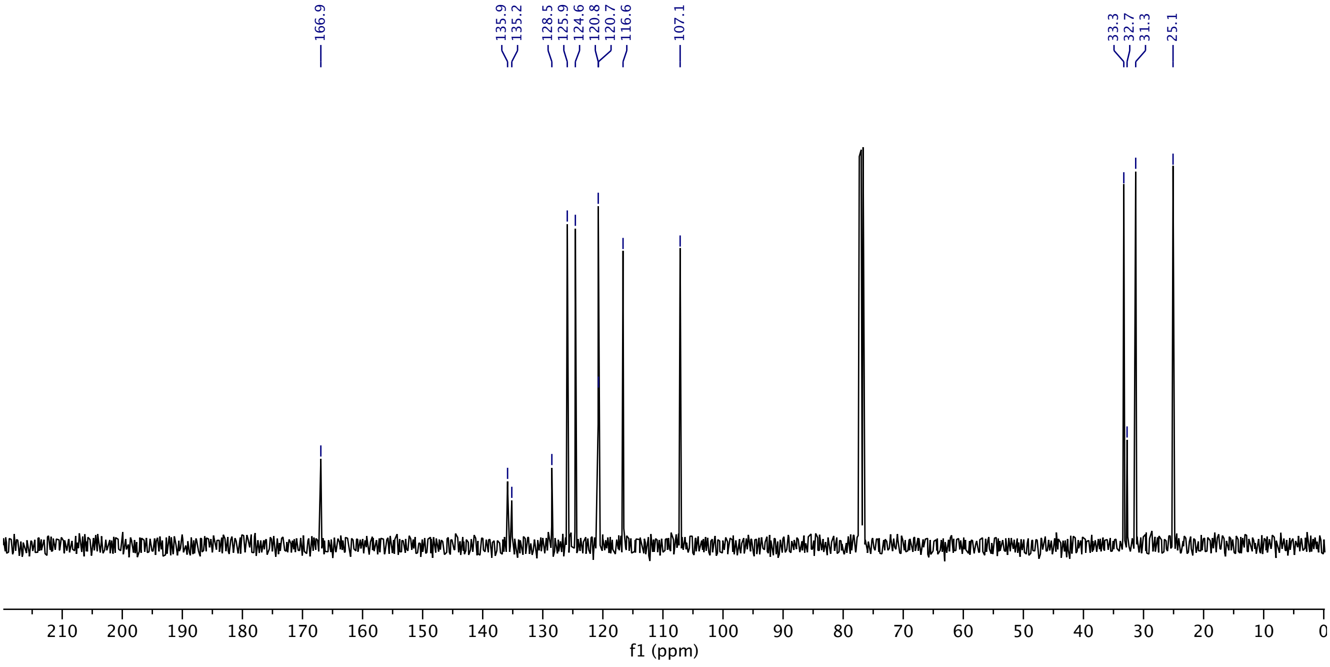

^{1}H NMR (500 MHz, $CDCl_3$)

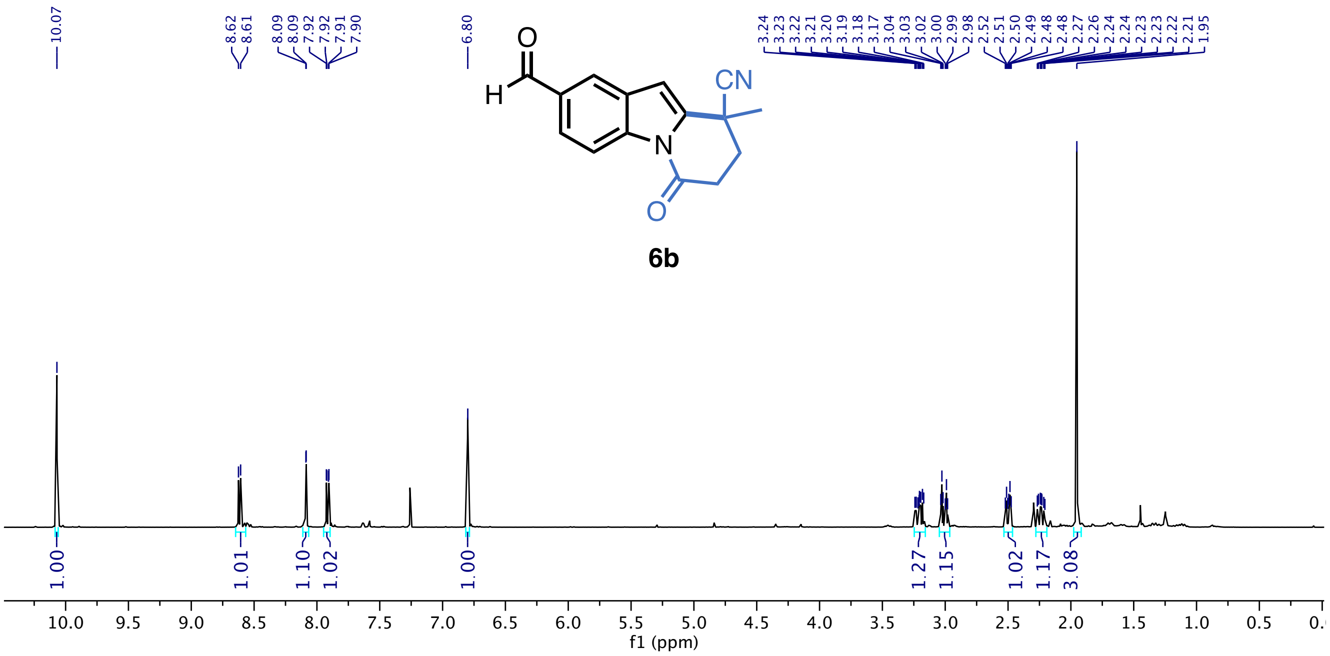

^{13}C NMR (125 MHz, $CDCl_3$)

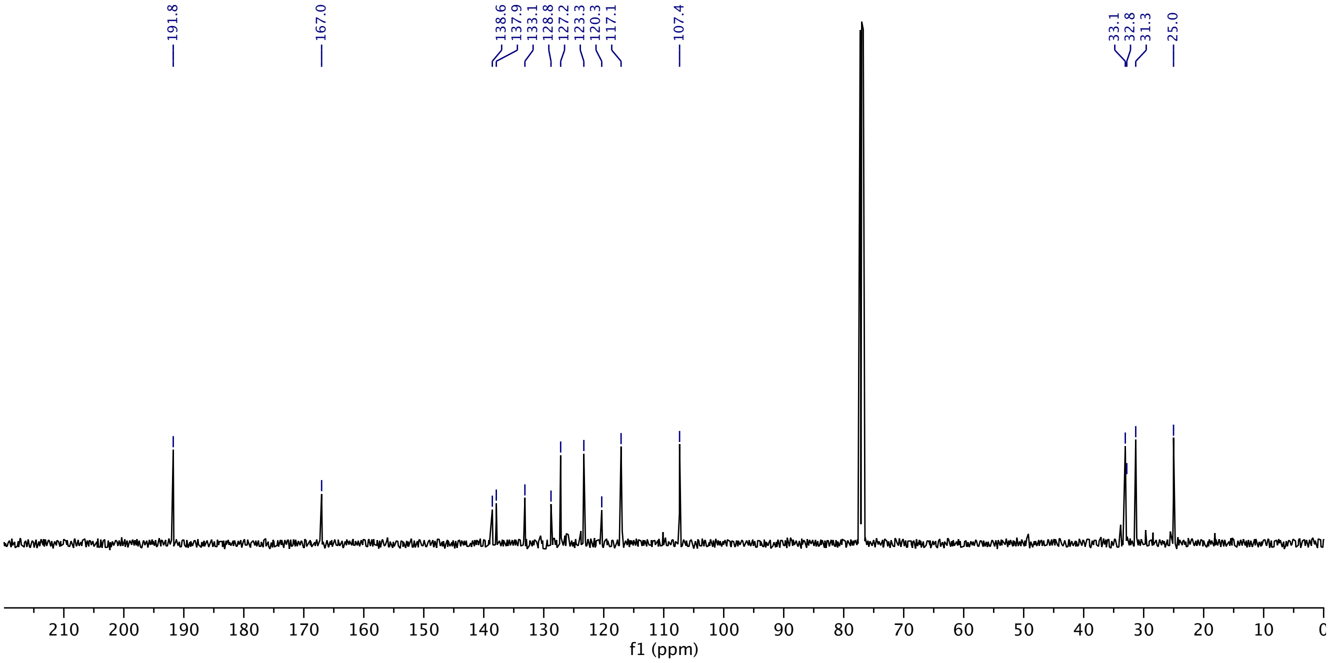

^{1}H NMR (700 MHz, $CDCl_3$)

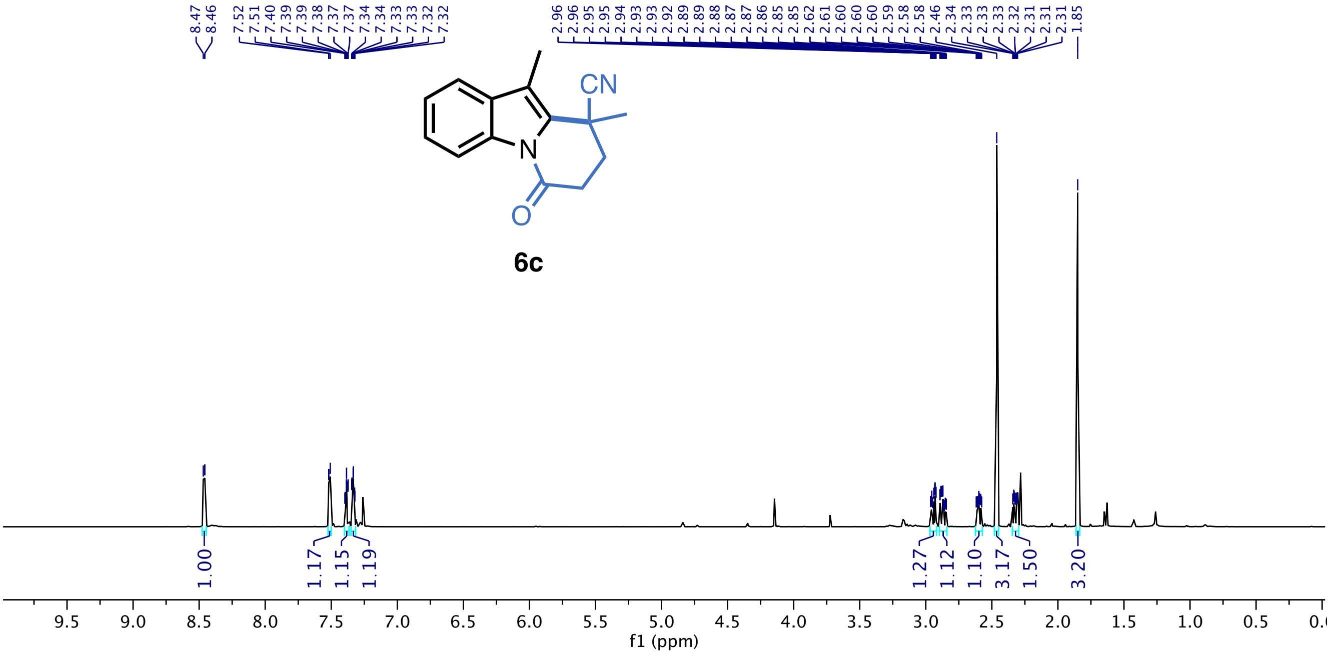

^{13}C NMR (175 MHz, $CDCl_3$)

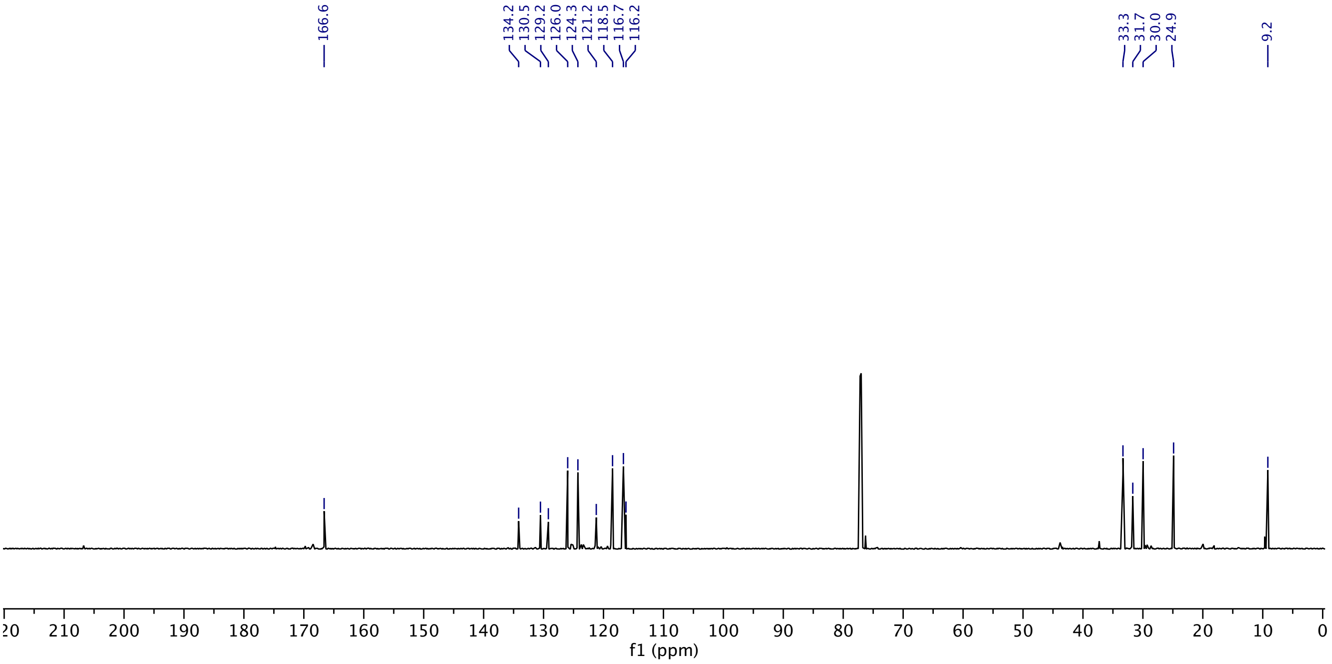

^{1}H NMR (400 MHz, $CDCl_3$)

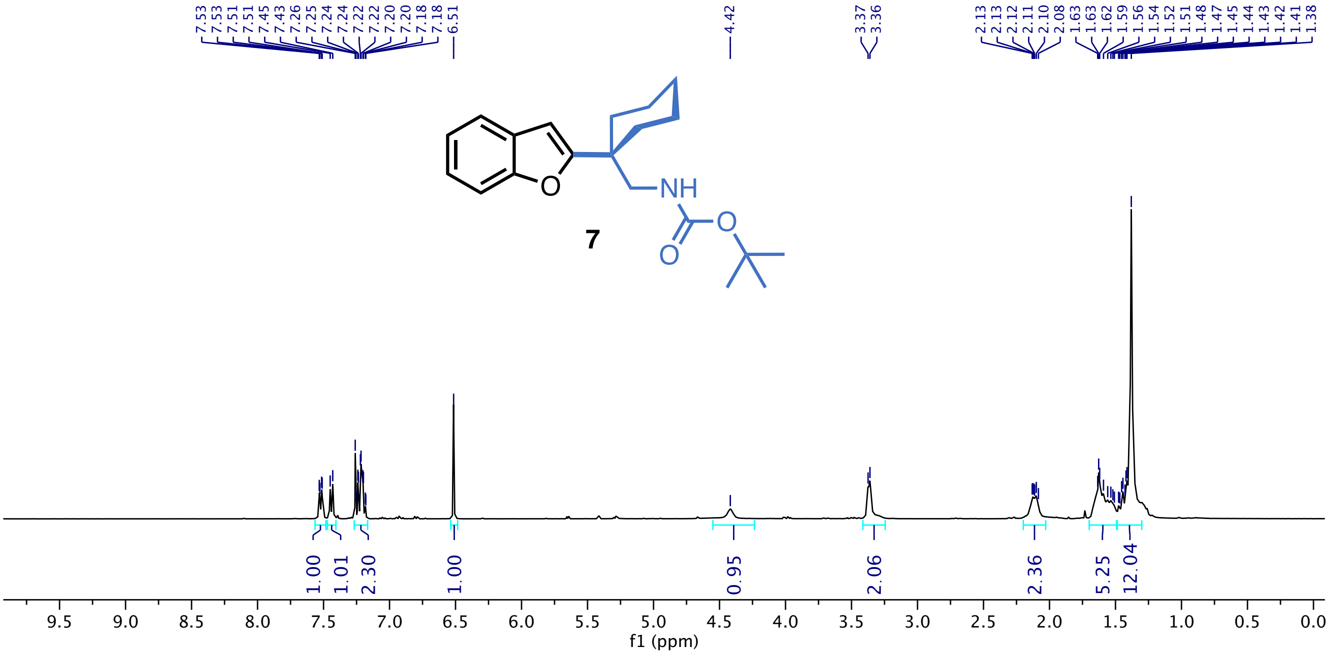

^{13}C NMR (100 MHz, $CDCl_3$)

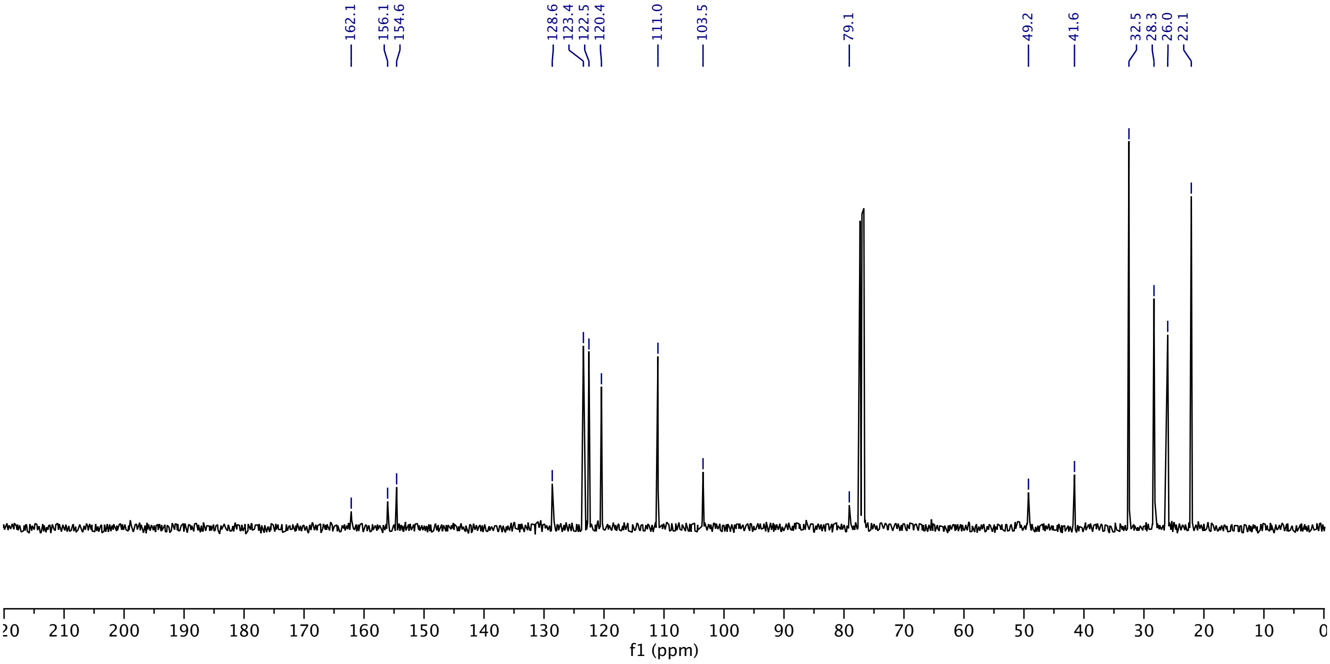

^1H NMR (400 MHz, $CDCl_3$)

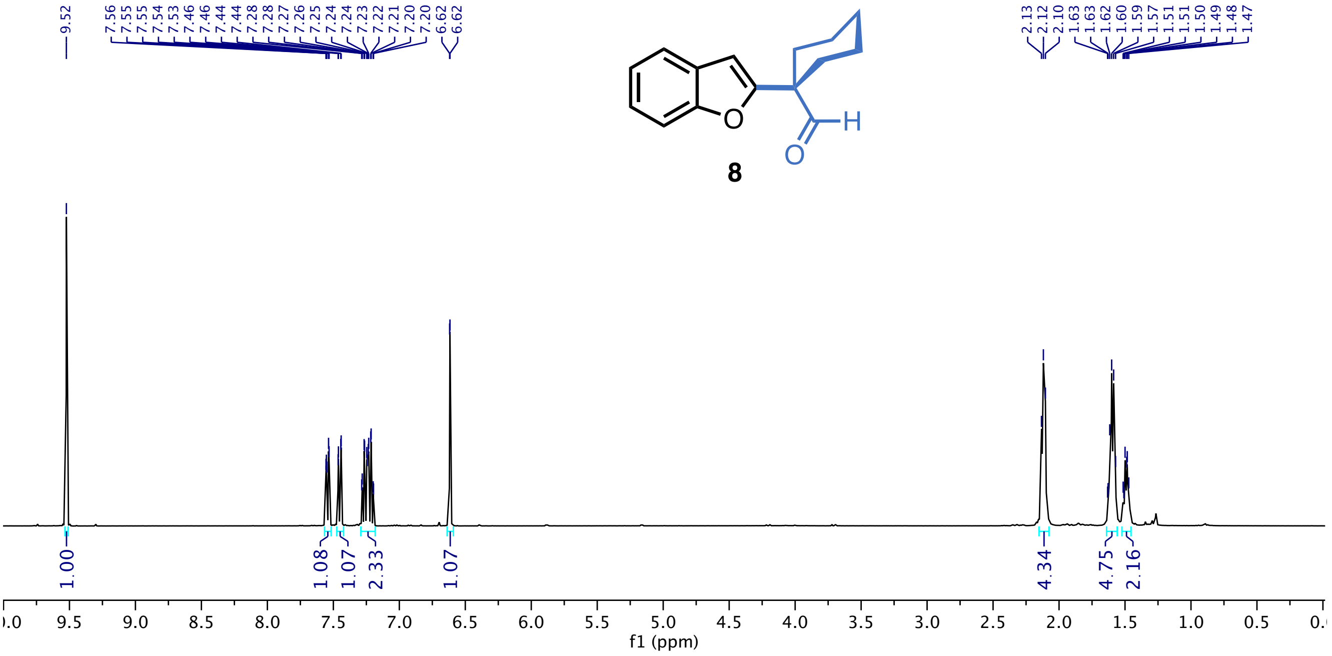

^{13}C NMR (100 MHz, $CDCl_3$)

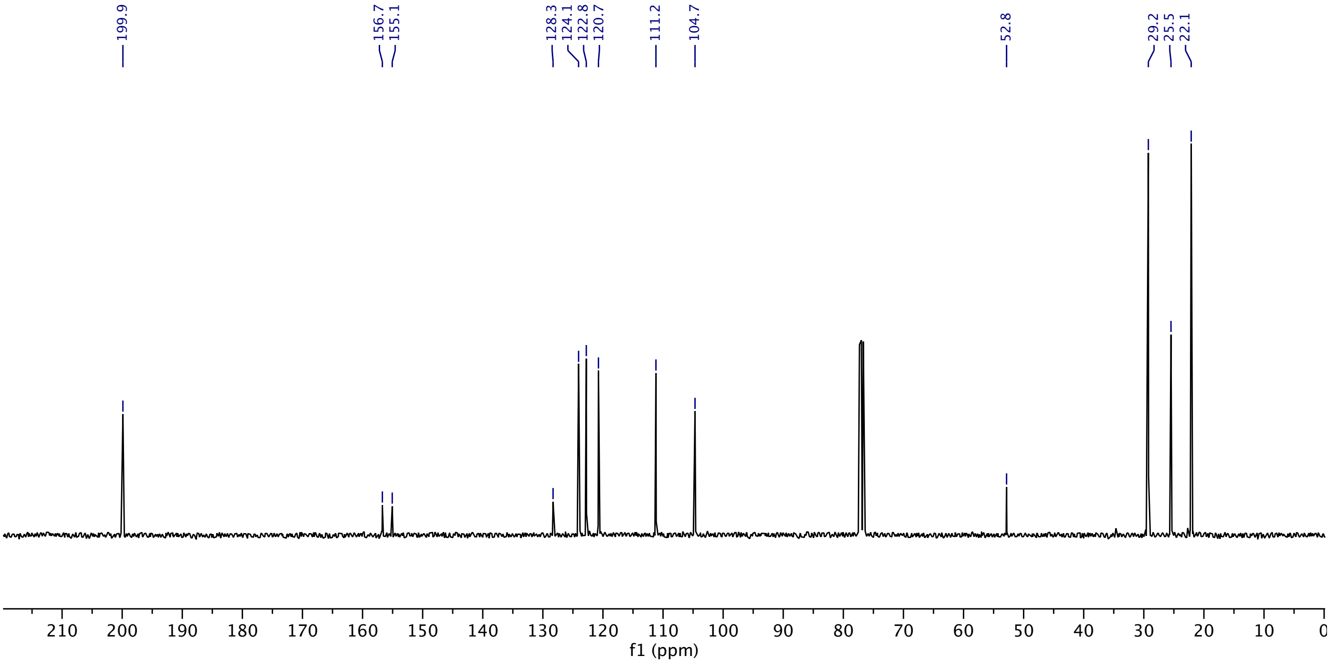

^{1}H NMR (400 MHz, CDCl$_3$)

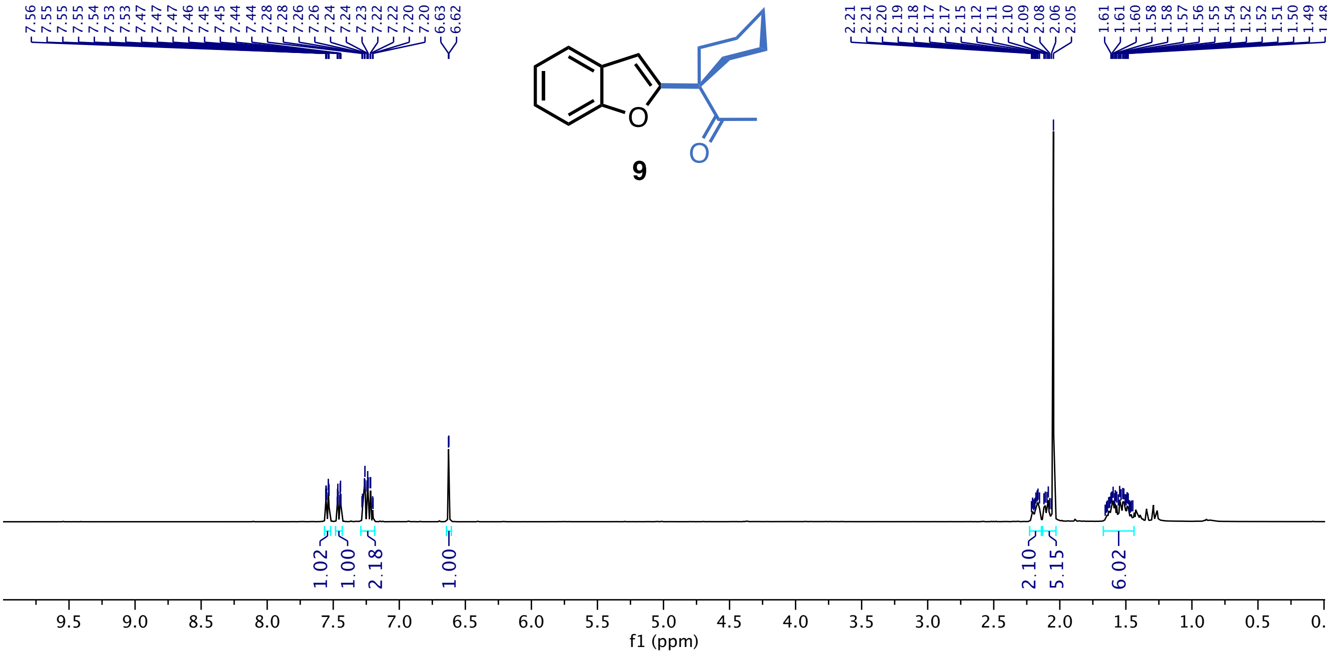

^{13}C NMR (100 MHz, CDCl$_3$)

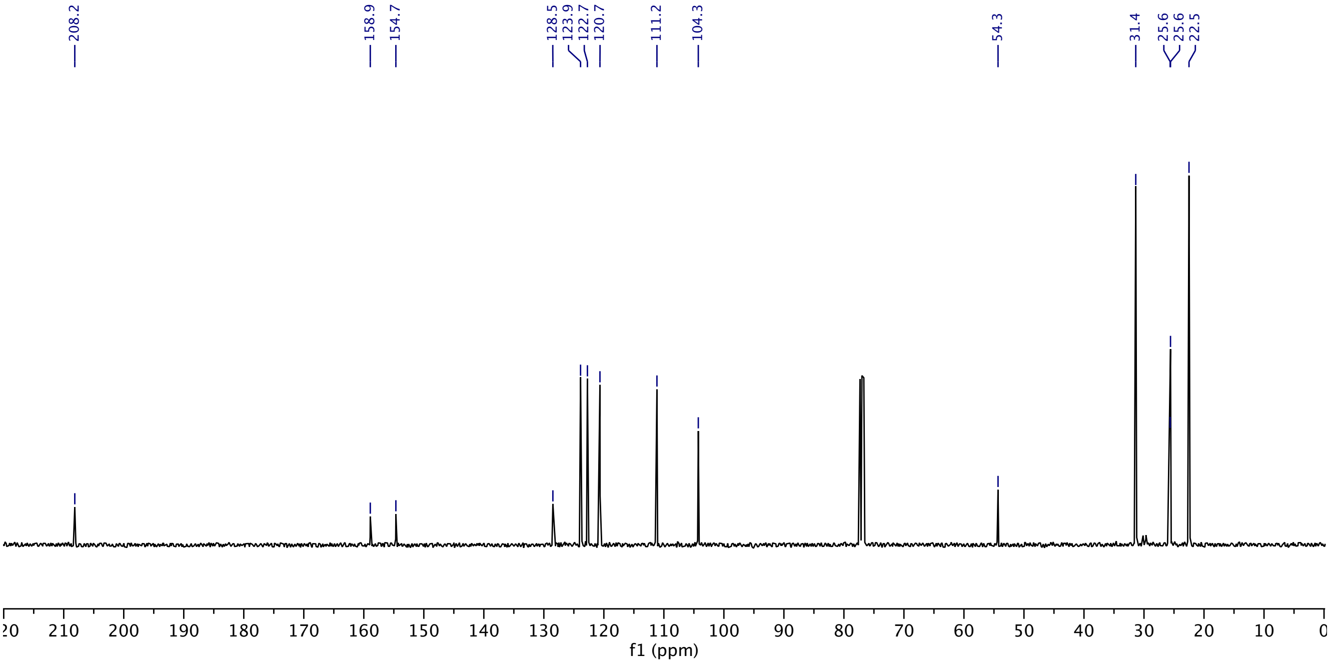

^{1}H NMR (400 MHz, $CDCl_3$)

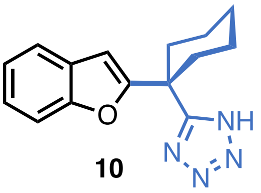

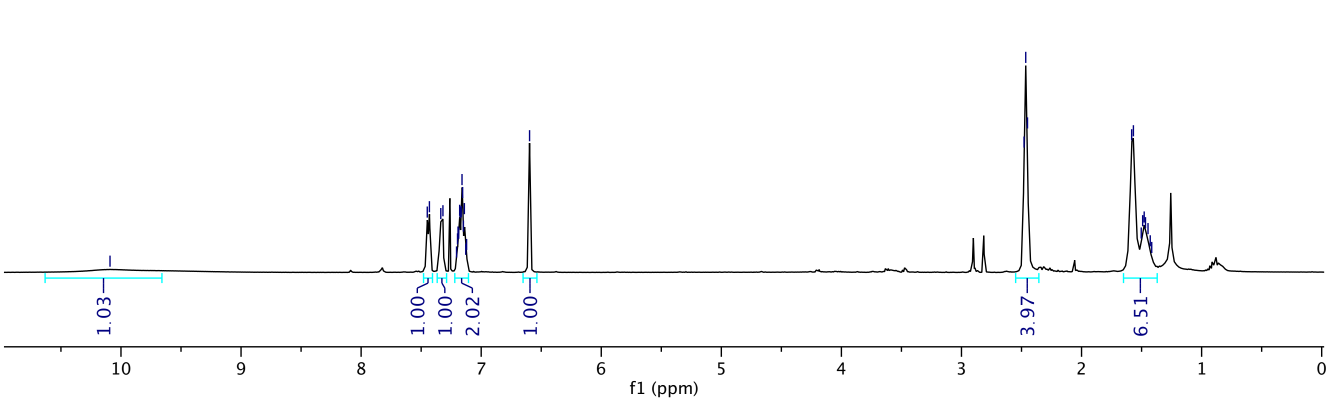

^{13}C NMR (100 MHz, $CDCl_3$)

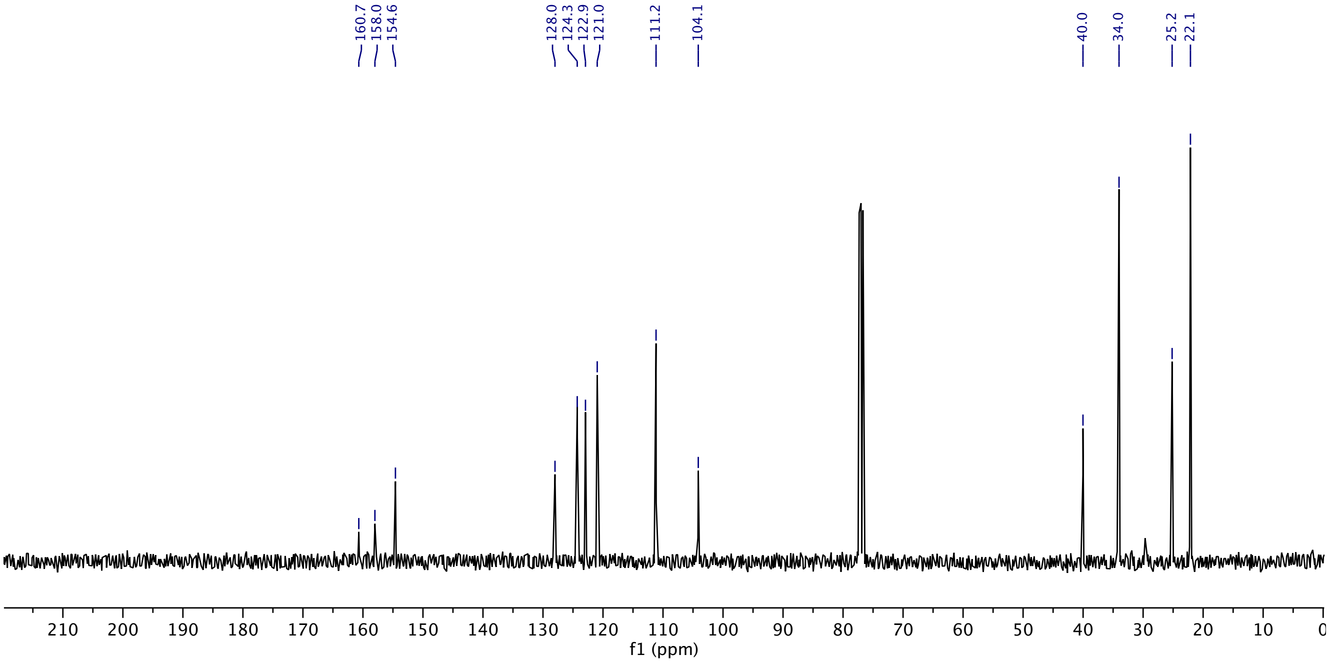

^{1}H NMR (500 MHz, $CDCl_3$)

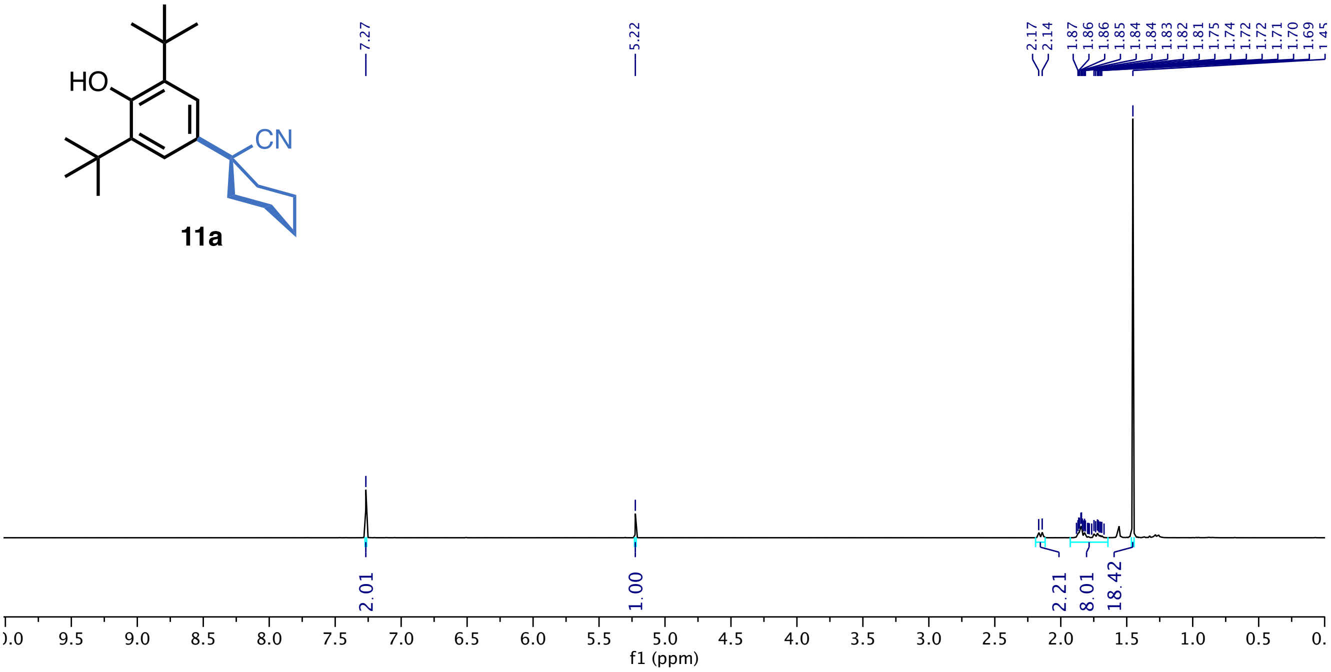

11a

^{13}C NMR (125 MHz, $CDCl_3$)

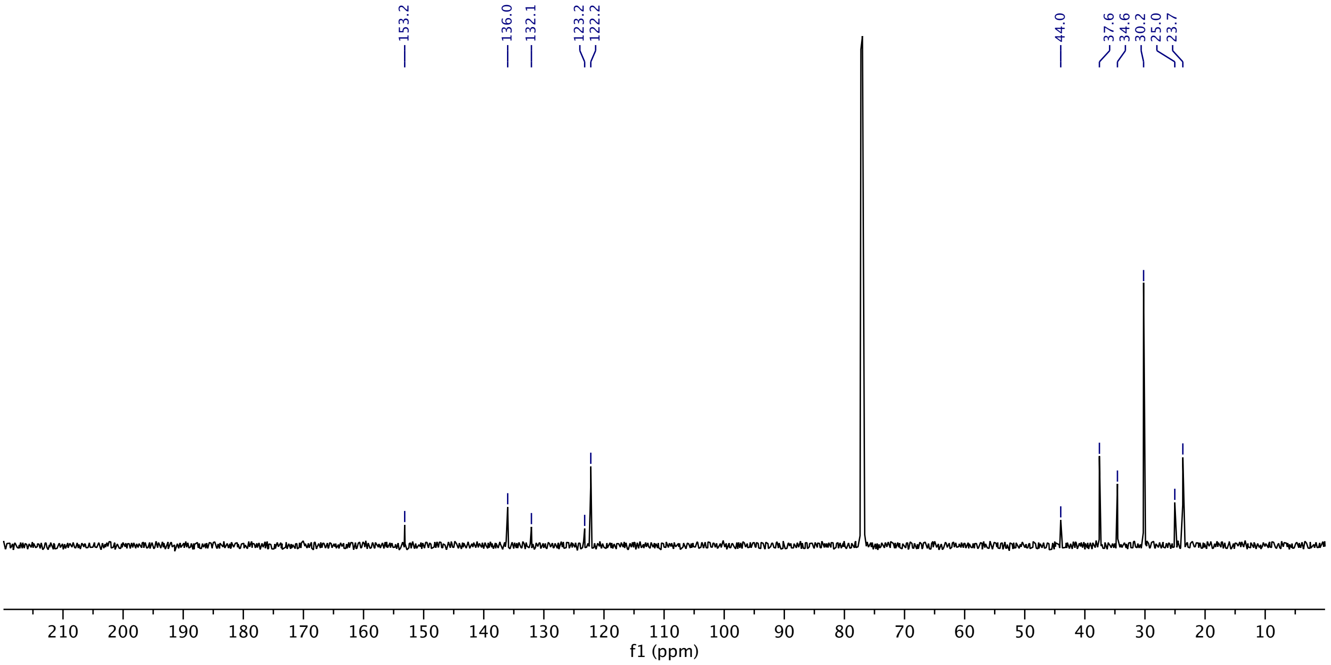

^{1}H NMR (400 MHz, $CDCl_3$)

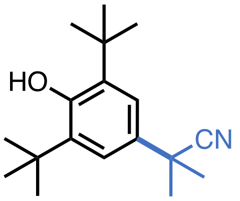

11b

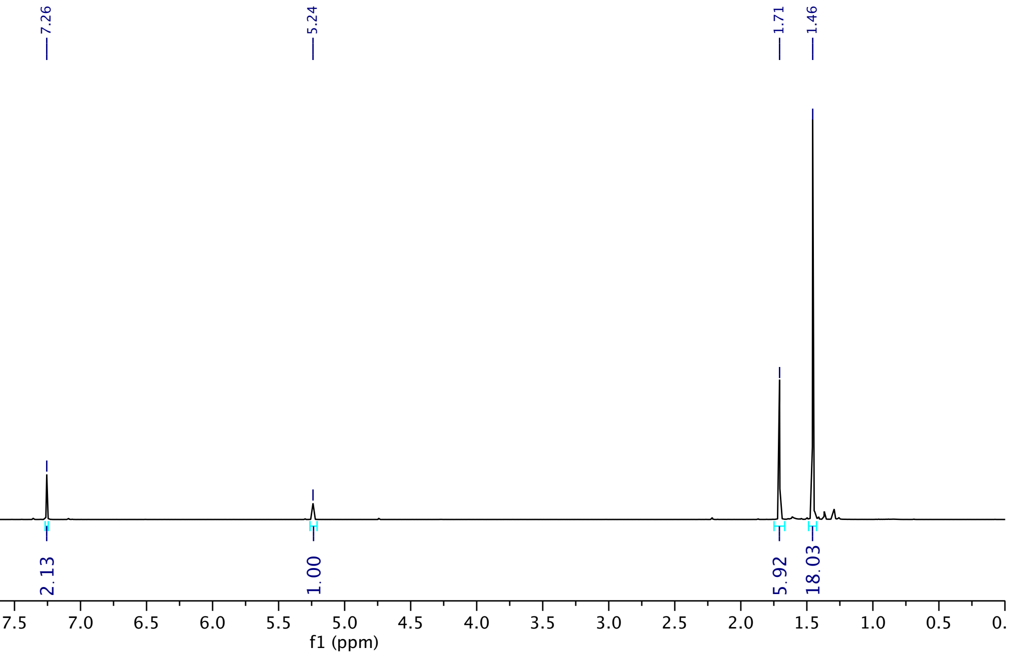

^{13}C NMR (100 MHz, $CDCl_3$)

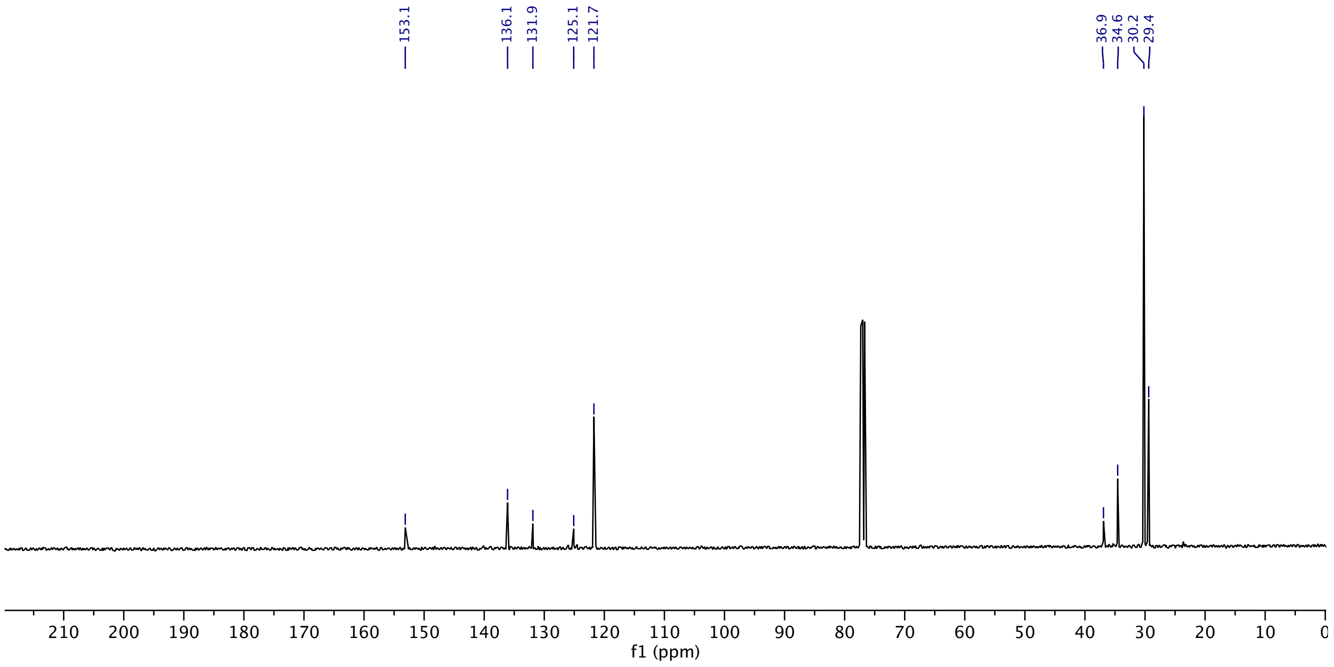

^{1}H NMR (400 MHz, $CDCl_3$)

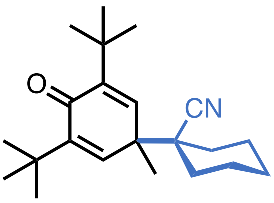

12a

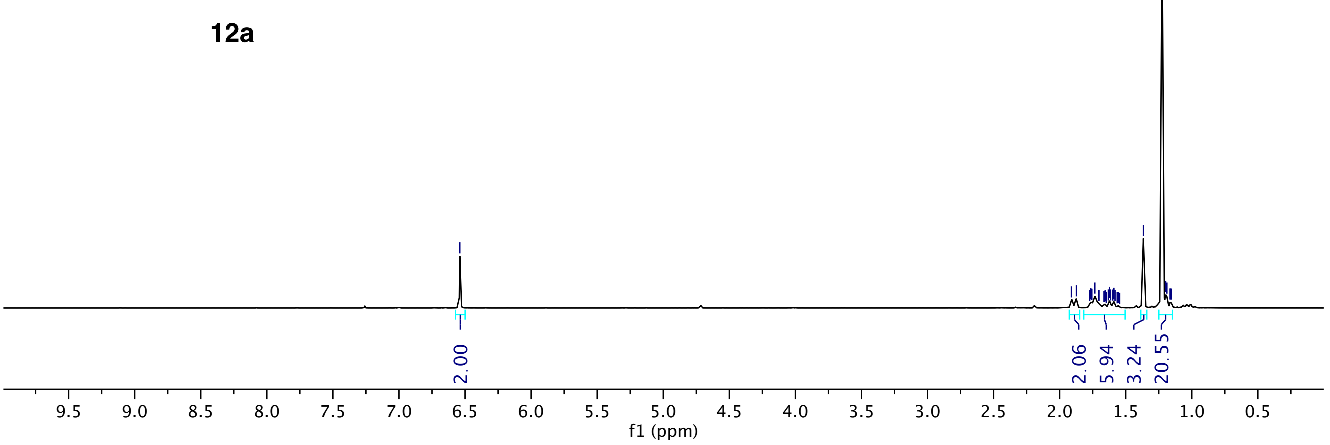

^{13}C NMR (100 MHz, $CDCl_3$)

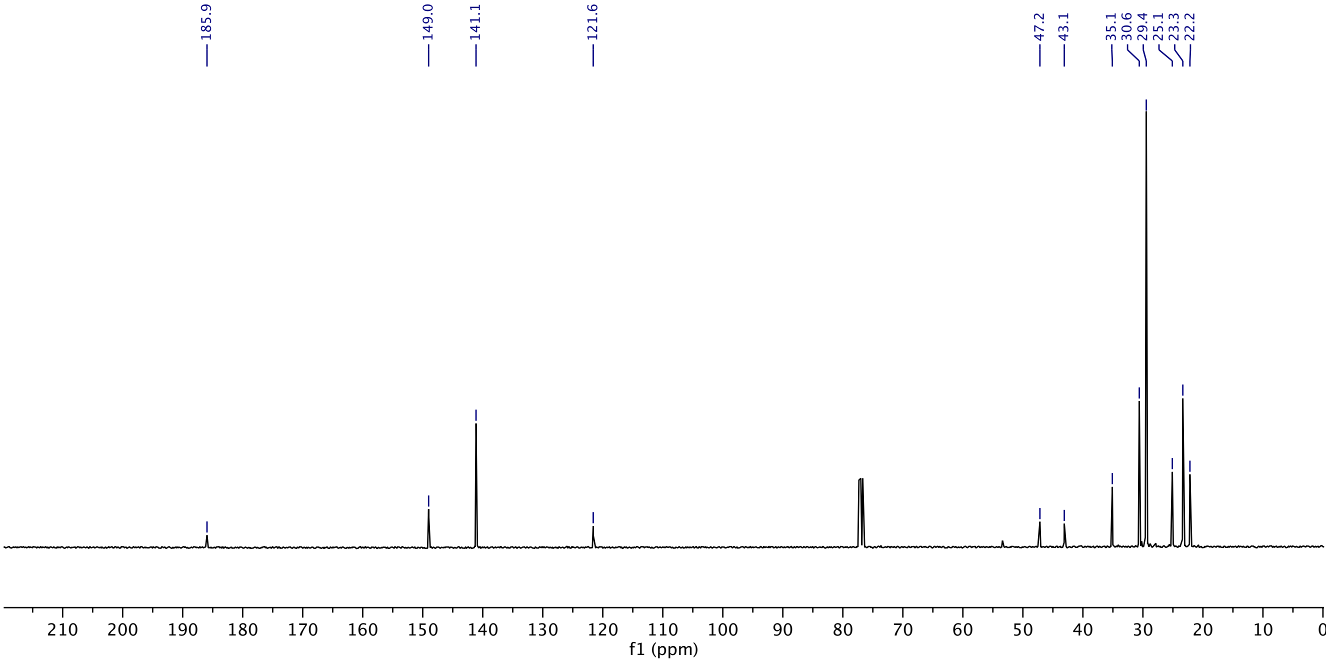

^{1}H NMR (400 MHz, $CDCl_3$)

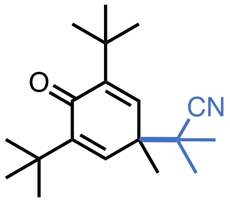

12b

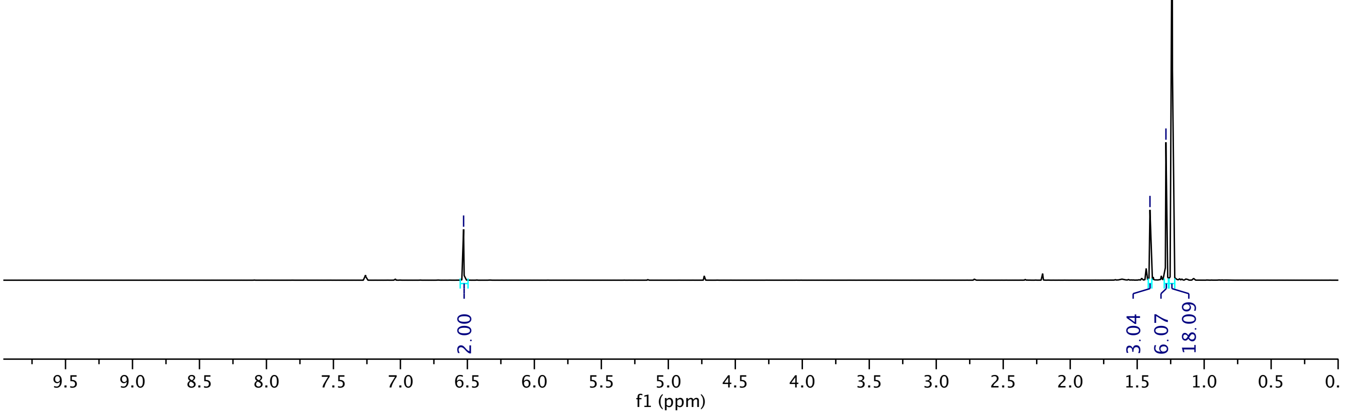

^{13}C NMR (100 MHz, $CDCl_3$)

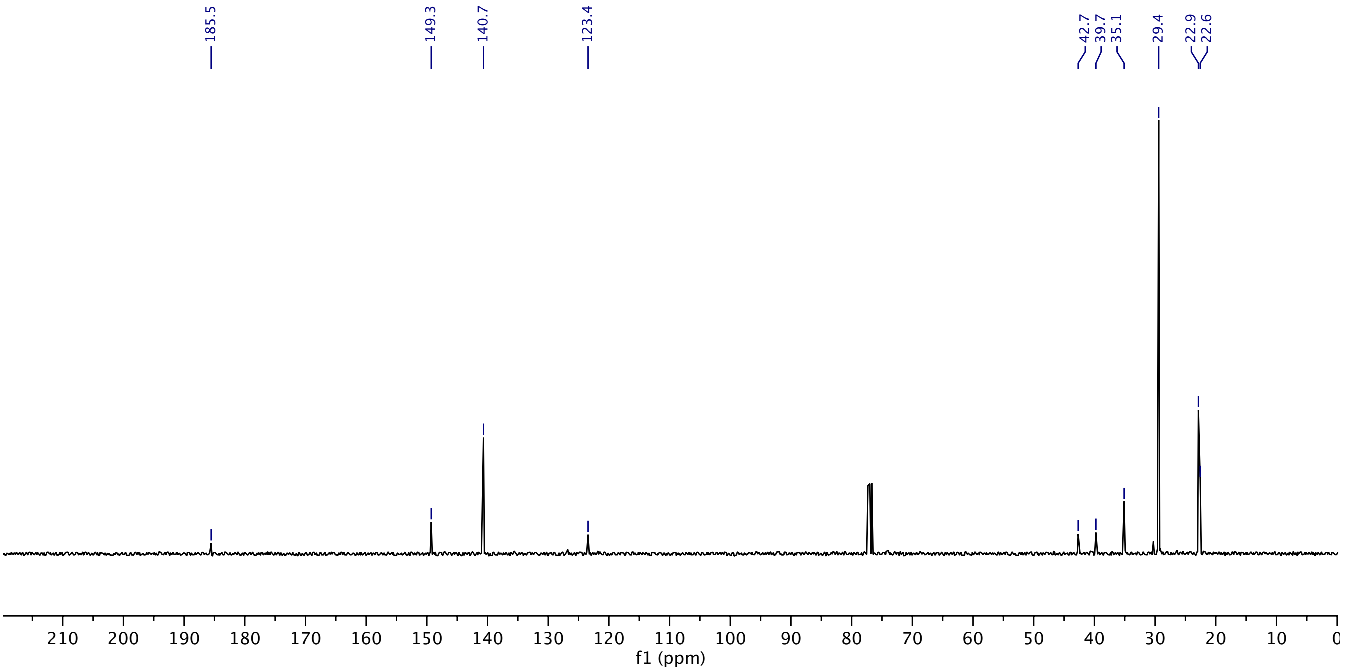

References

[1] R. Shishido, I. Sasaki, T. Seki, T. Ishiyama, H. Ito, *Chem. Eur. J.* **2019**, *25*, 12924–12928.
[2] G.-B. Liang, C. Zhou, H. Wang, X. Huo (Merck Sharp & Dohme Corp), U.S. patent WO2015095261A1, 2015.
[3] K. Kubota, Y. Pang, A. Miura, H. Ito, *Science* **2019**, *366*, 1500–1504.
[4] S. Biswas, V. Singh, S. Batra, *Tetrahedron* **2010**, *66*, 7781–7786.
[5] J.-M. Wang, T. Chen, C.-S. Yao, K. Zhang, *Org. Lett.* **2023**, *25*, 3325–3329.
[6] W.-L. Peng, Y.-J. Jhang, C.-Y. Chang, P.-K. Peng, W.-T. Zhaoa, Y.-K. Wu, *Org. Biomol. Chem.* **2022**, *20*, 6193–6195.
[7] N. T. Kadunce, S. E. Reisman, *J. Am. Chem. Soc.* **2015**, *137*, 10480–10483.
[8] L. Chen, M. Pu, S. Li, X. Sang, X. Liu, Y.-D. Wu, X. Feng, *J. Am. Chem. Soc.* **2021**, *143*, 19091–19098.
[9] H. Yoneyama, Y. Usami, S. Komeda, S. Harusawa, *Synthesis* **2013**, *45*, 1051–1059.
[10] Bruker (2018). APEX3. Bruker AXS Inc., Madison, Wisconsin, USA.
[11] G. M. Sheldrick, *Acta Cryst.* **2008**, *A64*, 112–122.
[12] G. M. Sheldrick, *Acta Cryst.* **2015**, *A71*, 3–8.
[13] G. M. Sheldrick, *Acta Cryst.* **2015**, *C71*, 3–8.
[14] L. J. Farrugia, *J. Appl. Cryst.* **2012**, *45*, 849–854.
